# Supplementary material for: Biocatalytic trifluoromethylation of unprotected phenols
Source: Nat Commun. 2016 Nov 11;7:13323. doi: 10.1038/ncomms13323 (PMC5114620; doi:10.1038/ncomms13323)
Supplement: Supplementary Information — Supplementary Figures 1-55, Supplementary Tables 1-21, Supplementary Methods, and Supplementary References. [file ncomms13323-s1.pdf]

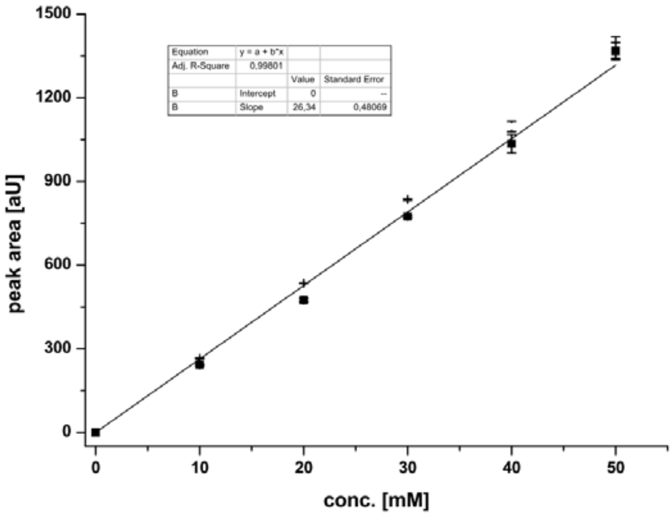

Supplementary Figure 1: Calibration curve of **1a** [1-(4-hydroxy-3,5-dimethoxyphenyl)ethanone]; determination of peak area was performed in duplicate.

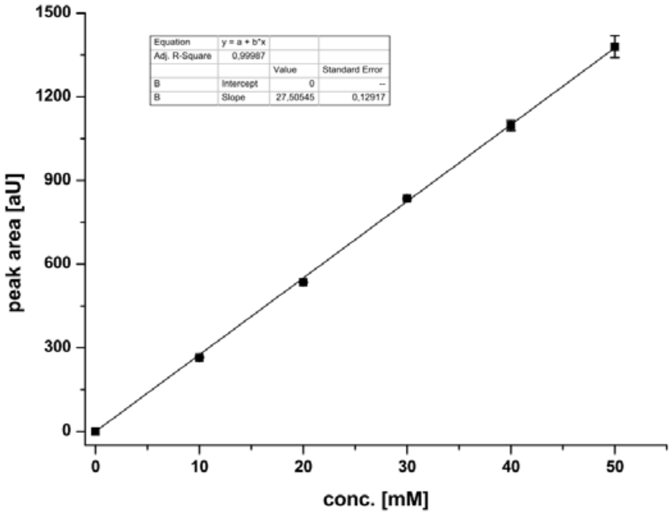

Supplementary Figure 2: Calibration curve of **2a** [1-(4-hydroxy-3,5-dimethoxy-2-(trifluoromethyl)phenyl)ethanone]; determination of peak area was performed in duplicate.

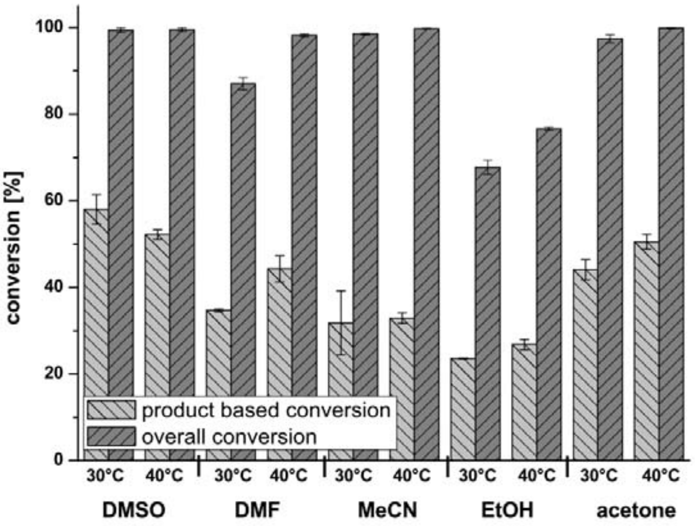

Supplementary Figure 3: Reagents and conditions: sodium acetate buffer (250 mM, pH 5.5), 50 mM substrate **1a** (4.91 mg per 500 µL), 2.0 eq. $Zn(SO_2CF_3)_2$ = (16.6 mg per 500 µL), 8 eq. *t*BuOOH (aq. 70% wt solution: 27.48 µL per 500µL); Laccase (2.5 mg per 500 µL), reaction time 24 hours, 30 °C, 900 rpm in Eppendorf orbital shaker (horizontal position), with syringe needle for air exchange in the top lid of the Eppendorf tube (2.0 mL).

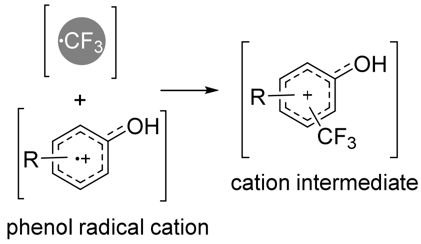

Supplementary Figure 4. C–C bond formation between the CF_3 radical and the phenol radical cation to give the corresponding substituted cationic phenol intermediate.

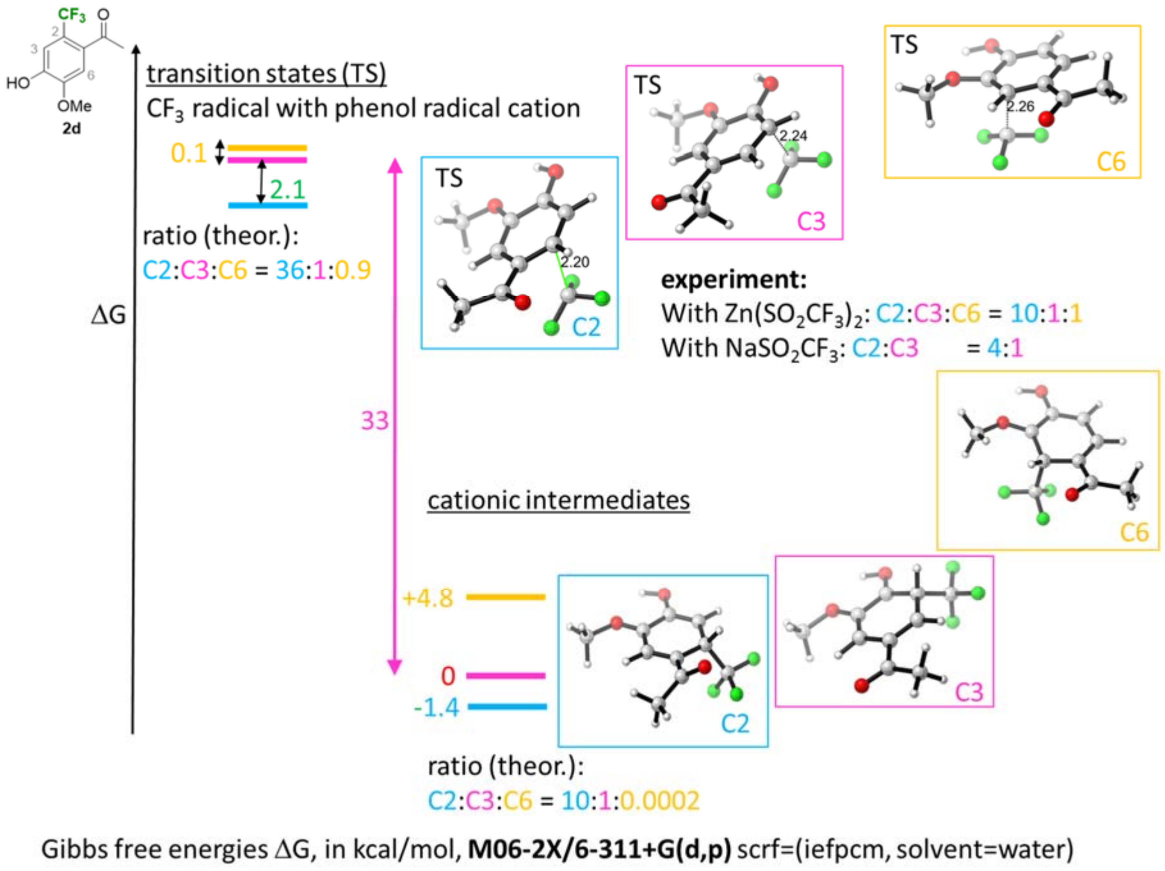

Supplementary Figure 5. Energy diagram and structures of transition states and cationic intermediates of reaction leading preferentially to **2d**. The transition state is for the reaction in **Supplementary Figure 4.**

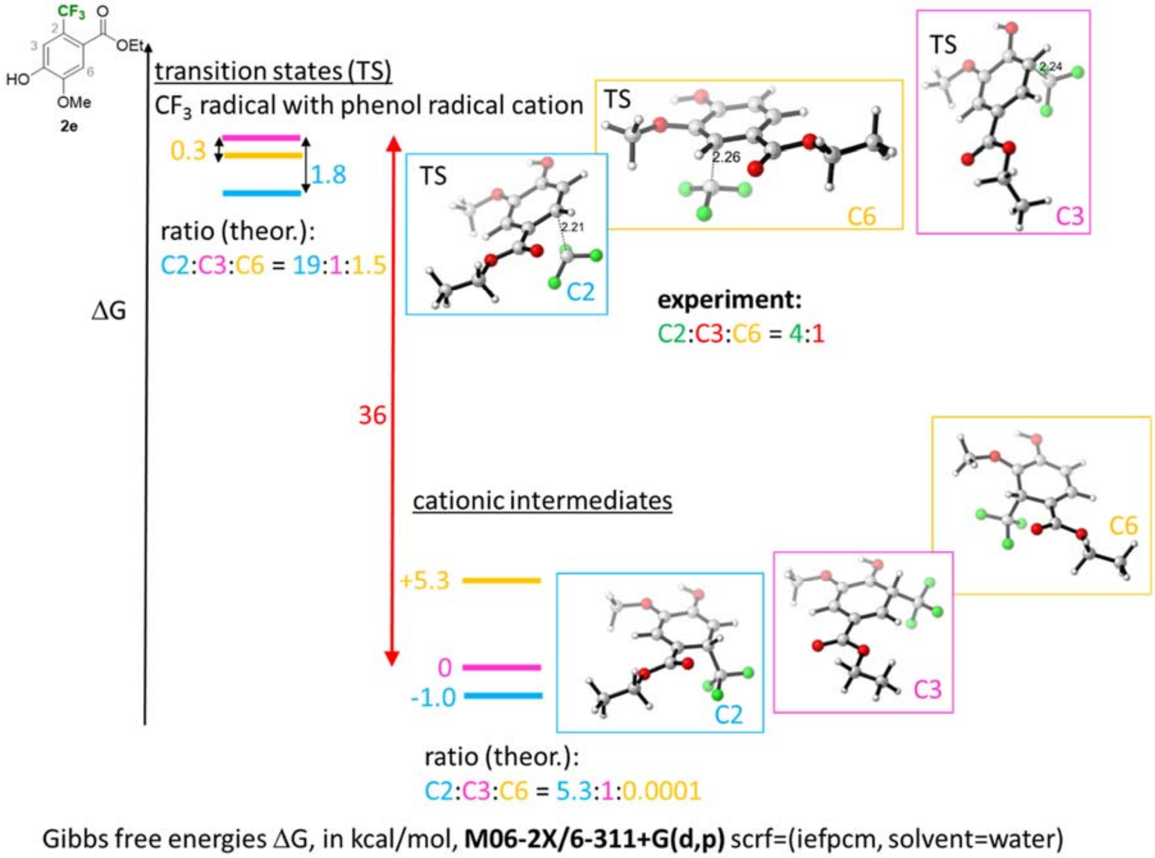

Supplementary Figure 6. Energy diagram and structures of transition states and cationic intermediates of reaction leading preferentially to **2e**. The transition state is for the reaction in **Supplementary Figure 4.**

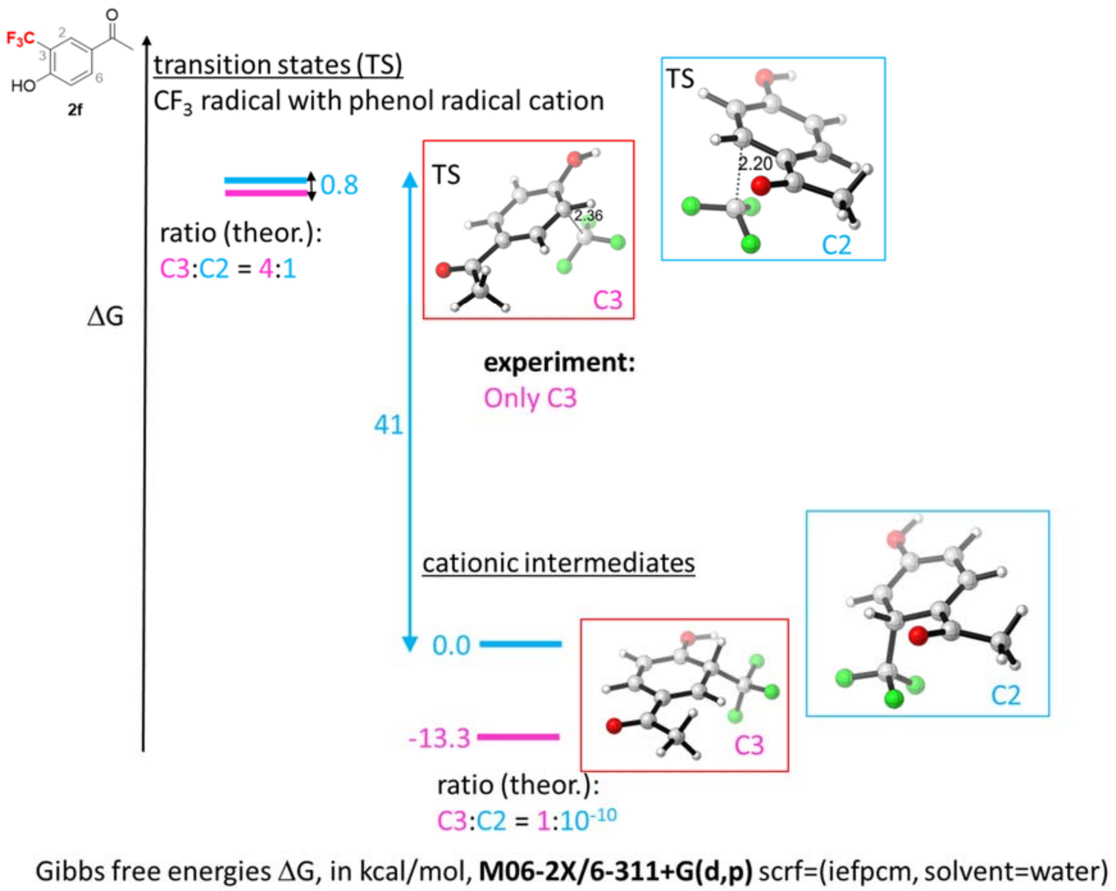

Supplementary Figure 7. Energy diagram and structures of transition states and cationic intermediates of reaction leading preferentially to **2f**. The transition state is for the reaction in **Supplementary Figure 4.**

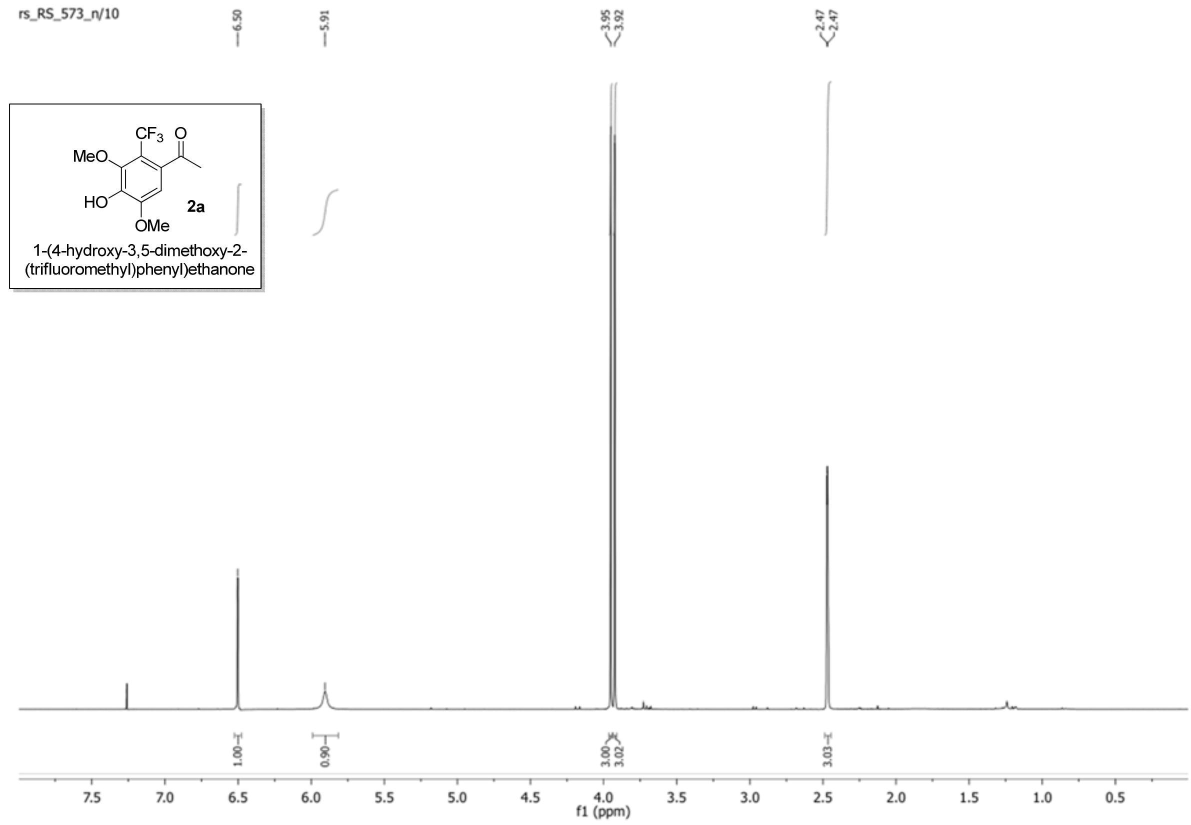

Supplementary Figure 8. ^{1}H-NMR of 1-(4-hydroxy-3,5-dimethoxy-2-(trifluoromethyl)phenyl)ethanone (**2a**)

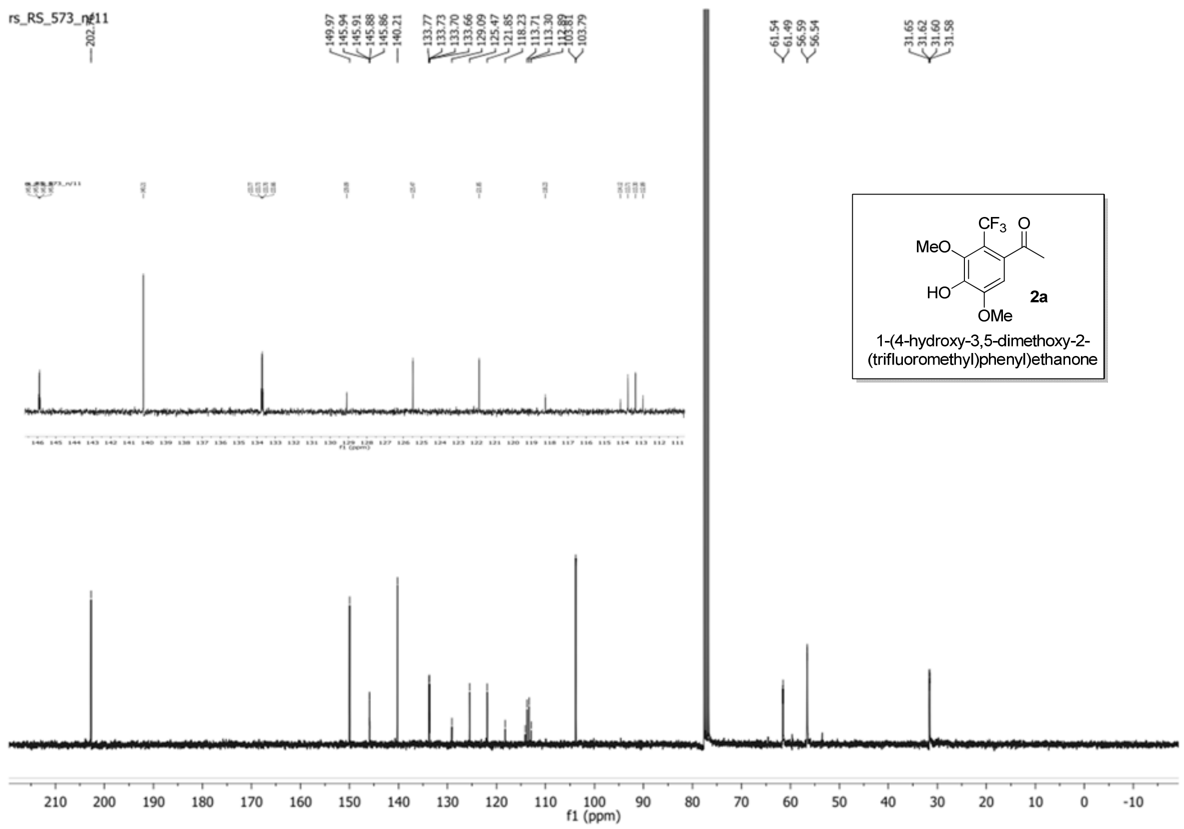

Supplementary Figure 9. ^{13}C-NMR of 1-(4-hydroxy-3,5-dimethoxy-2-(trifluoromethyl)phenyl)ethanone (**2a**)

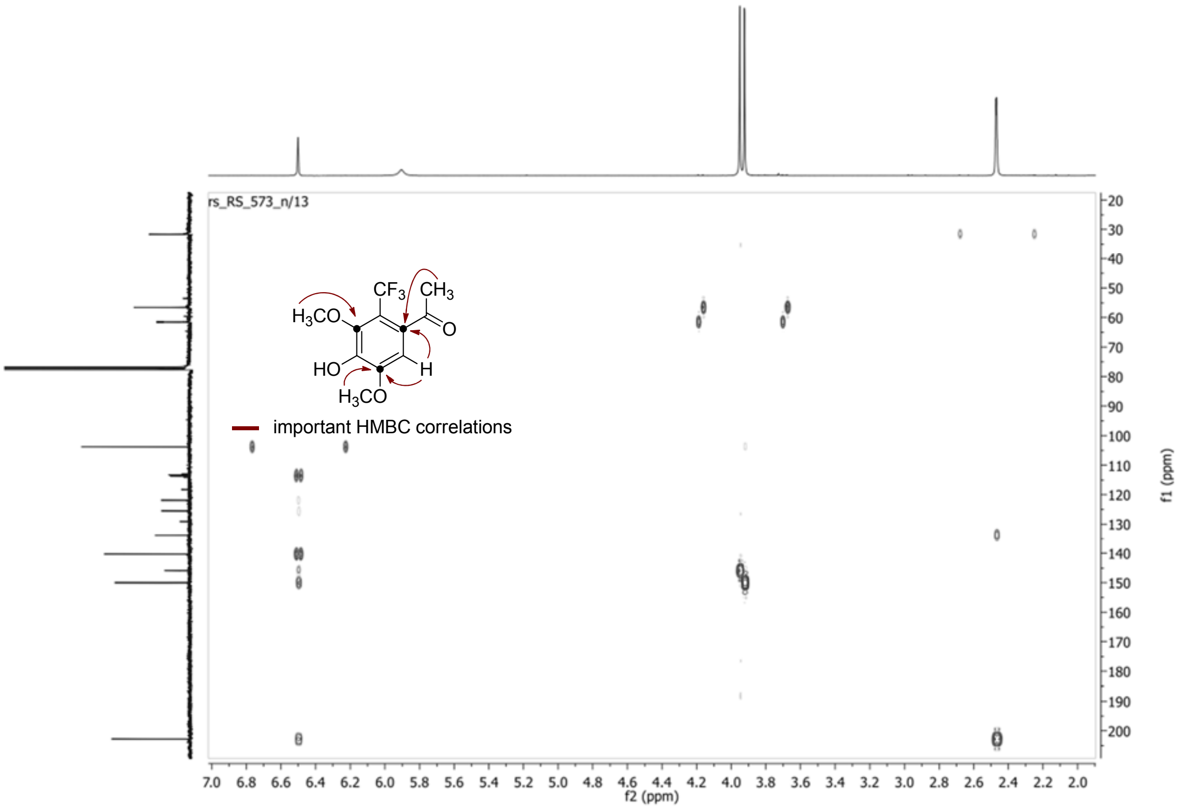

Supplementary Figure 10. HMBC NMR of 1-(4-hydroxy-3,5-dimethoxy-2-(trifluoromethyl)phenyl)ethanone (**2a**)

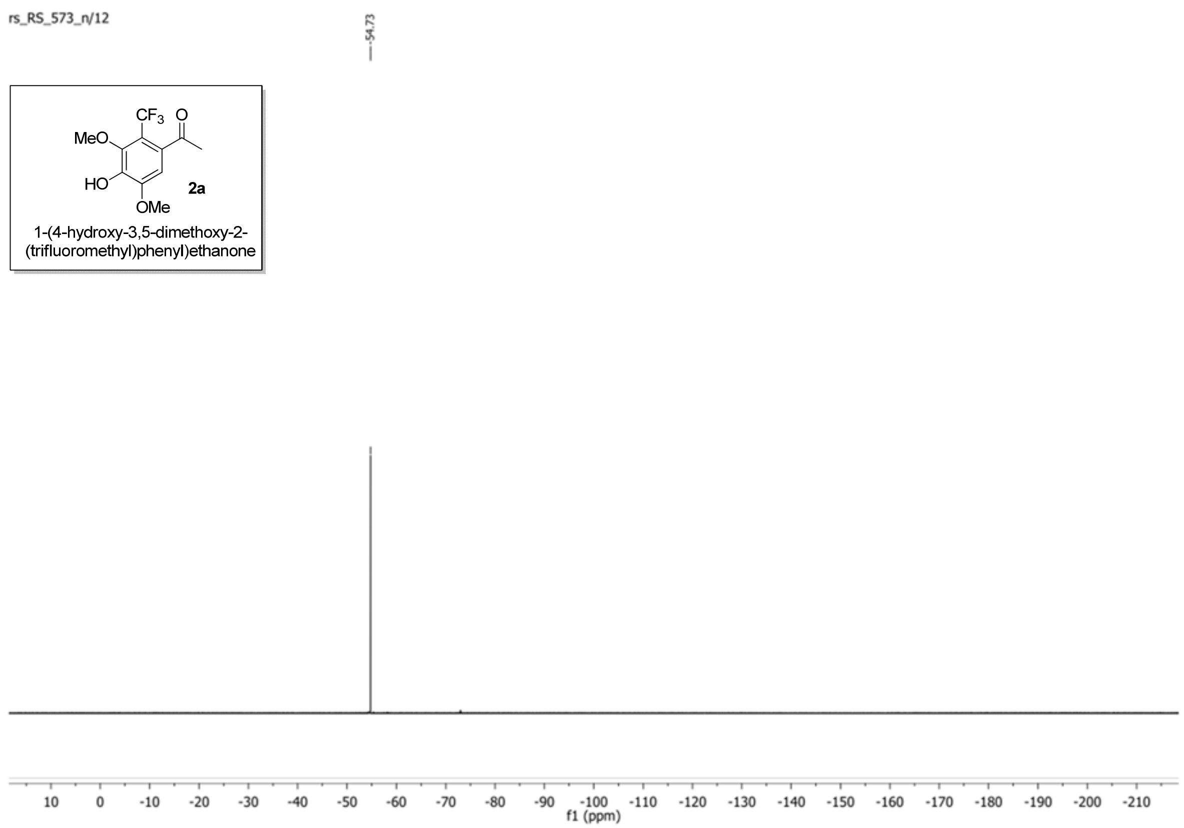

Supplementary Figure 11. ^{19}F-NMR of 1-(4-hydroxy-3,5-dimethoxy-2-(trifluoromethyl)phenyl)ethanone (**2a**)

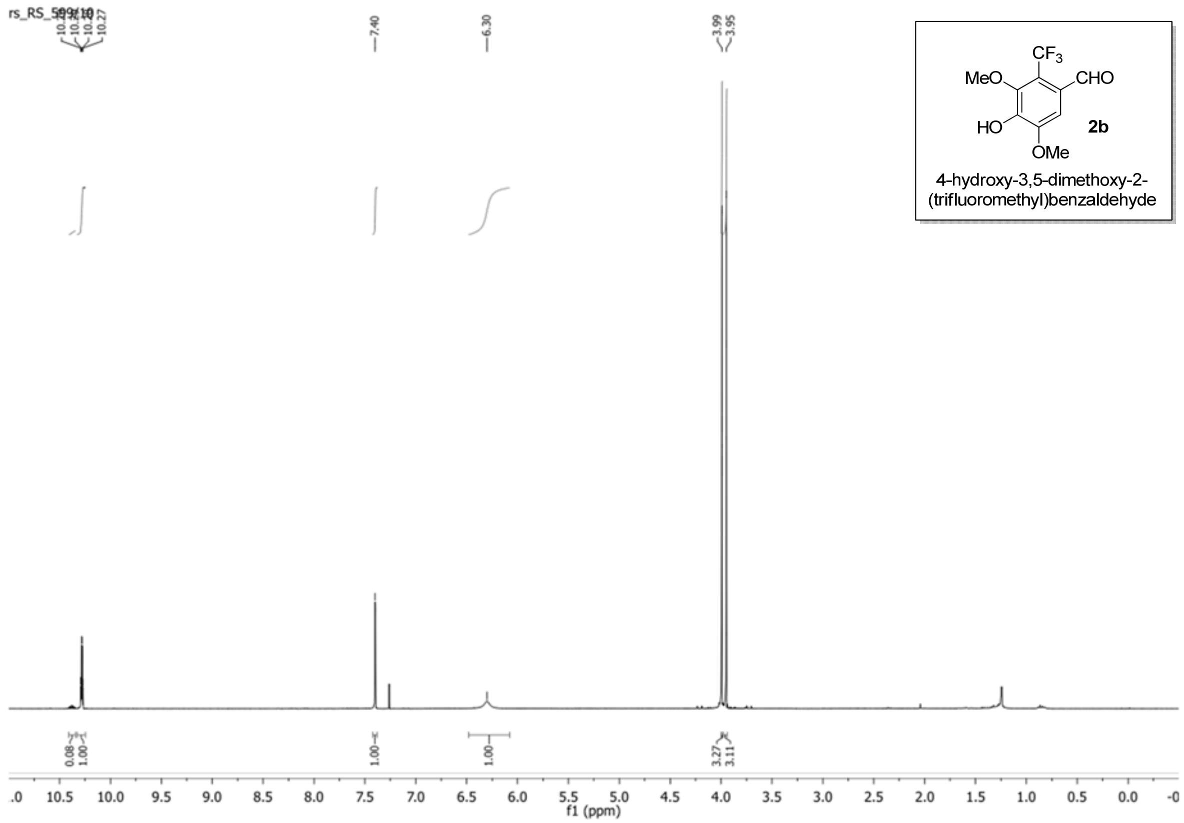

Supplementary Figure 12. ^{1}H-NMR of 4-hydroxy-3,5-dimethoxy-2-(trifluoromethyl)benzaldehyde (**2b**)

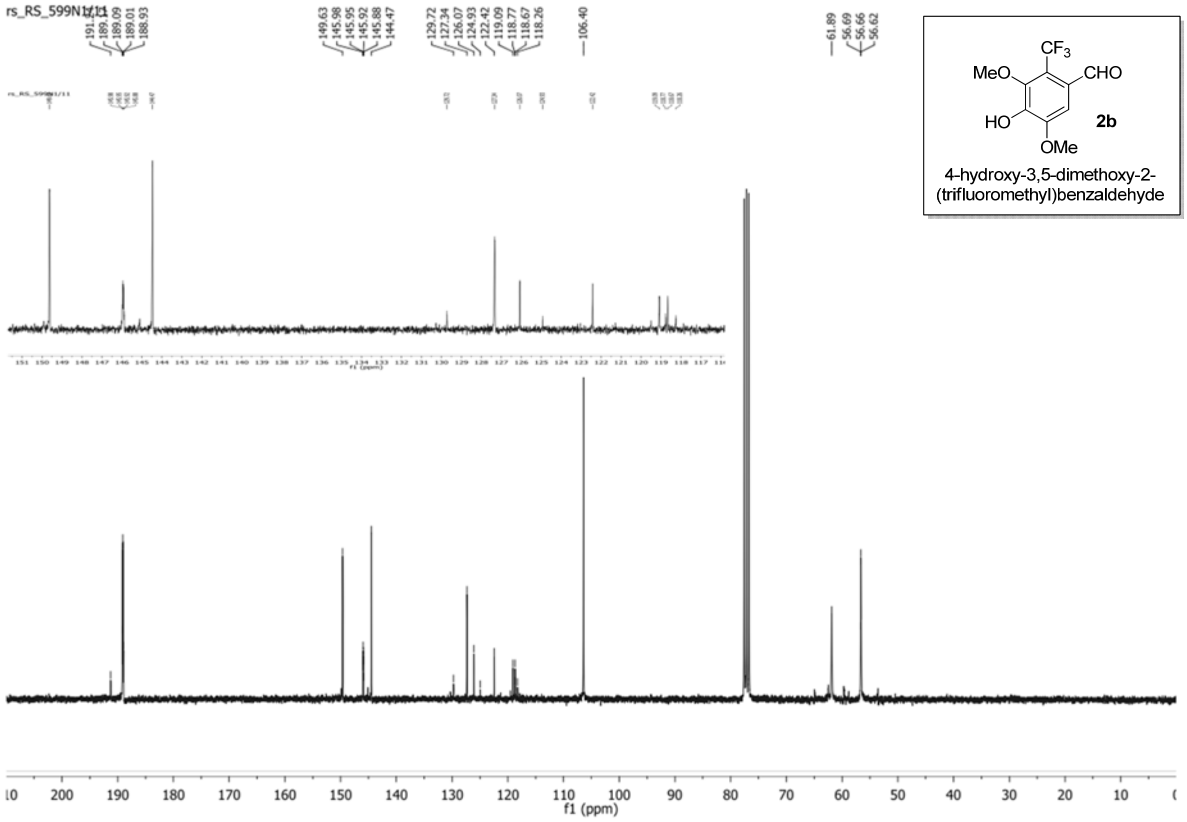

Supplementary Figure 13. ^{13}C-NMR of 4-hydroxy-3,5-dimethoxy-2-(trifluoromethyl)benzaldehyde (**2b**)

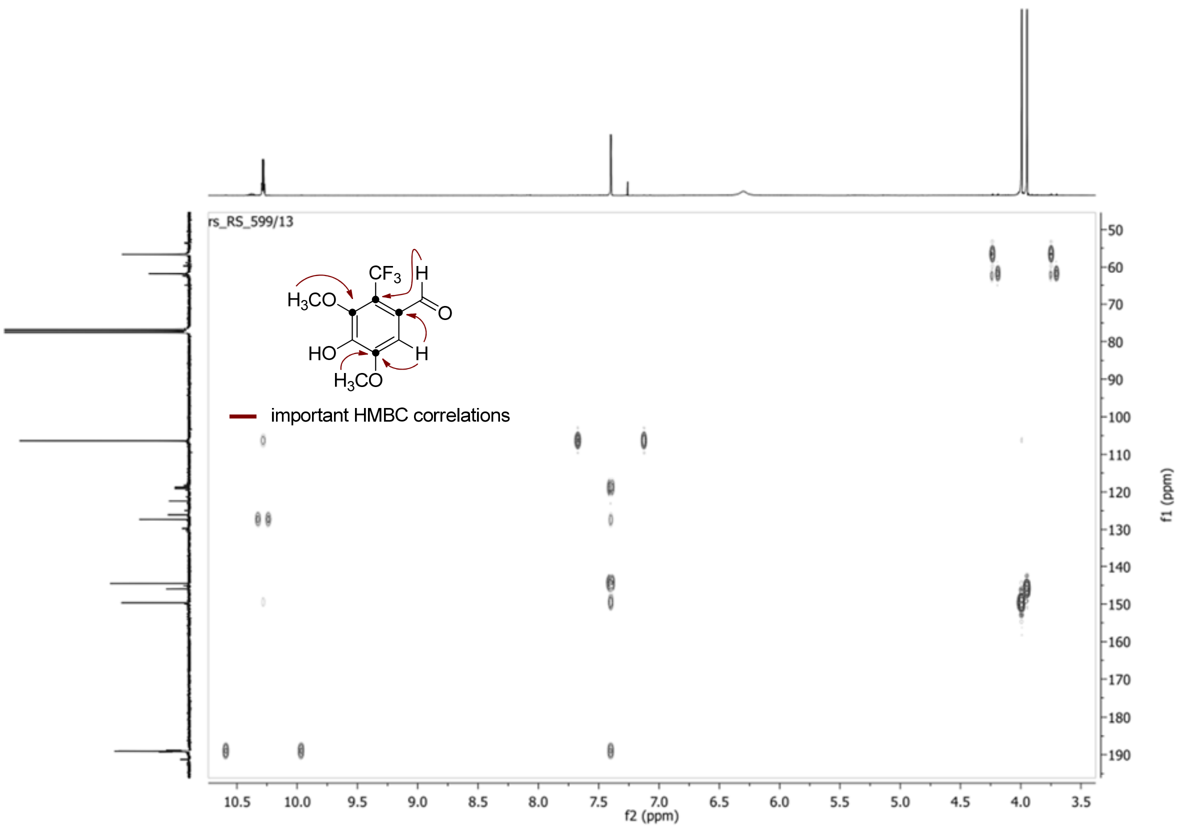

Supplementary Figure 14. HMBC of 4-hydroxy-3,5-dimethoxy-2-(trifluoroethyl)benzaldehyde (**2b**)

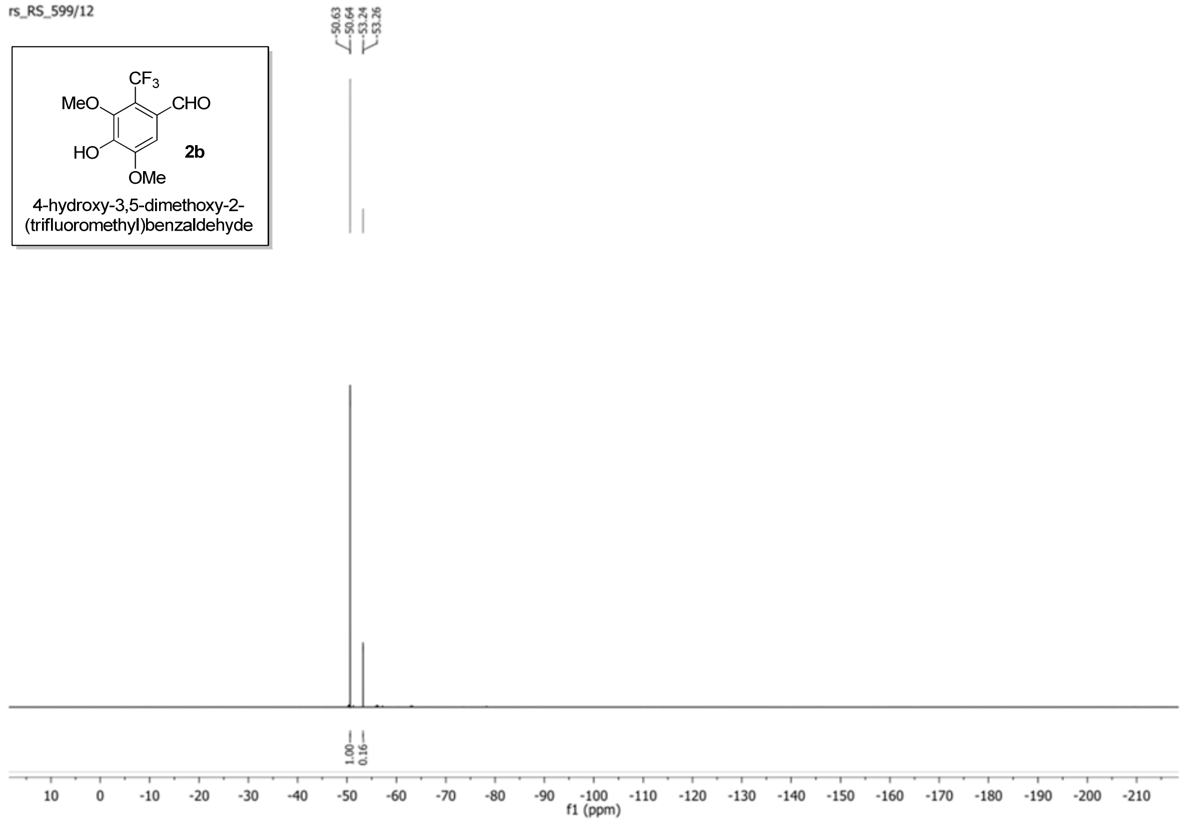

Supplementary Figure 15. ^{19}F-NMR of 4-hydroxy-3,5-dimethoxy-2-(trifluoromethyl)benzaldehyde (**2b**)

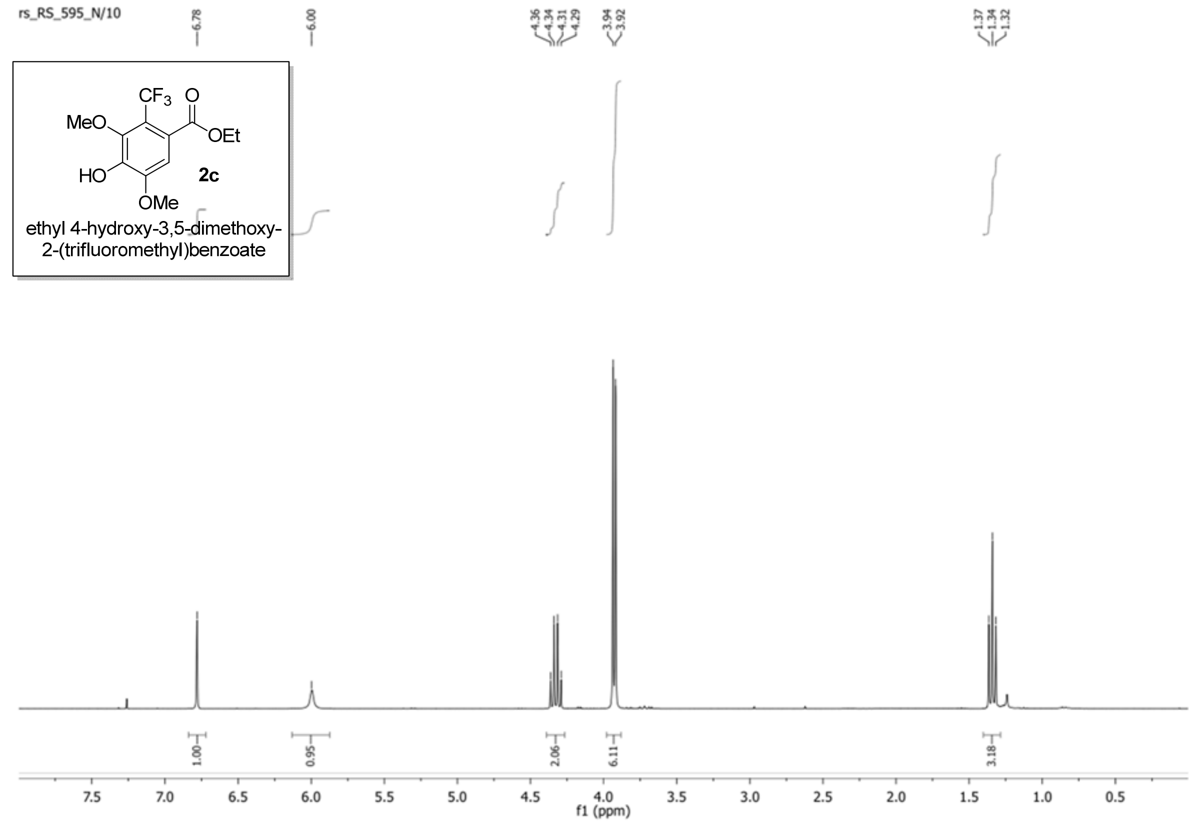

Supplementary Figure 16. ^{1}H-NMR of ethyl 4-hydroxy-3,5-dimethoxy-2-(trifluoromethyl)benzoate (**2c**)

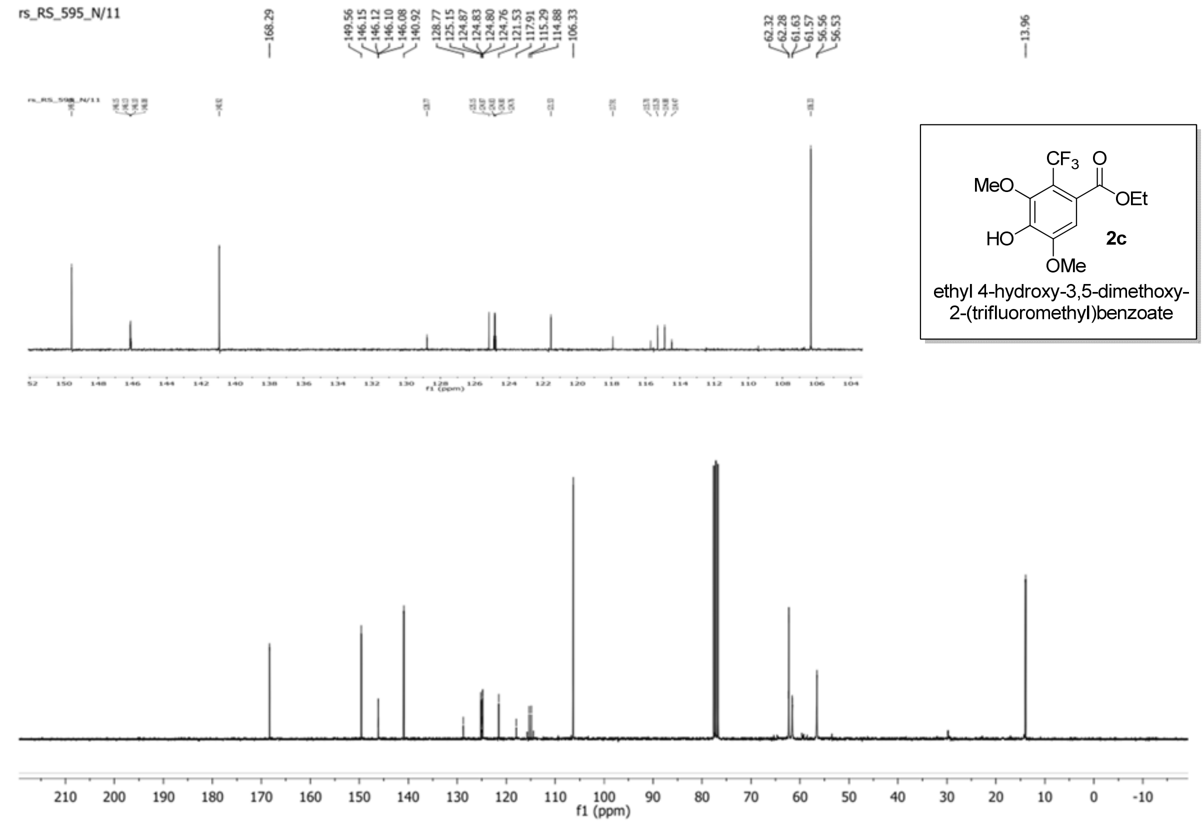

Supplementary Figure 17. ^{13}C-NMR of ethyl 4-hydroxy-3,5-dimethoxy-2-(trifluoromethyl)benzoate (**2c**)

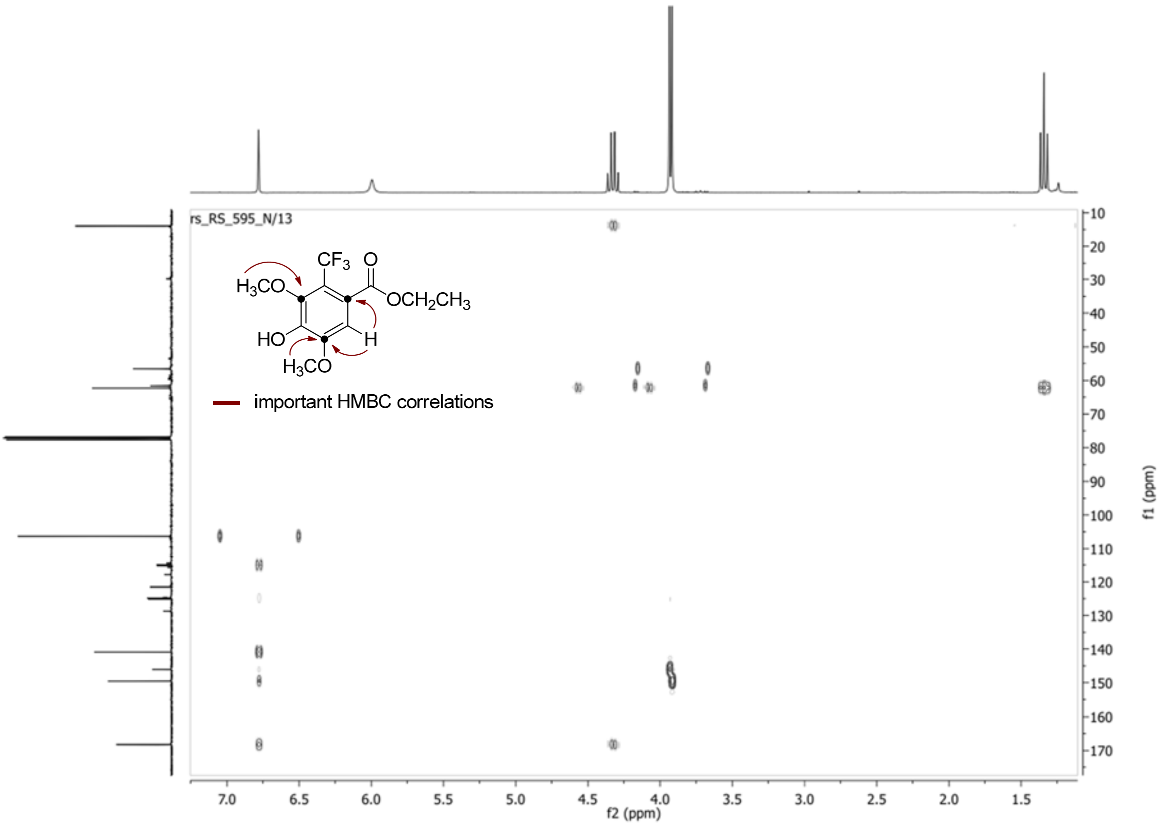

Supplementary Figure 18. HMBC of ethyl 4-hydroxy-3,5-dimethoxy-2-(trifluoromethyl)benzoate (**2c**)

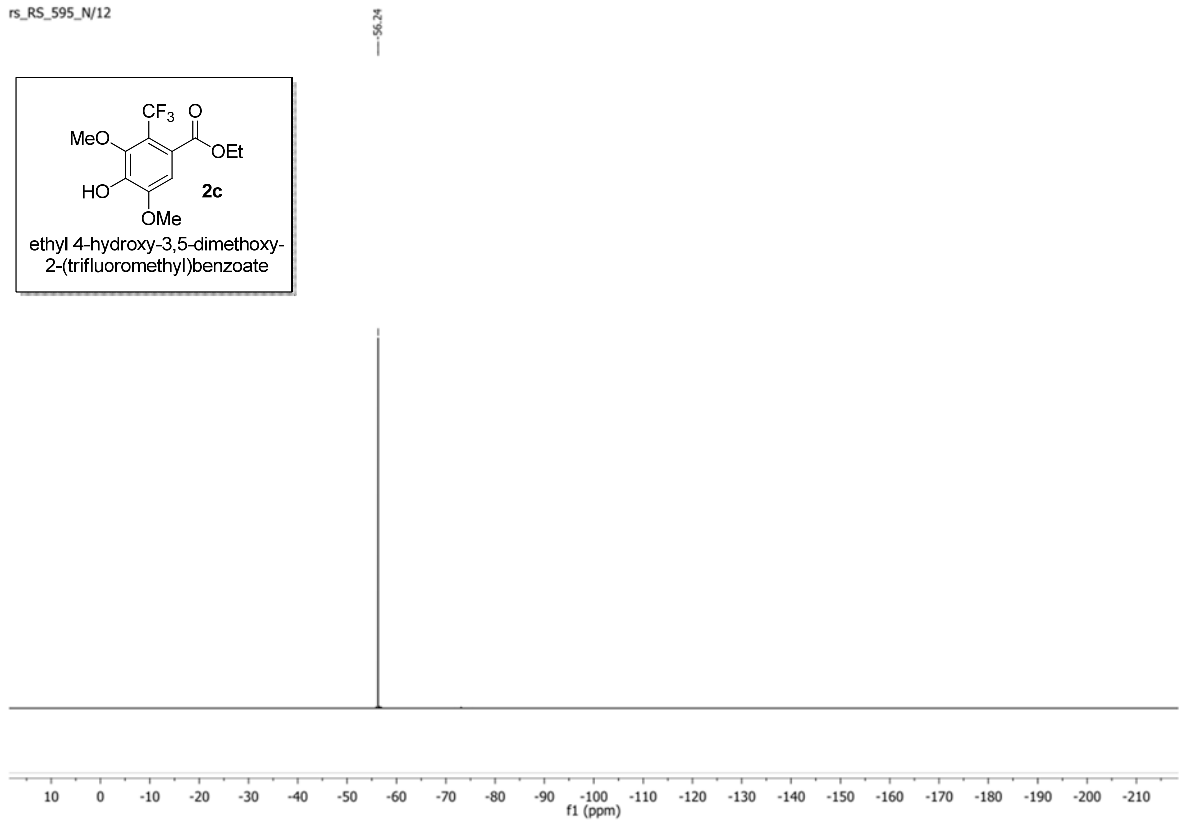

Supplementary Figure 19. ^{19}F-NMR ethyl 4-hydroxy-3,5-dimethoxy-2-(trifluoromethyl)benzoate (**2c**)

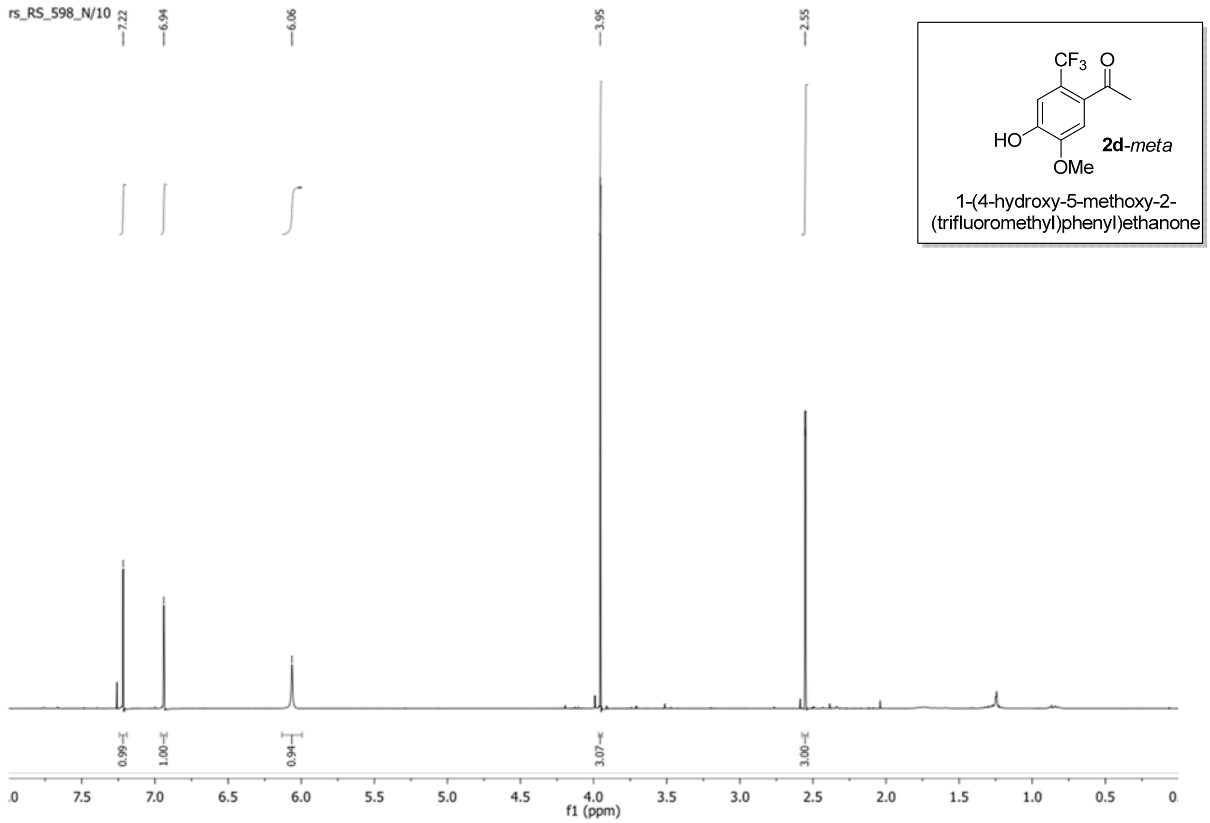

Supplementary Figure 20. ^{1}H-NMR of 1-(4-hydroxy-5-methoxy-2-(trifluoromethyl)phenyl)ethanone (**2d**-*meta*)

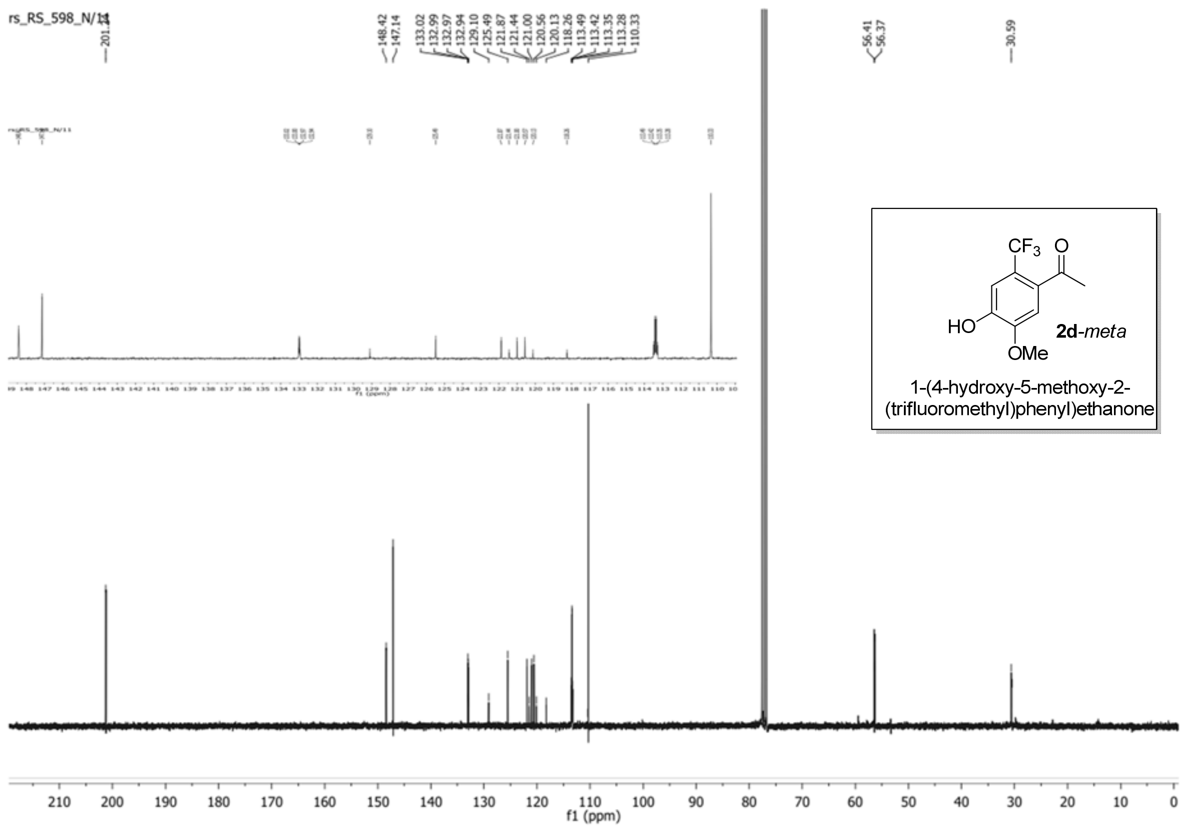

Supplementary Figure 21. ^{13}C-NMR of of 1-(4-hydroxy-5-methoxy-2-(trifluoromethyl)phenyl)ethanone (**2d**-*meta*)

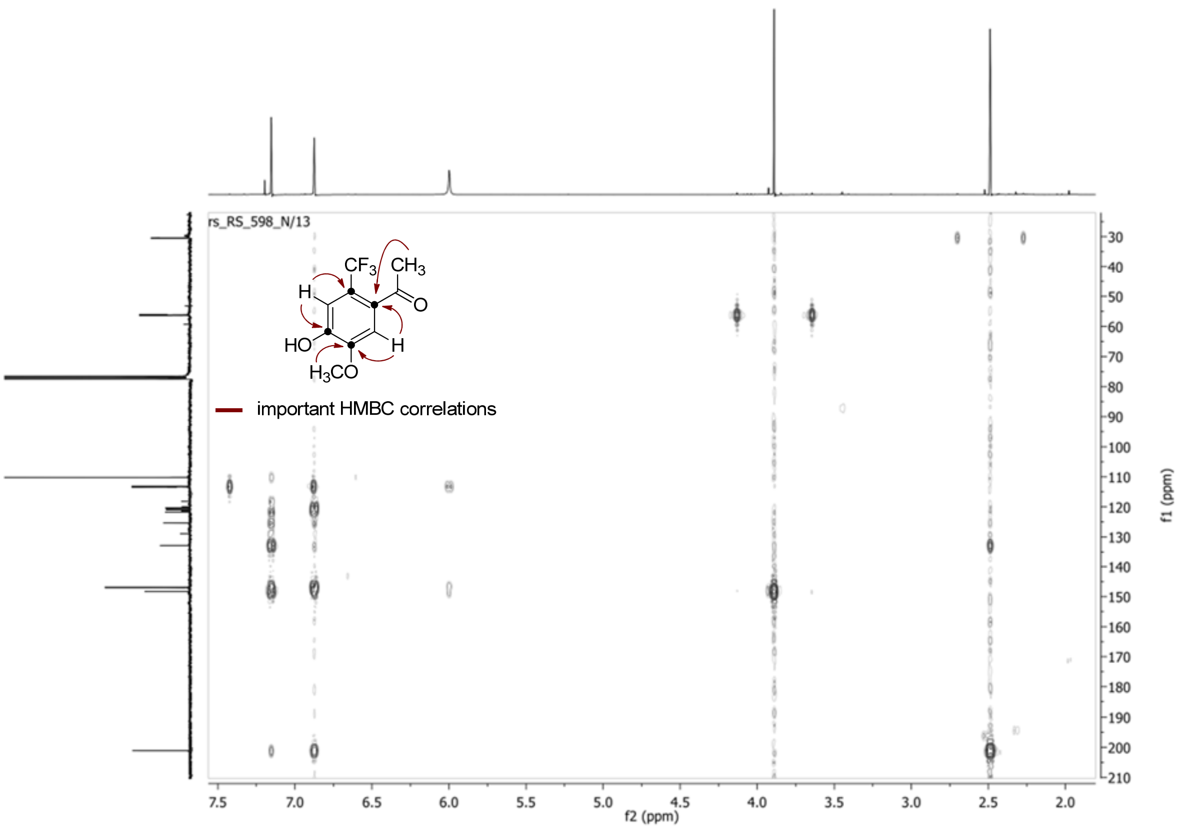

Supplementary Figure 22. HMBC of 1-(4-hydroxy-5-methoxy-2-(trifluoromethyl)phenyl)ethanone (**2d**-*meta*)

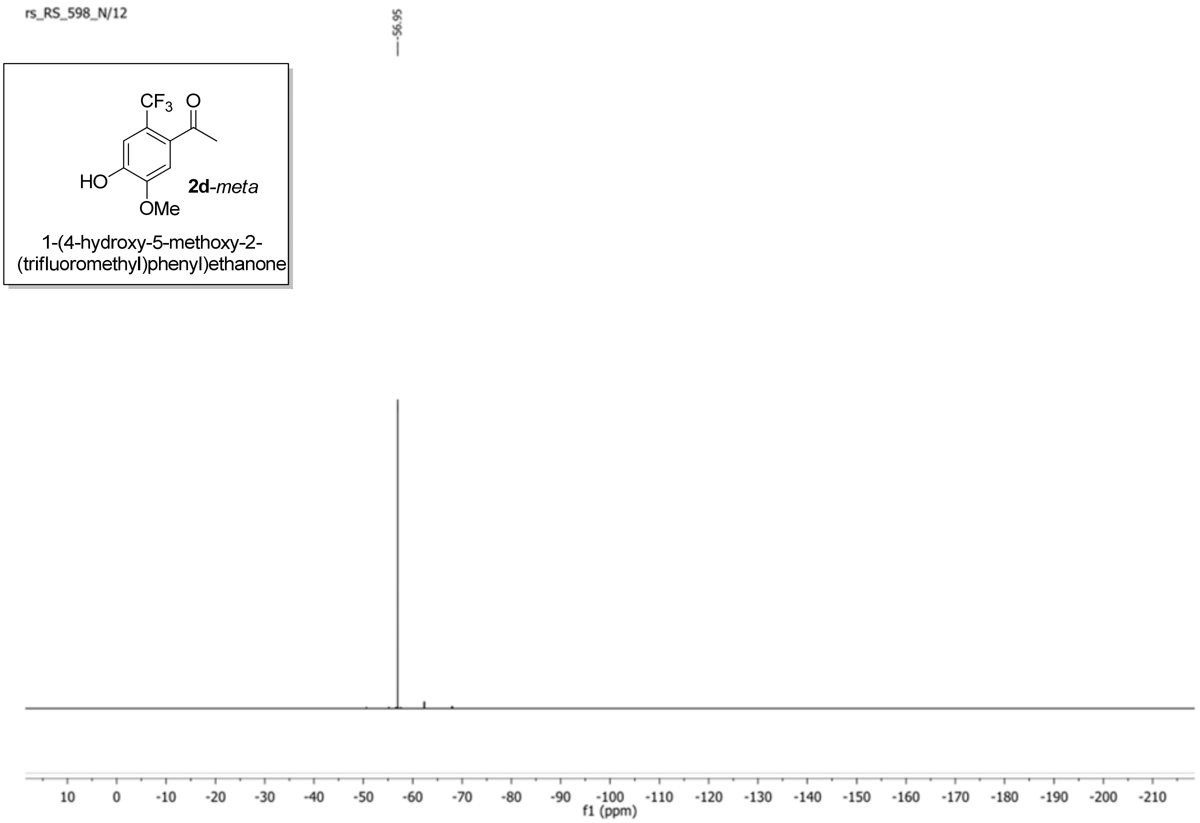

Supplementary Figure 23. ^{19}F-NMR of 1-(4-hydroxy-5-methoxy-2-(trifluoromethyl)phenyl)ethanone (**2d**-*meta*)

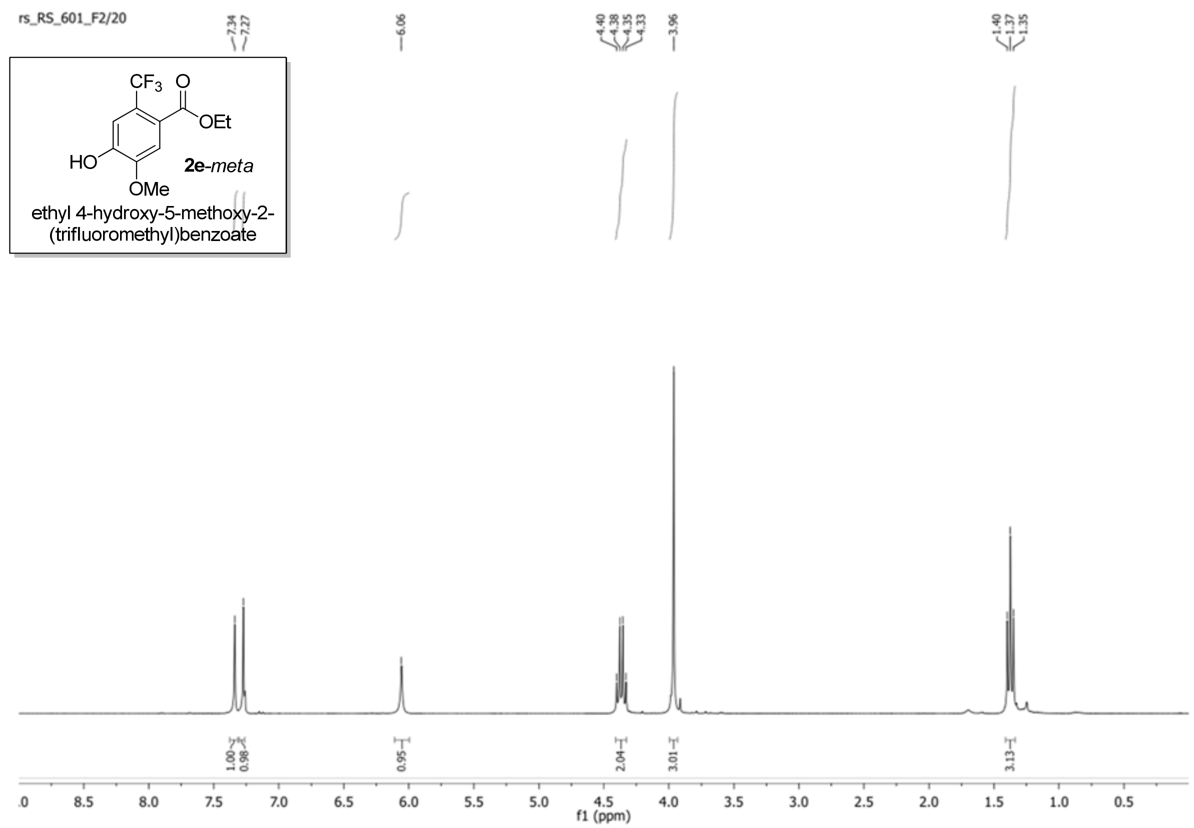

Supplementary Figure 24. ^{1}H-NMR of ethyl 4-hydroxy-5-methoxy-2-(trifluoromethyl)benzoate (**2e**-*meta*)

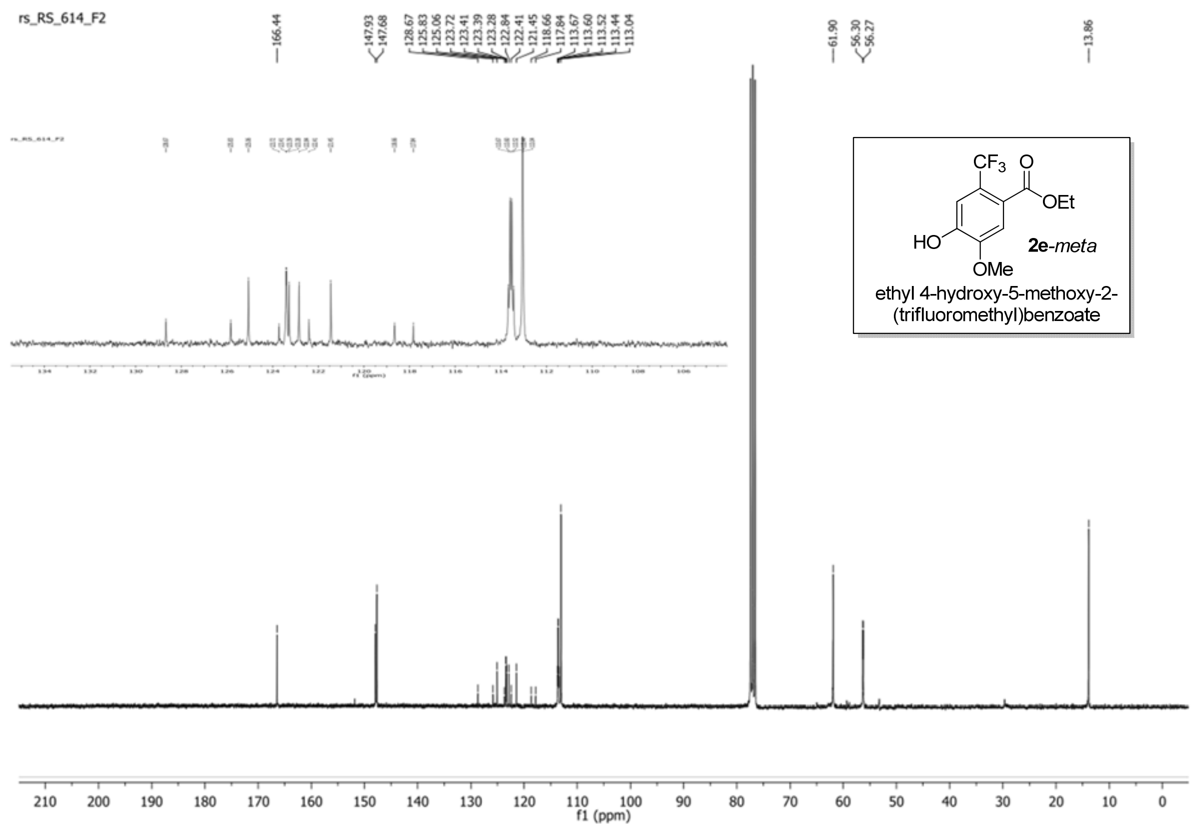

Supplementary Figure 25. ^{13}C-NMR of ethyl 4-hydroxy-5-methoxy-2-(trifluoromethyl)benzoate (**2e**-*meta*)

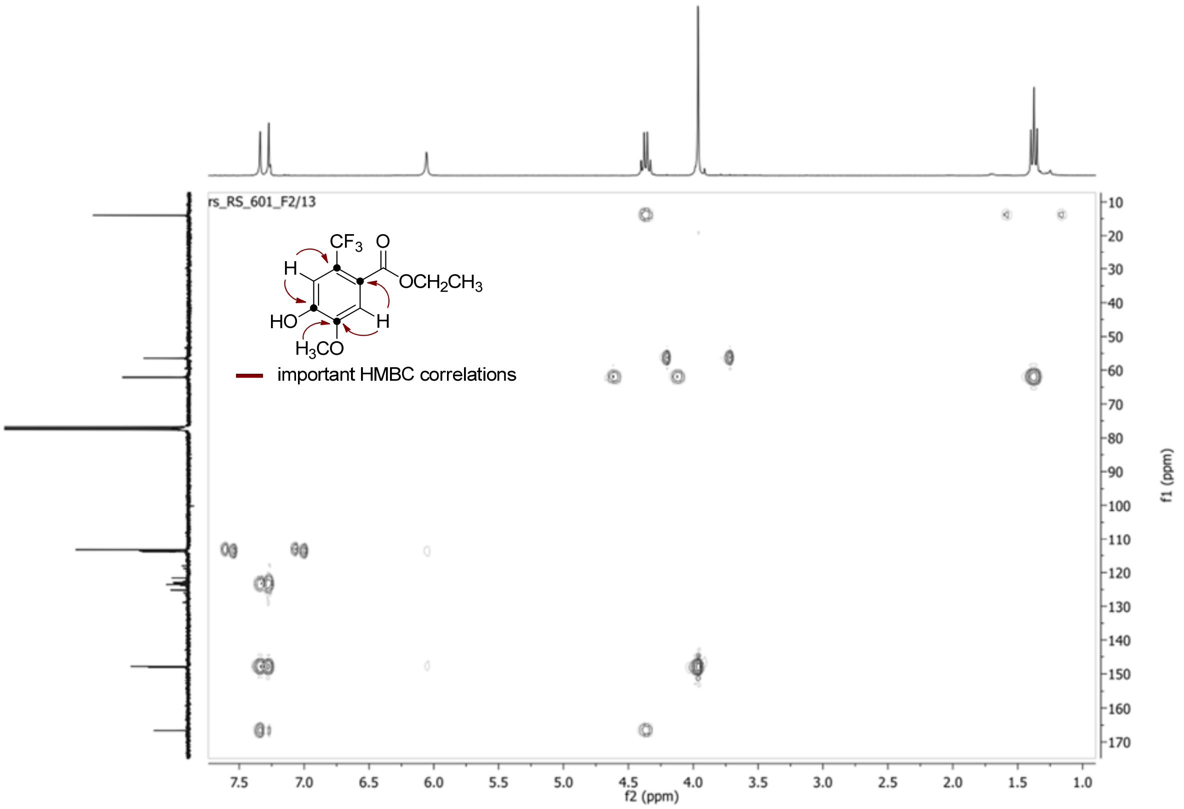

Supplementary Figure 26. HMBC of ethyl 4-hydroxy-5-methoxy-2-(trifluoromethyl)benzoate (**2e**-*meta*)

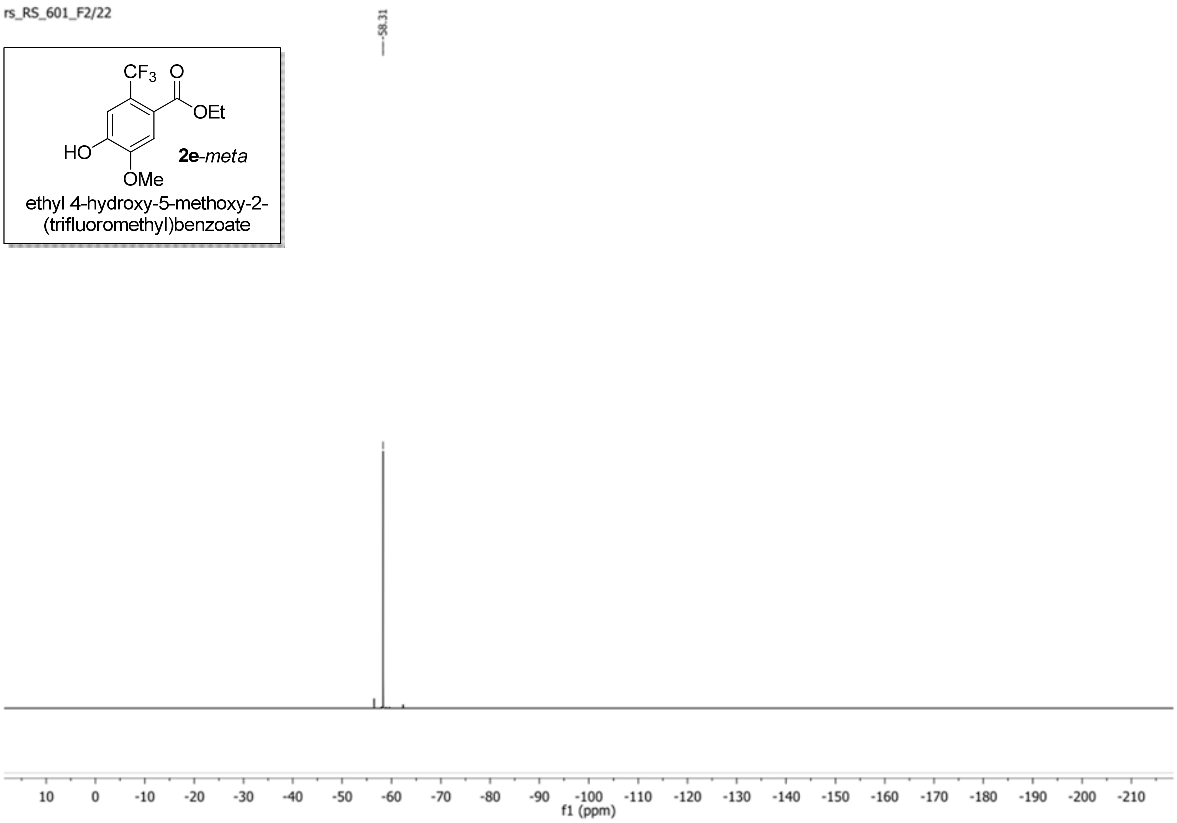

Supplementary Figure 27. ^{19}F-NMR ethyl 4-hydroxy-5-methoxy-2-(trifluoromethyl)benzoate (**2e**-*meta*)

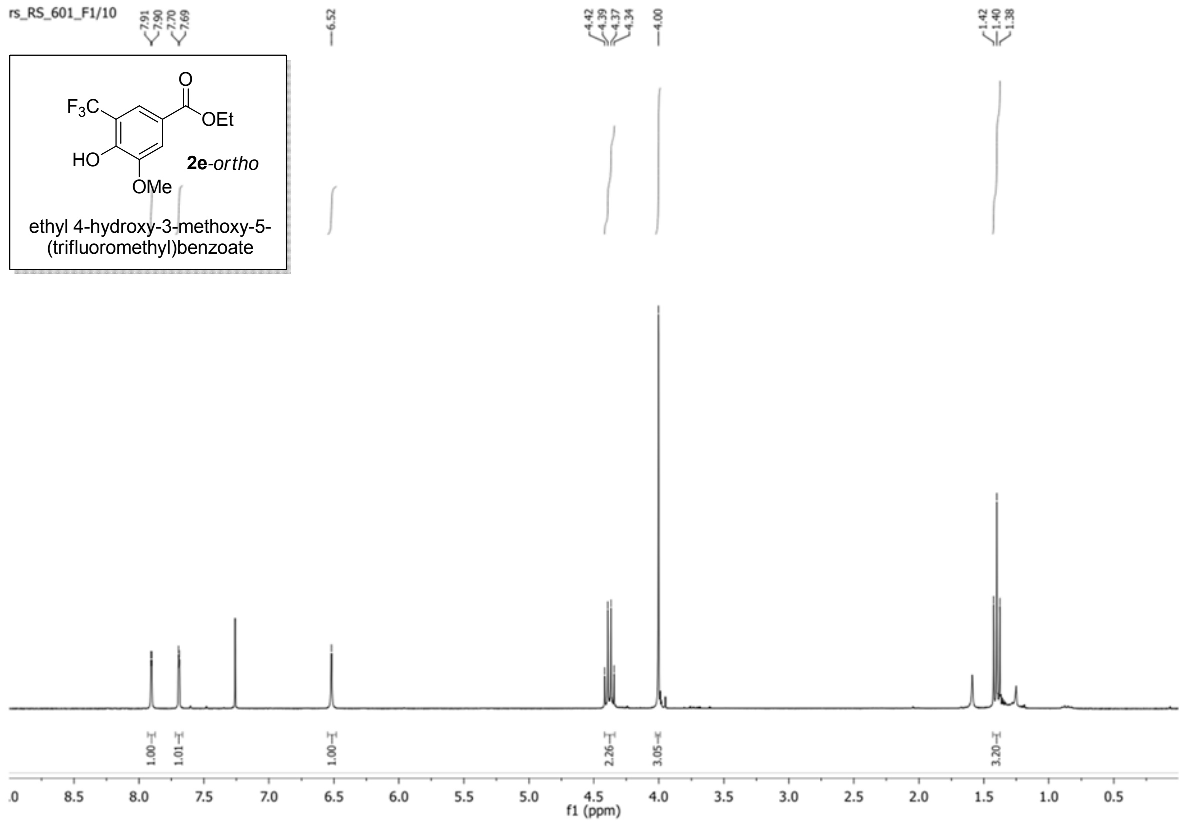

Supplementary Figure 28. ^{1}H-NMR of ethyl 4-hydroxy-3-methoxy-5-(trifluoromethyl)benzoate (**2e**-*ortho*)

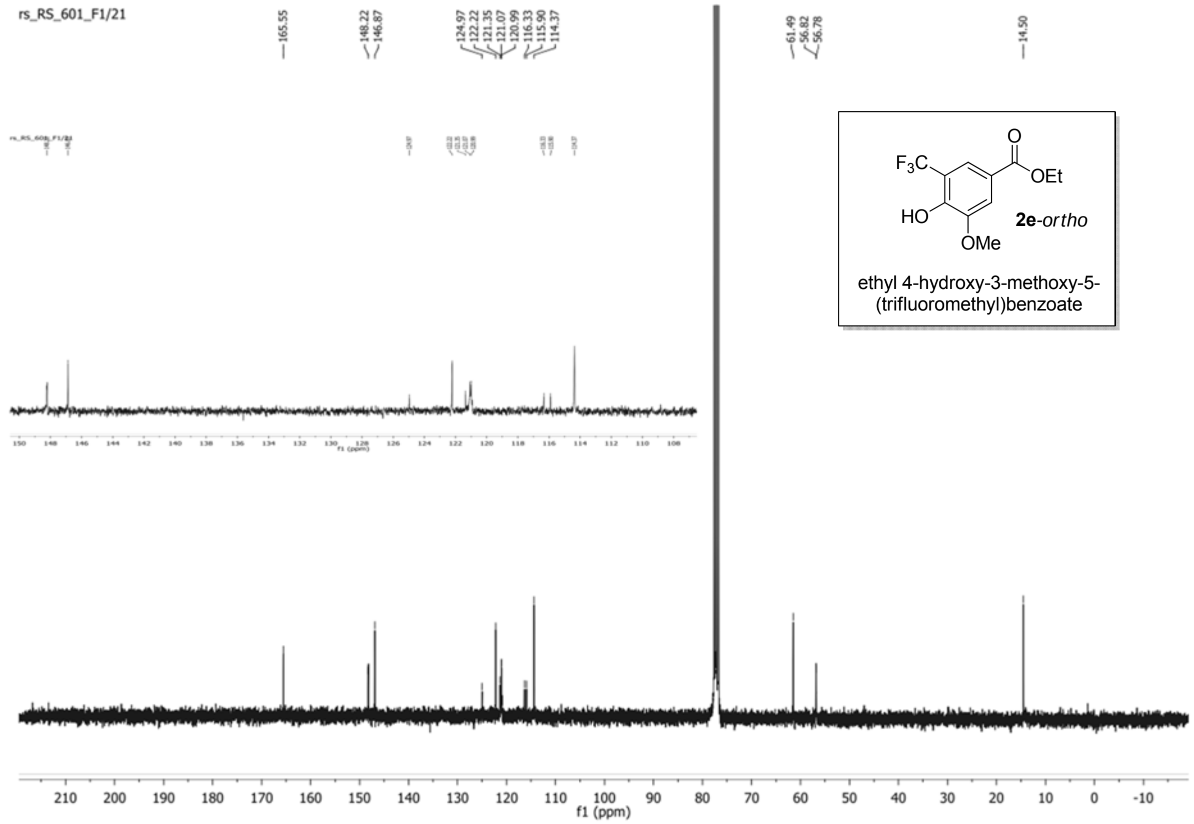

Supplementary Figure 29. ^{13}C-NMR of ethyl 4-hydroxy-3-methoxy-5-(trifluoromethyl)benzoate (**2e**-*ortho*)

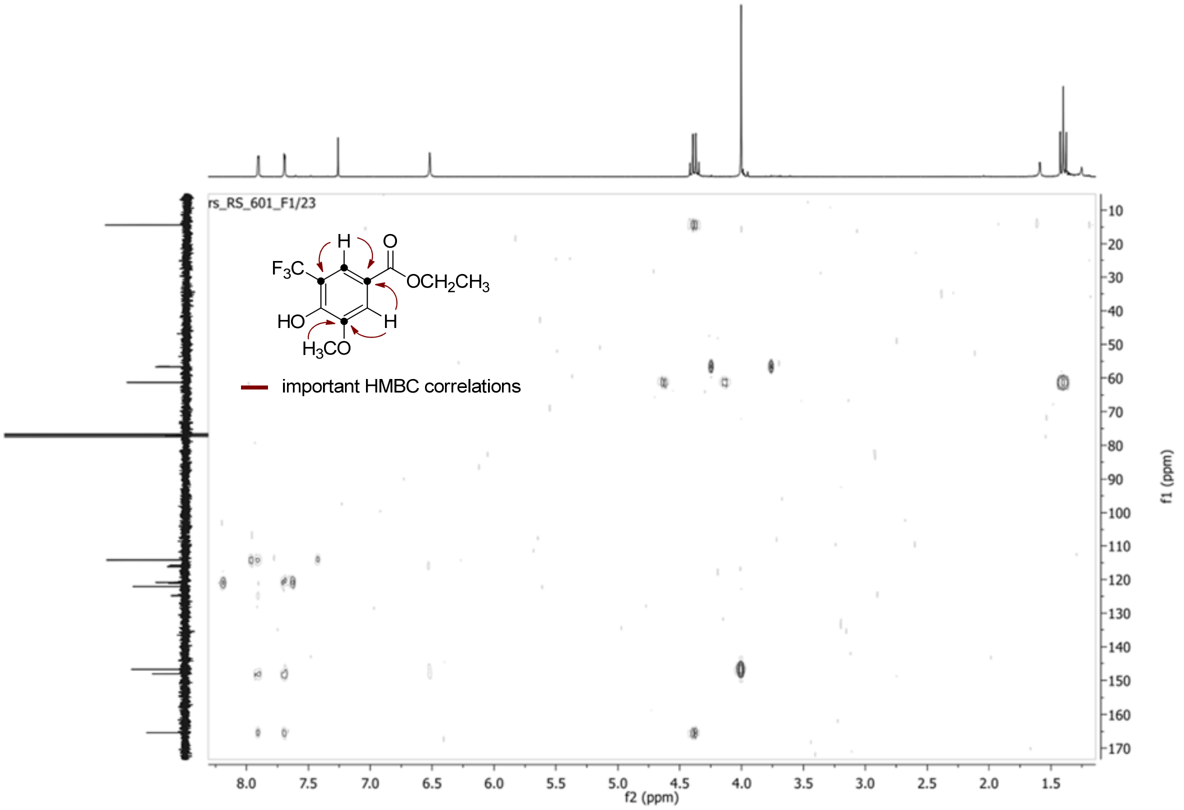

Supplementary Figure 30. HMBC of ethyl 4-hydroxy-3-methoxy-5-(trifluoromethyl)benzoate (**2e**-*ortho*)

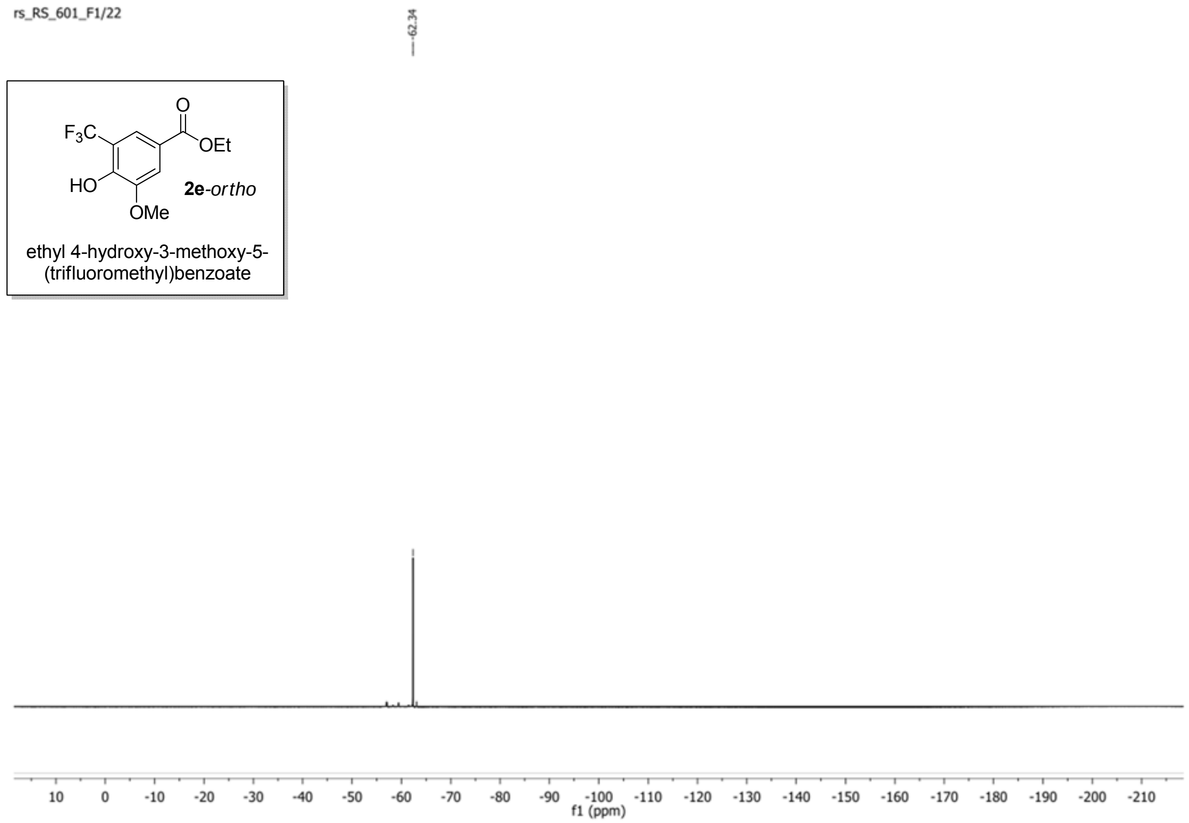

Supplementary Figure 31. ^{19}F-NMR of ethyl 4-hydroxy-3-methoxy-5-(trifluoromethyl)benzoate (**2e**-*ortho*)

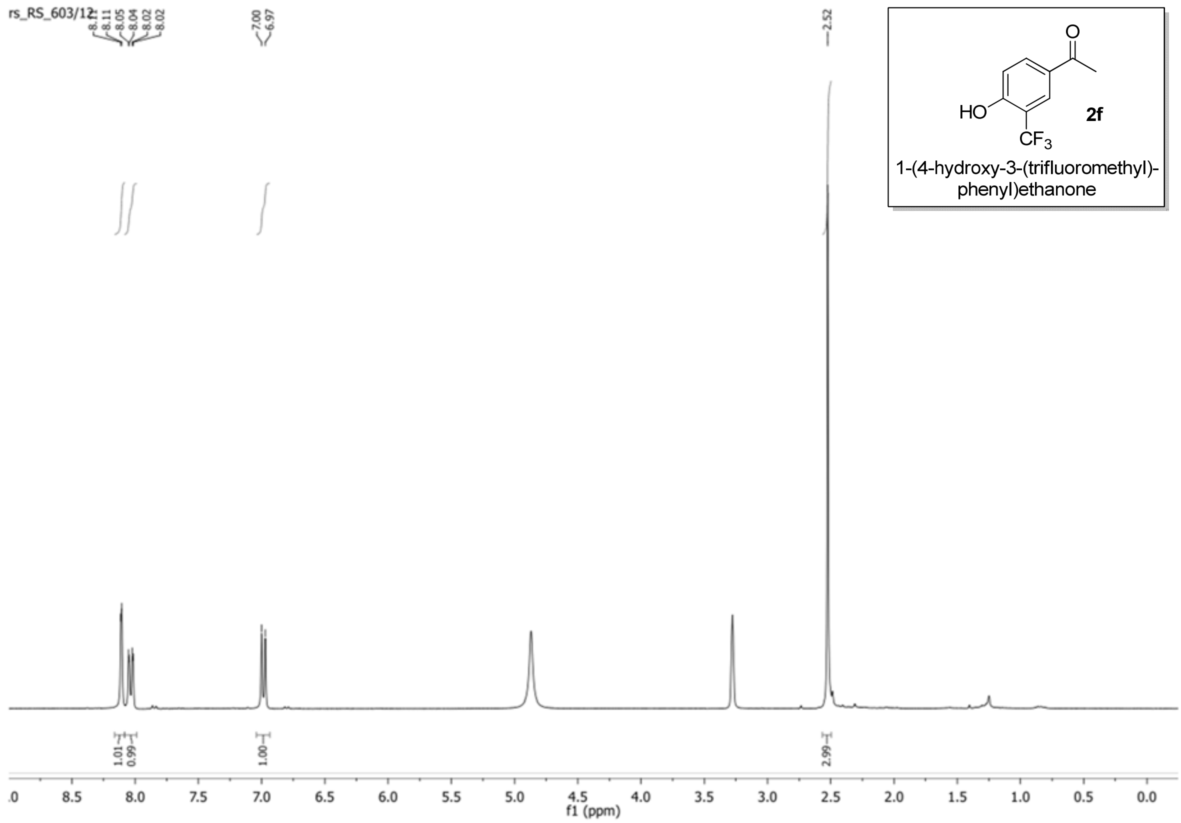

Supplementary Figure 32. ^{1}H-NMR of 1-(4-hydroxy-3-(trifluoromethyl)phenyl)ethanone (**2f**)

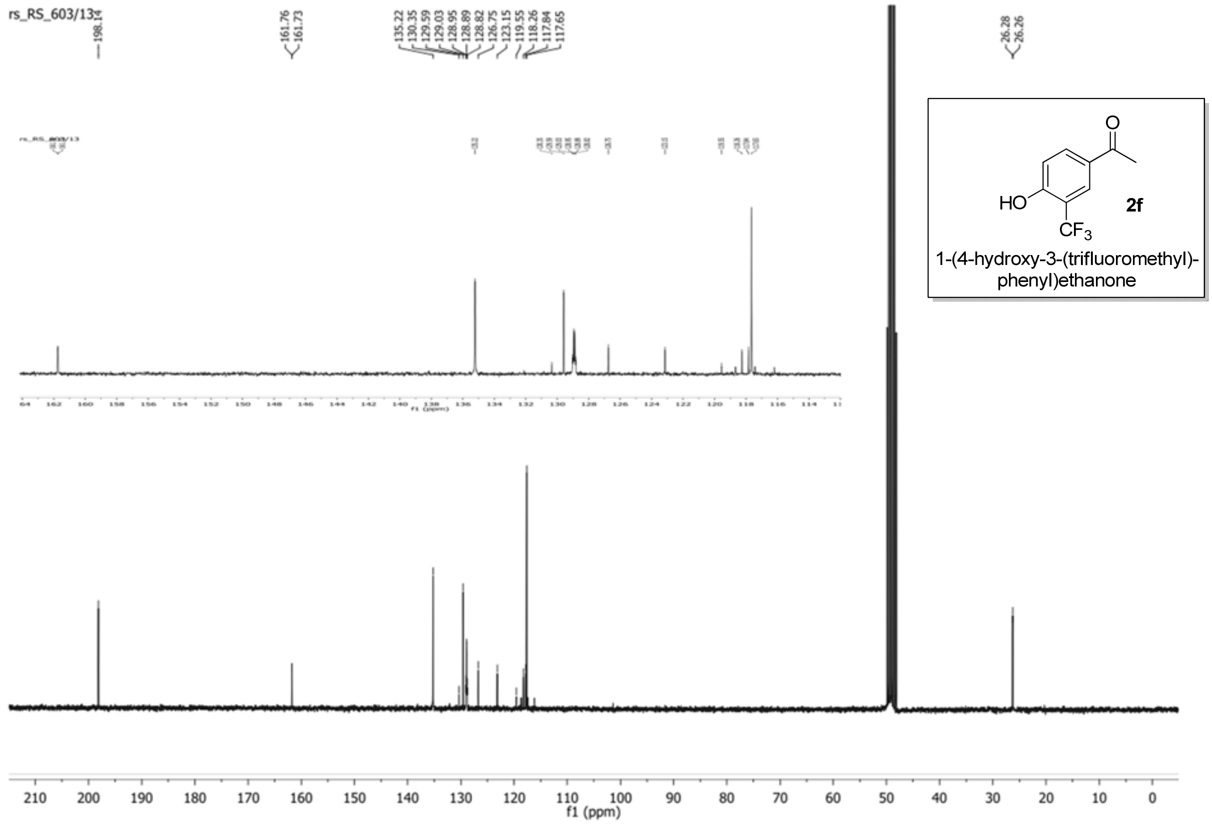

Supplementary Figure 33. ^{13}C- NMR of 1-(4-hydroxy-3-(trifluoromethyl)phenyl)ethanone (**2f**)

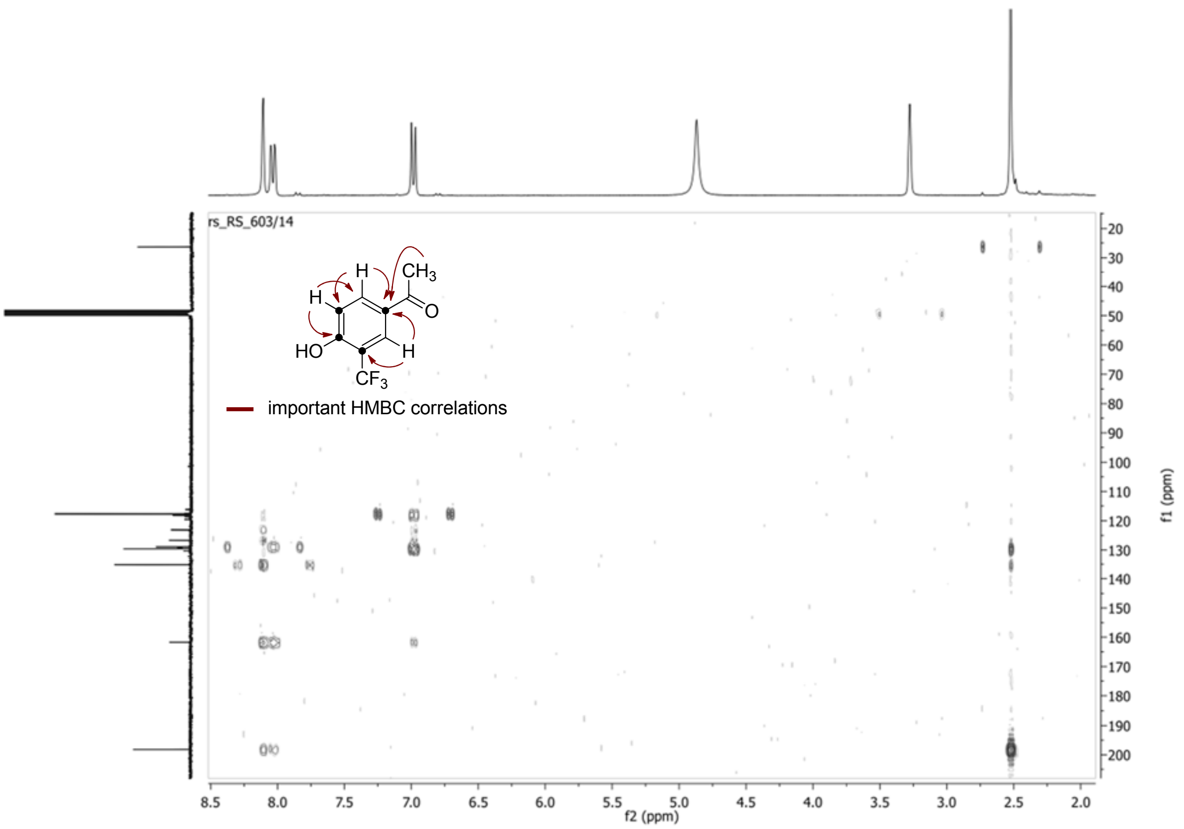

Supplementary Figure 34. HMBC of 1-(4-hydroxy-3-(trifluoromethyl)phenyl)ethanone (**2f**)

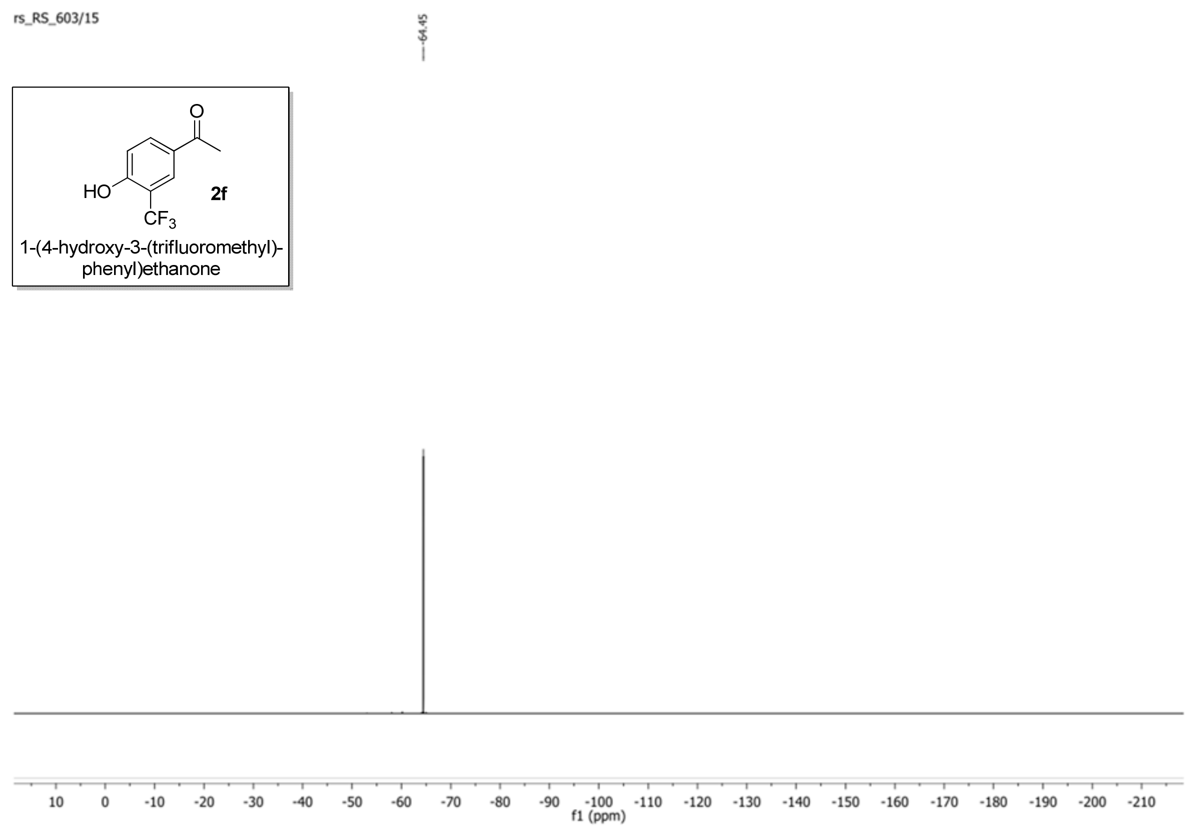

Supplementary Figure 35. ^{19}F-NMR 1-(4-hydroxy-3-(trifluoromethyl)phenyl)ethanone (**2f**)

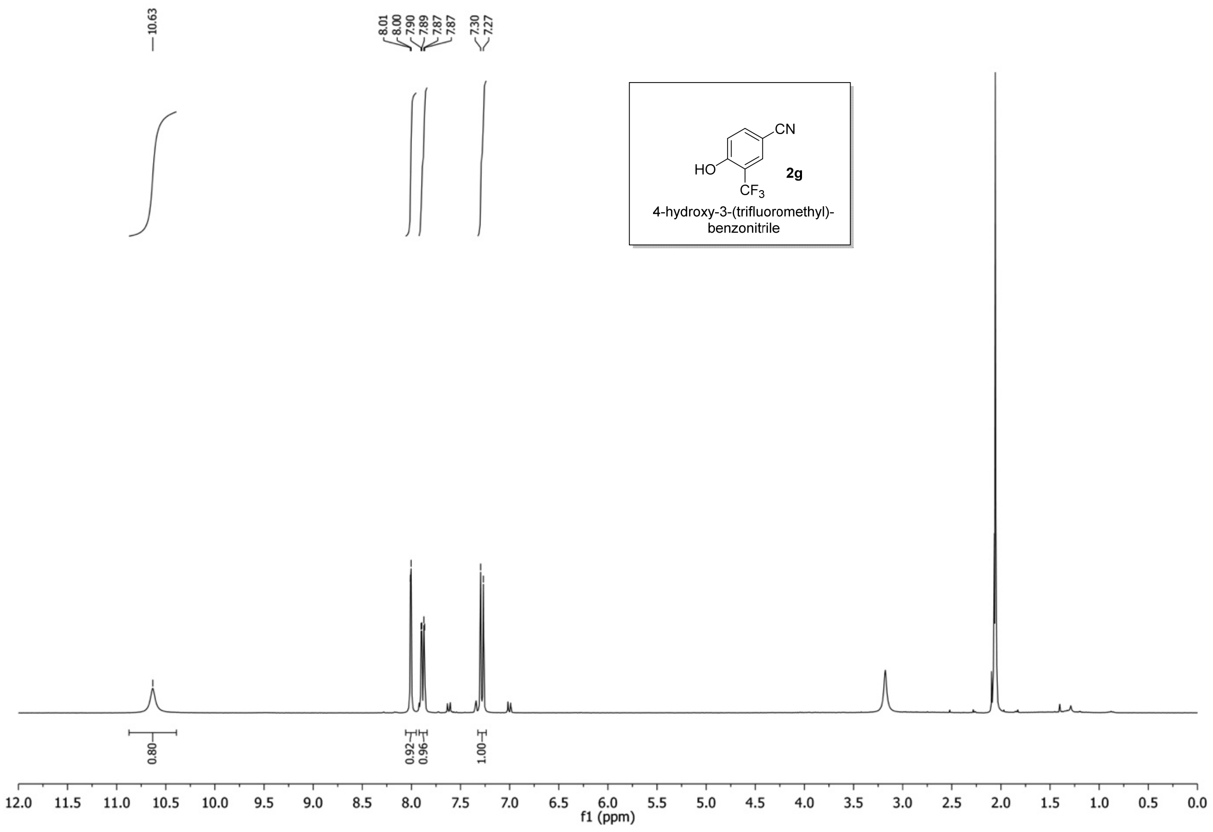

Supplementary Figure 36. ^{1}H-NMR of 4-hydroxy-3-(trifluoromethyl)benzonitrile (**2g**)

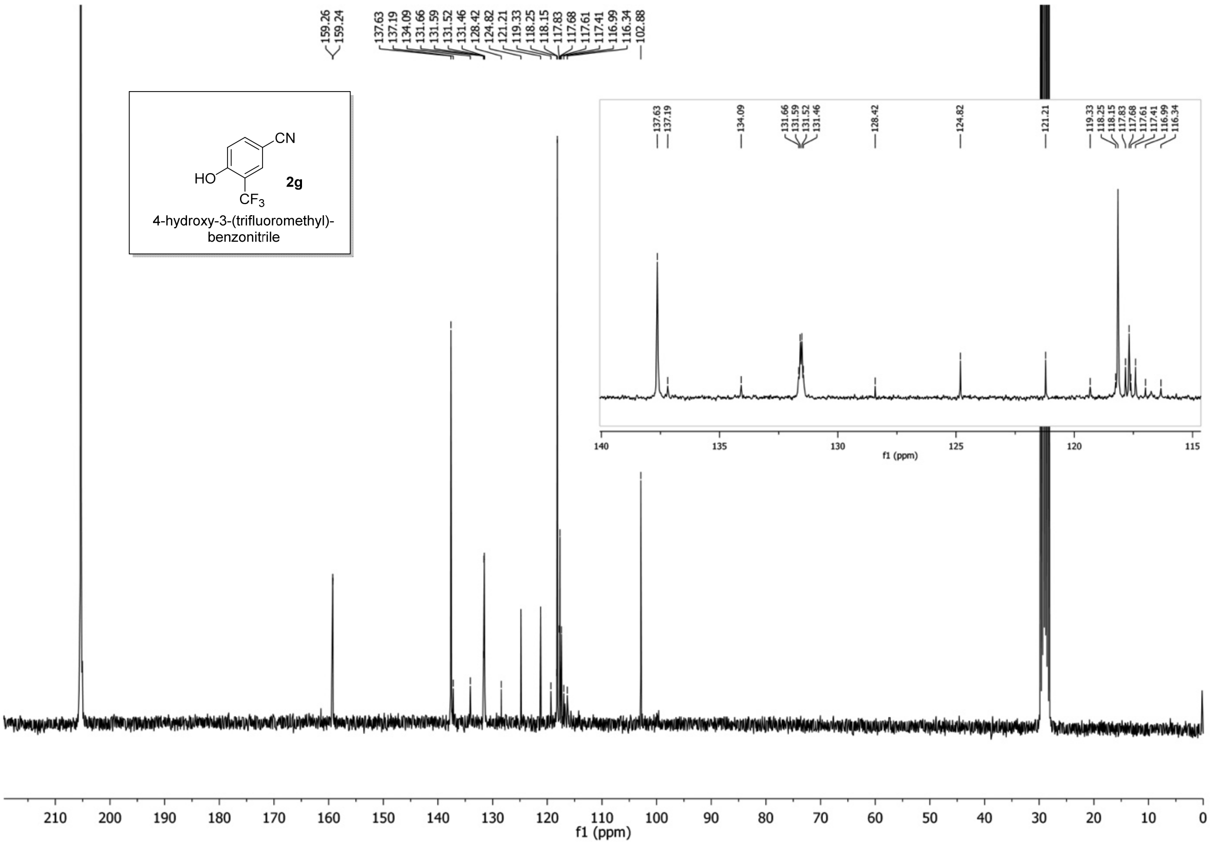

Supplementary Figure 37. ^{13}C-NMR of 4-hydroxy-3-(trifluoromethyl)benzonitrile (**2g**)

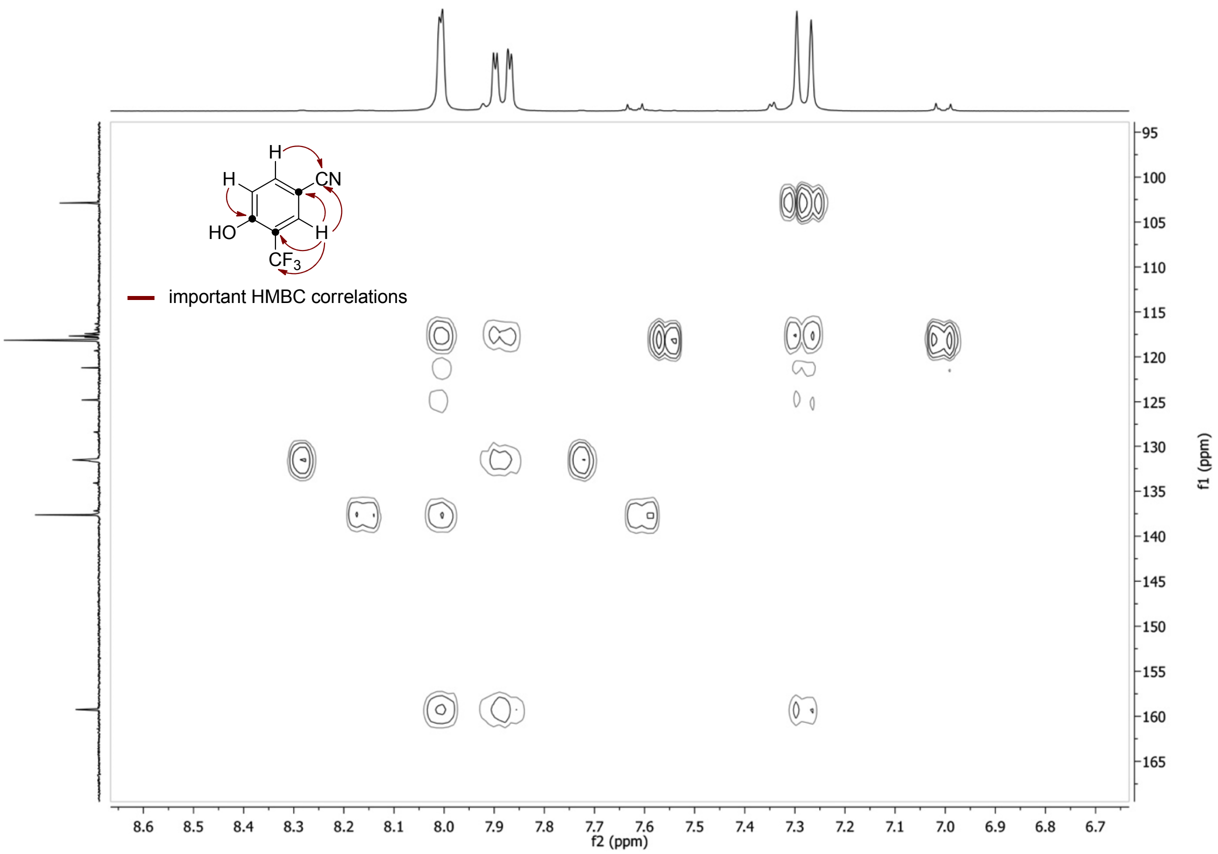

Supplementary Figure 38. HMBC NMR of 4-hydroxy-3-(trifluoromethyl)benzonitrile (**2g**)

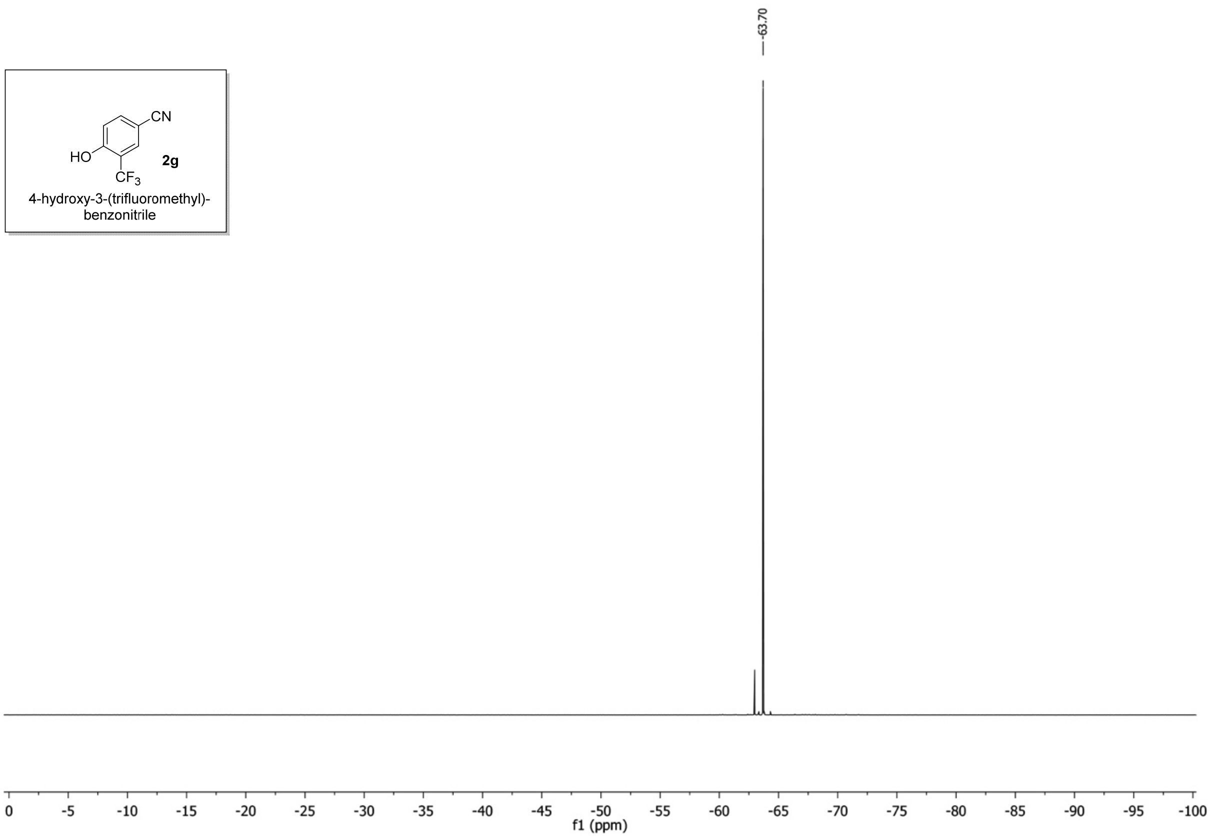

Supplementary Figure 39. ^{19}F-NMR of 4-hydroxy-3-(trifluoromethyl)benzonitrile (**2g**)

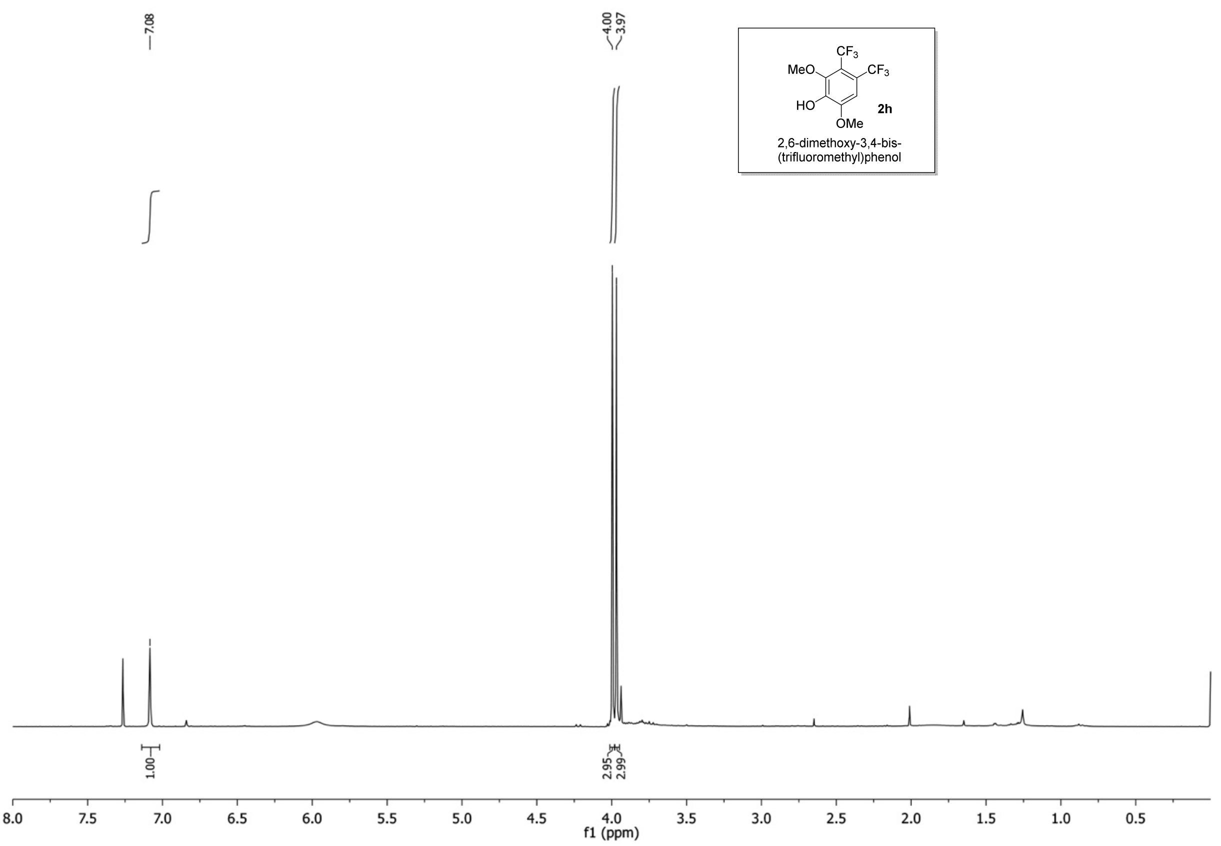

Supplementary Figure 40. ^{1}H-NMR of 2,6-dimethoxy-3,4-bis(trifluoromethyl)phenol (**2h**)

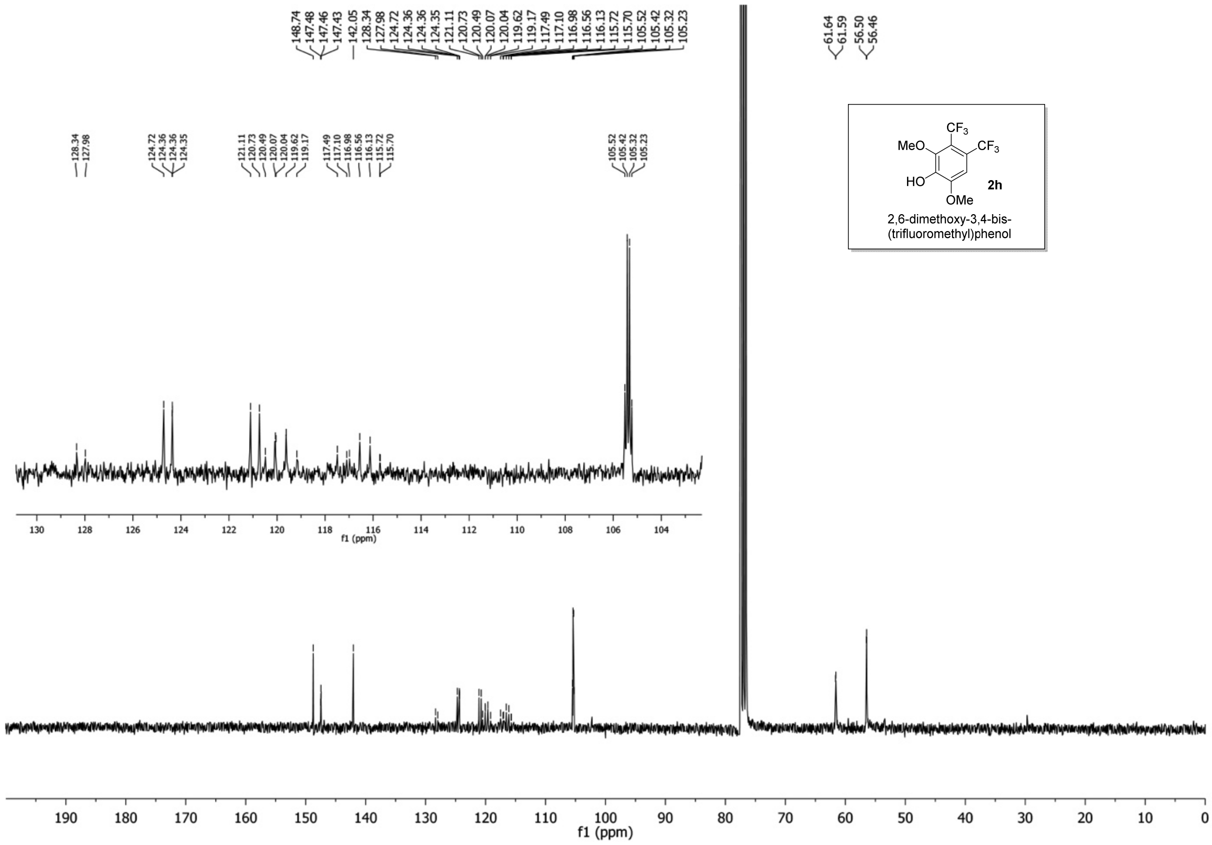

Supplementary Figure 41. ^{13}C-NMR of 2,6-dimethoxy-3,4-bis(trifluoromethyl)phenol (**2h**)

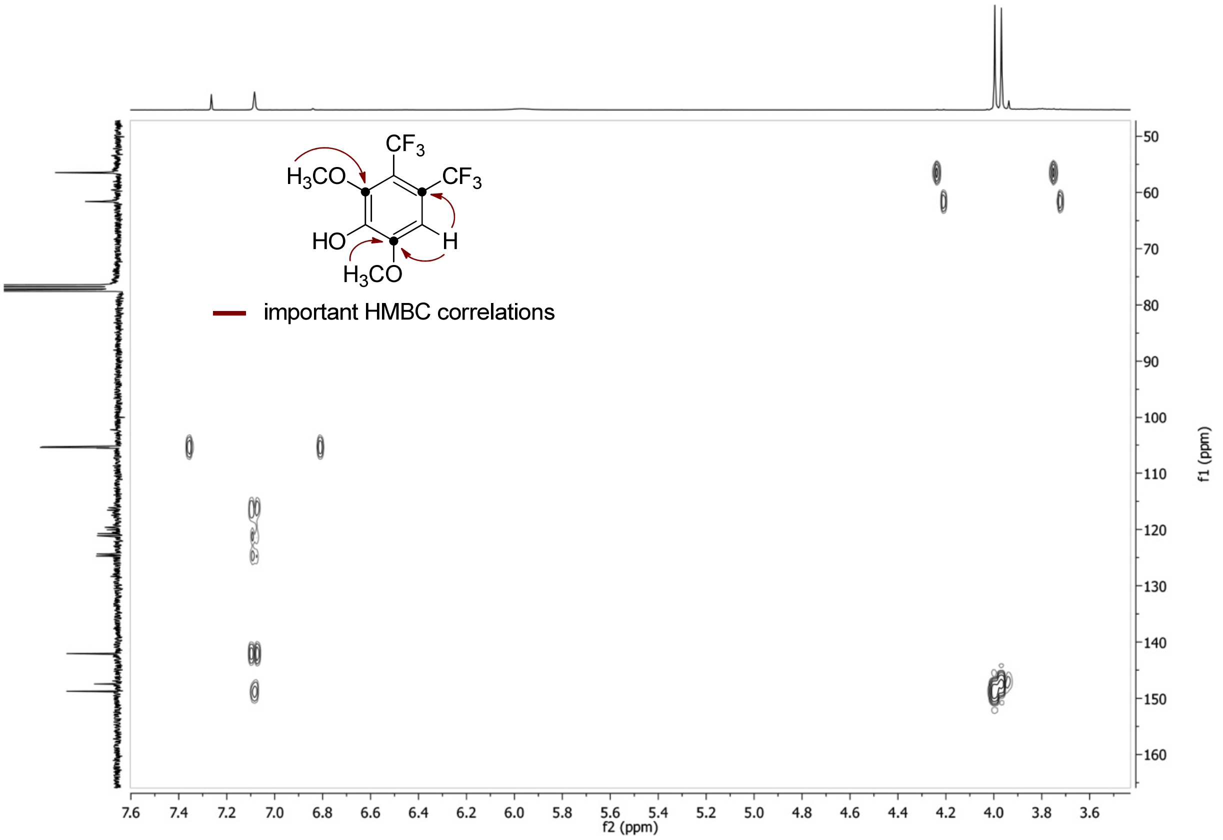

Supplementary Figure 42. HMBC NMR of 2,6-dimethoxy-3,4-bis(trifluoromethyl)phenol (**2h**)

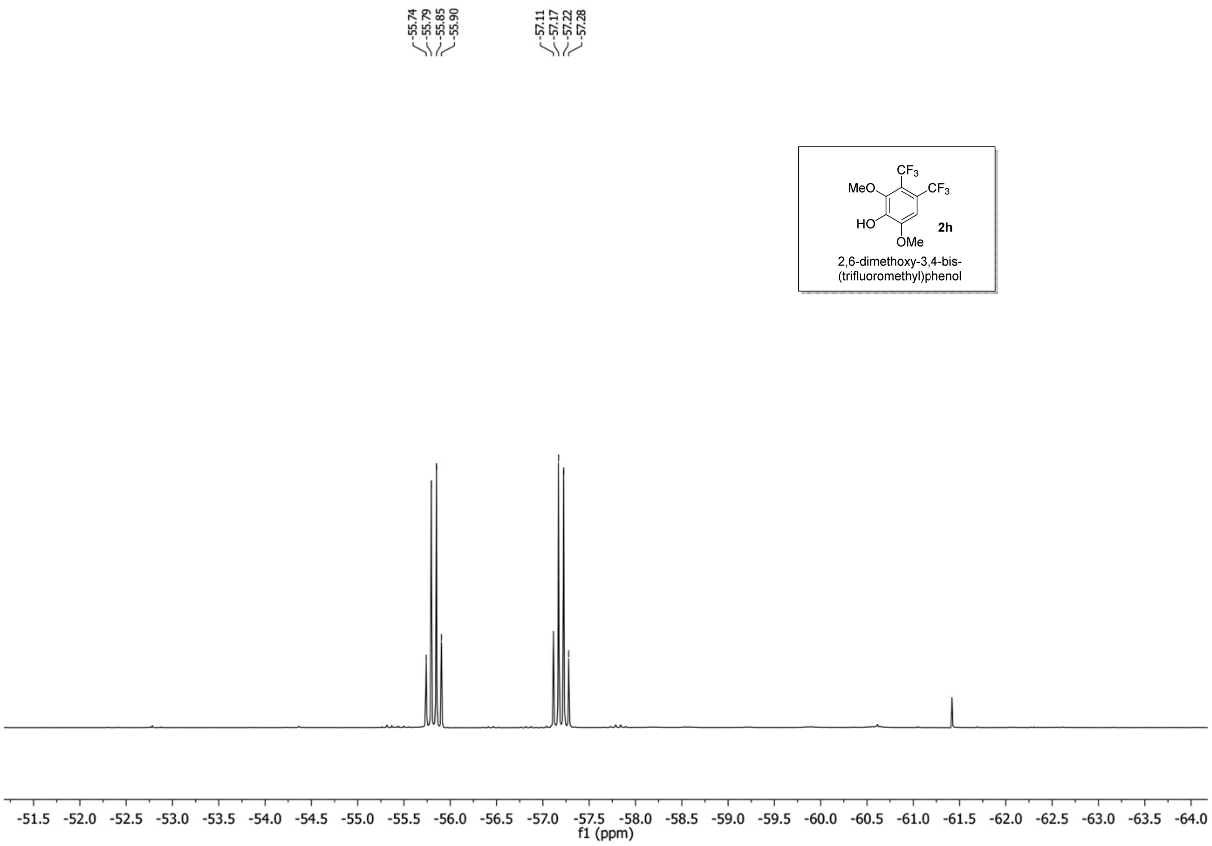

Supplementary Figure 43. ^{19}F-NMR of 2,6-dimethoxy-3,4-bis(trifluoromethyl)phenol (**2h**)

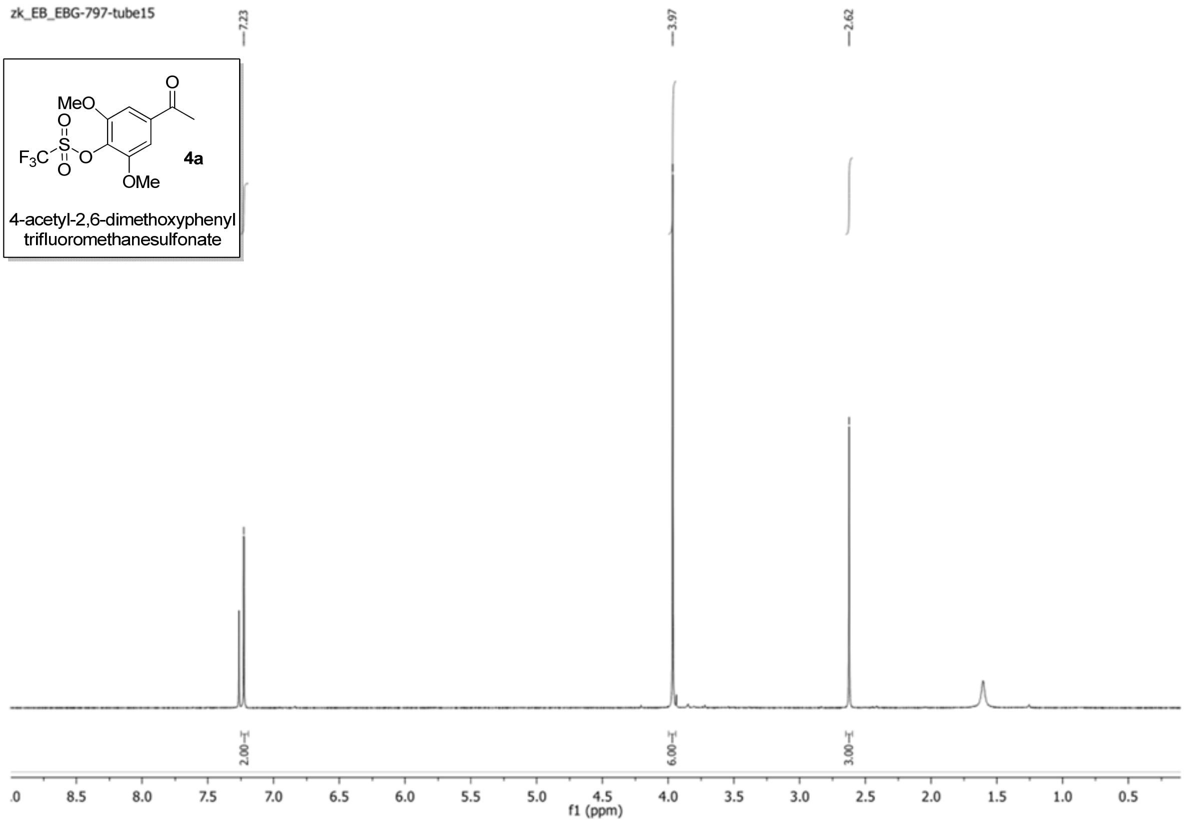

Supplementary Figure 44. ^{1}H-NMR of 4-acetyl-2,6-dimethoxyphenyl trifluoromethanesulfonate (**4a**)

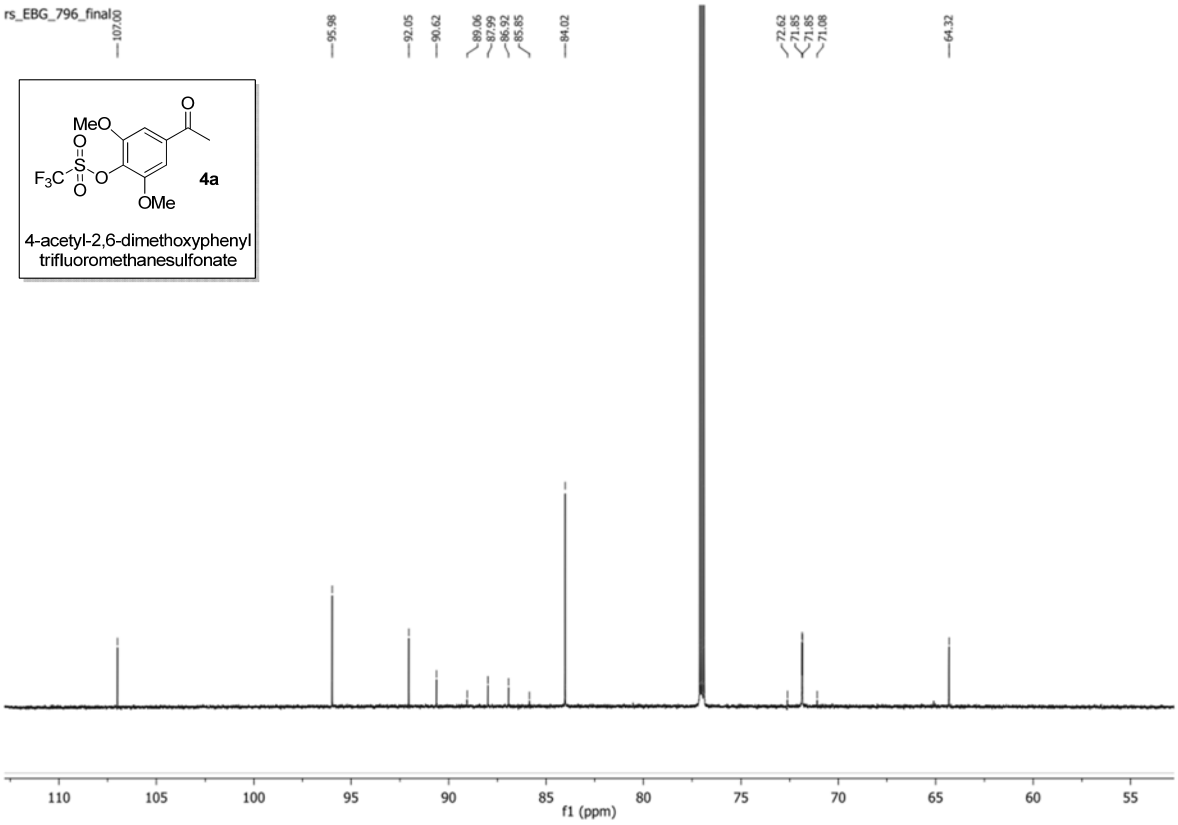

Supplementary Figure 45. ^{13}C-NMR of 4-acetyl-2,6-dimethoxyphenyl trifluoromethanesulfonate (**3a**)

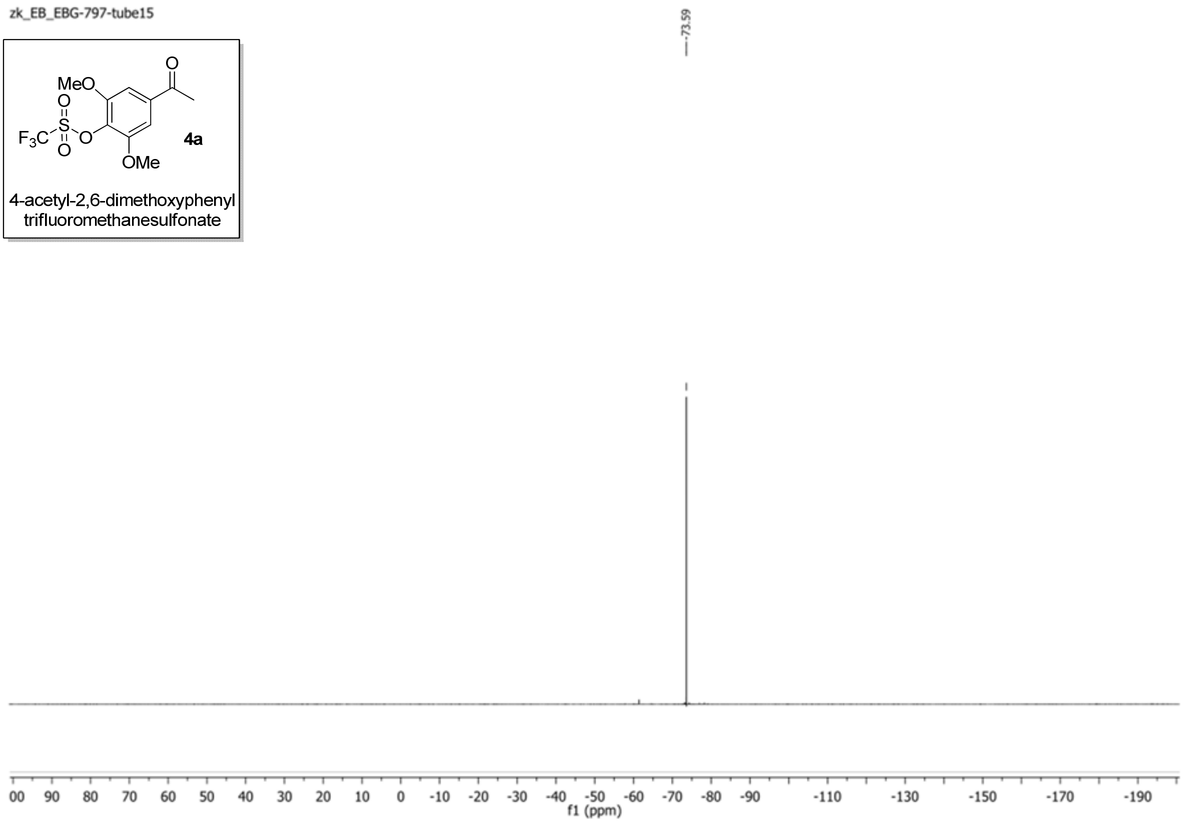

Supplementary Figure 46. ^{19}F-NMR of 4-acetyl-2,6-dimethoxyphenyl trifluoromethanesulfonate (**3a**)

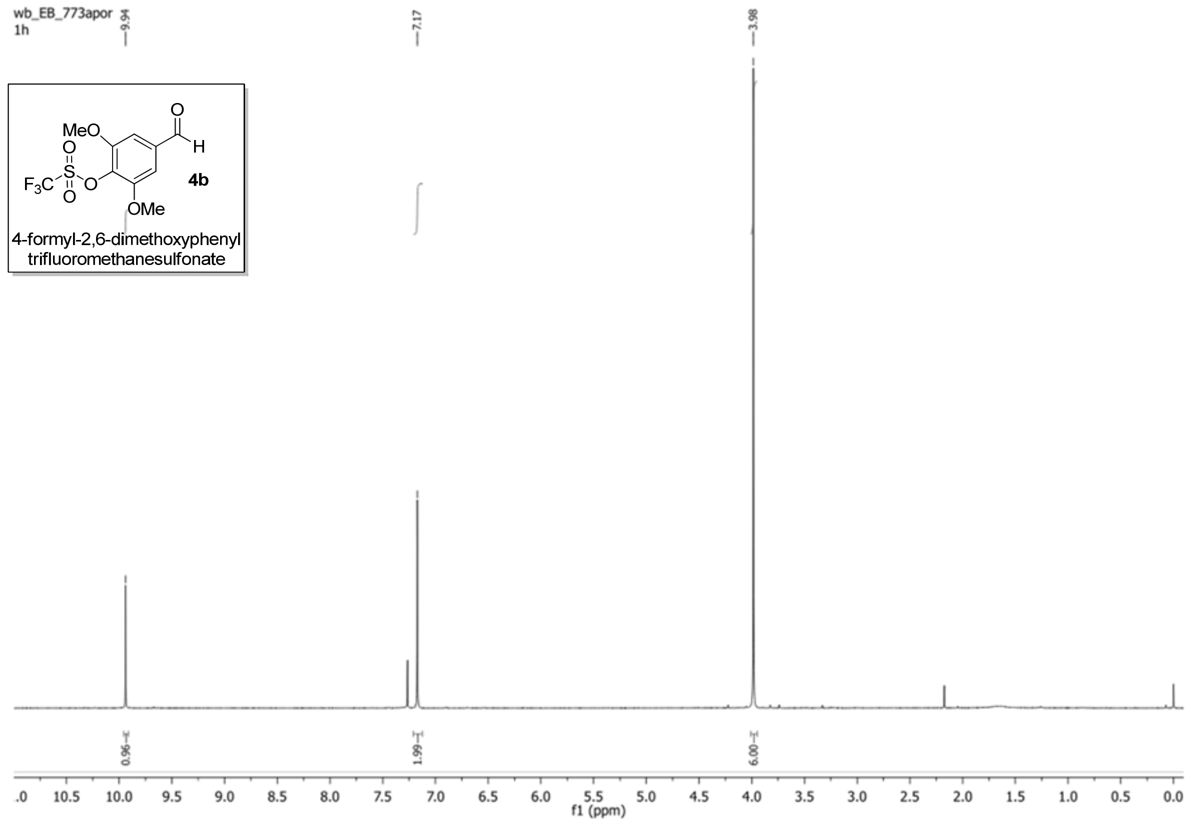

Supplementary Figure 47. ^{1}H-NMR of 4-formyl-2,6-dimethoxyphenyl trifluoromethanesulfonate (**4b**)

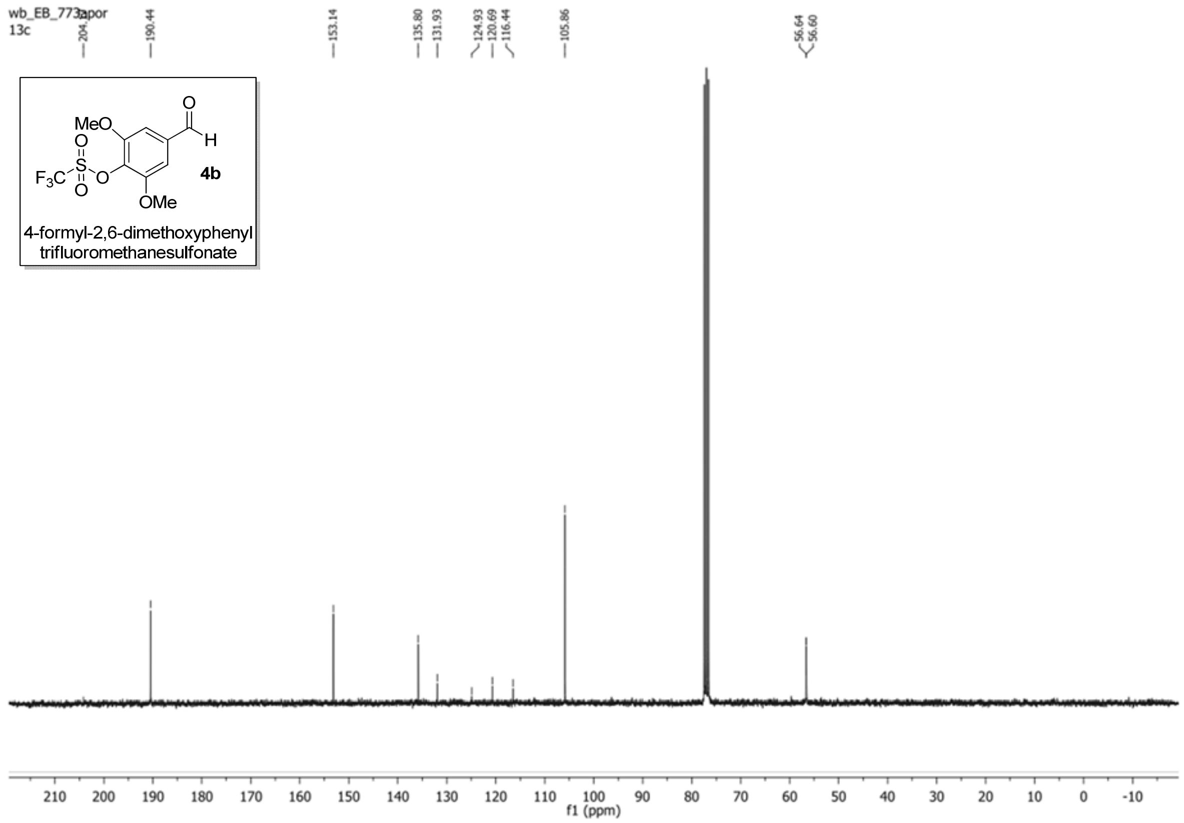

Supplementary Figure 48. ^{13}C-NMR of 4-formyl-2,6-dimethoxyphenyl trifluoromethanesulfonate (**3b**)

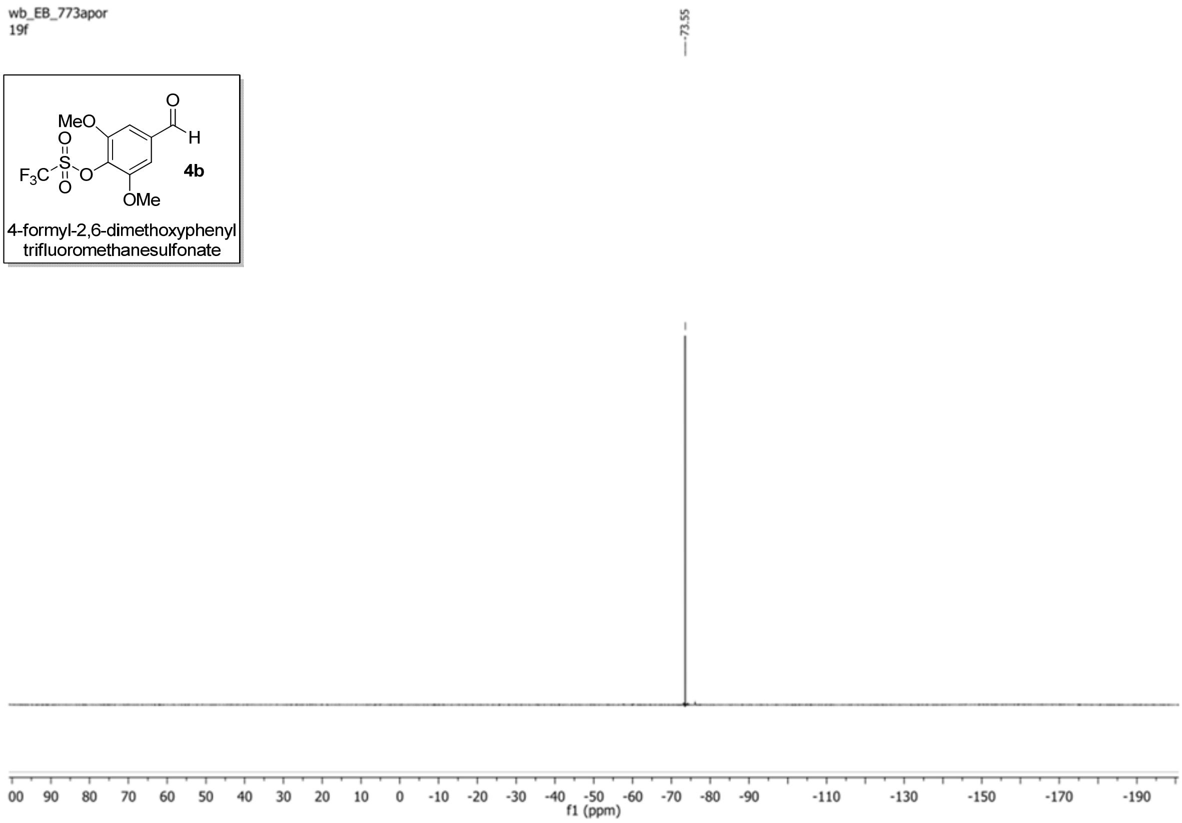

Supplementary Figure 49. ^{19}F-NMR of 4-formyl-2,6-dimethoxyphenyl trifluoromethanesulfonate (**3b**)

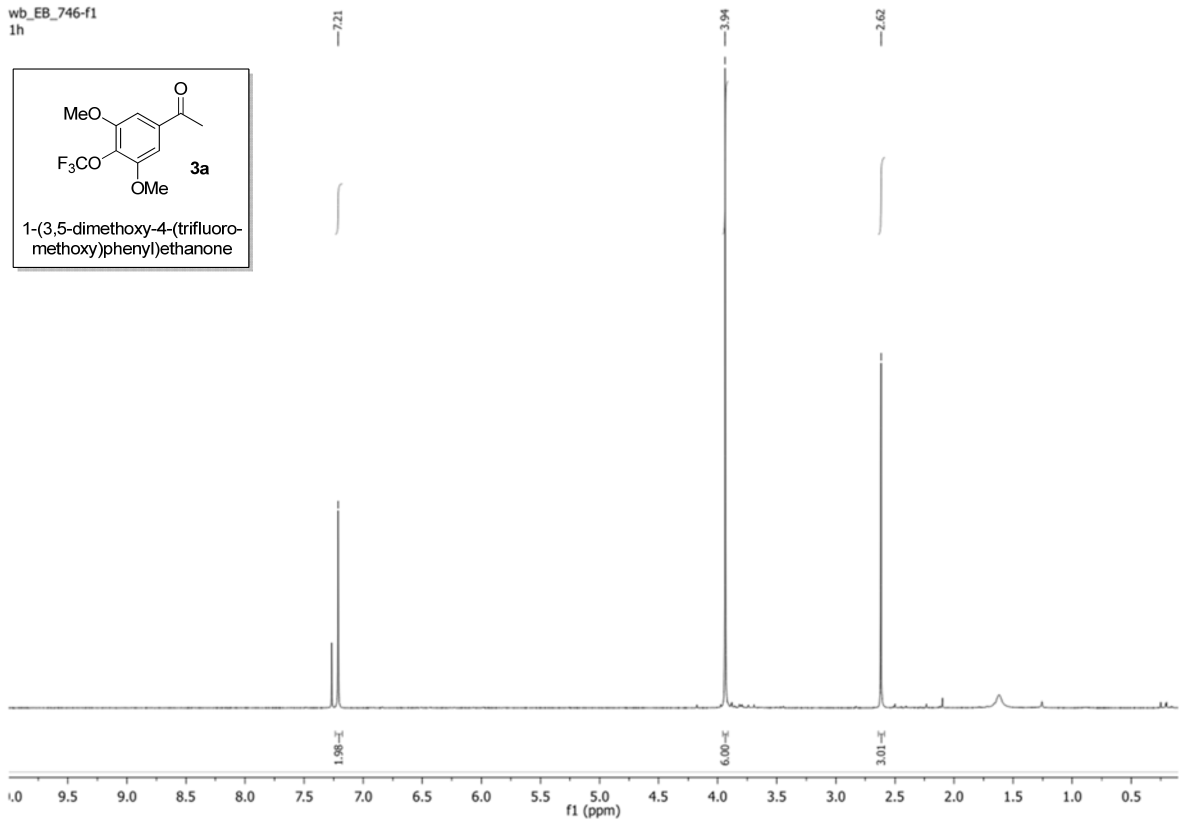

Supplementary Figure 50. ^{1}H-NMR of 1-(3,5-dimethoxy-4-(trifluoromethoxy)phenyl)ethanone (**3a**)

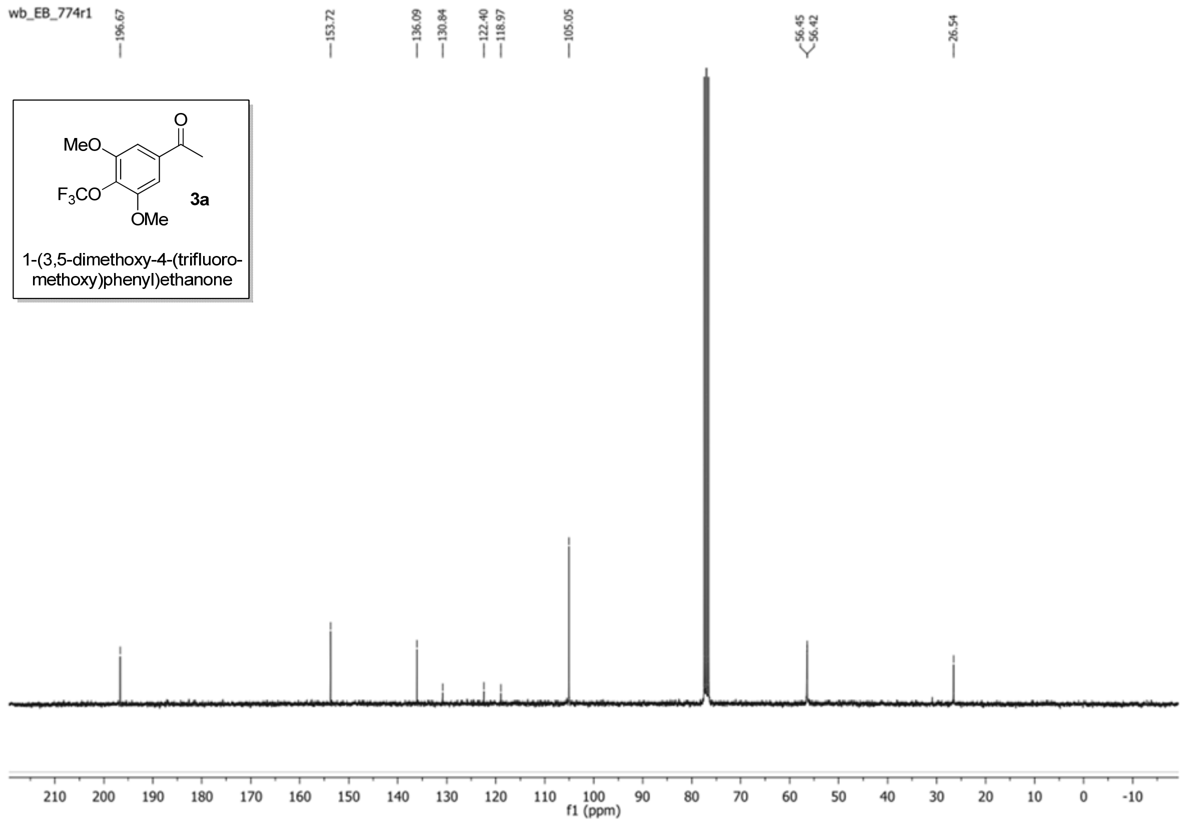

Supplementary Figure 51. ^{13}C-NMR of 1-(3,5-dimethoxy-4-(trifluoromethoxy)phenyl)ethanone (**3a**)

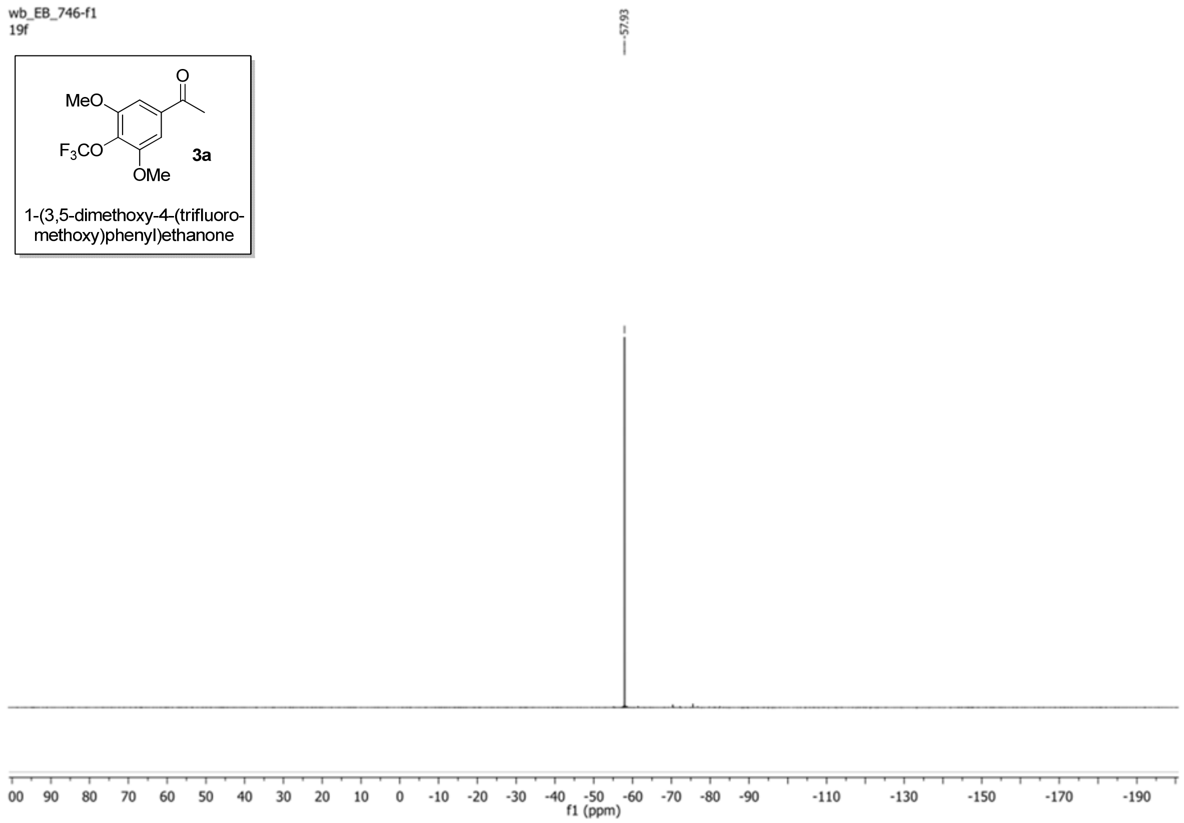

Supplementary Figure 52. ^{19}F-NMR of 1-(3,5-dimethoxy-4-(trifluoromethoxy)phenyl)ethanone (**3a**)

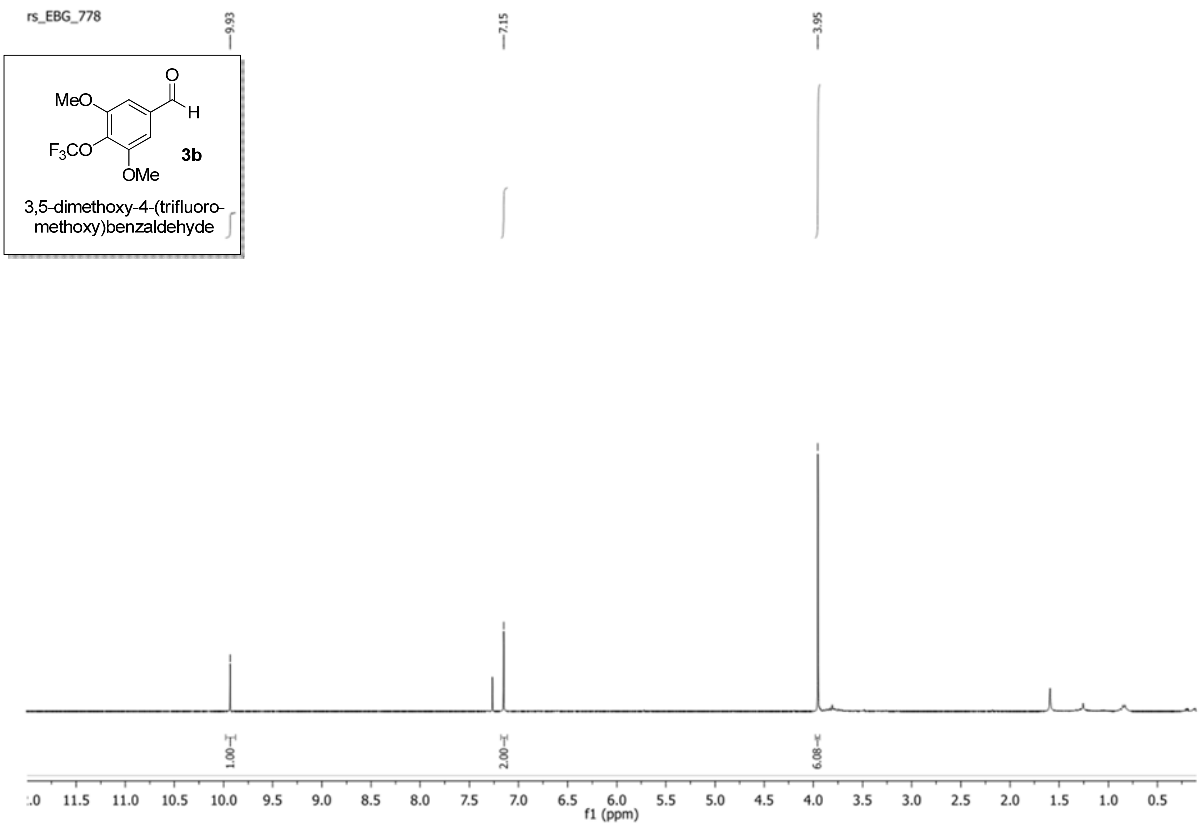

Supplementary Figure 53. ^{1}H-NMR of 3,5-dimethoxy-4-(trifluoromethoxy)benzaldehyde **(3b)**

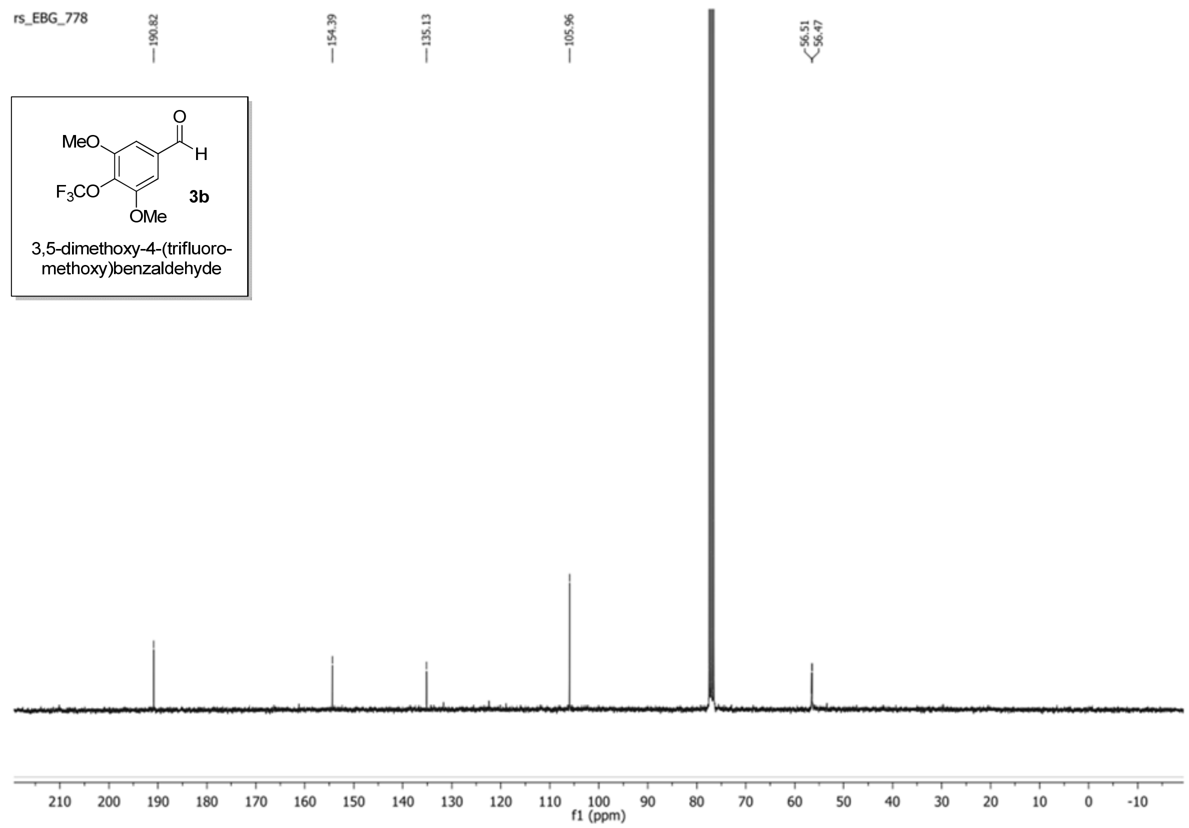

Supplementary Figure 54. ^{13}C-NMR of 3,5-dimethoxy-4-(trifluoromethoxy)benzaldehyde (**3b**)

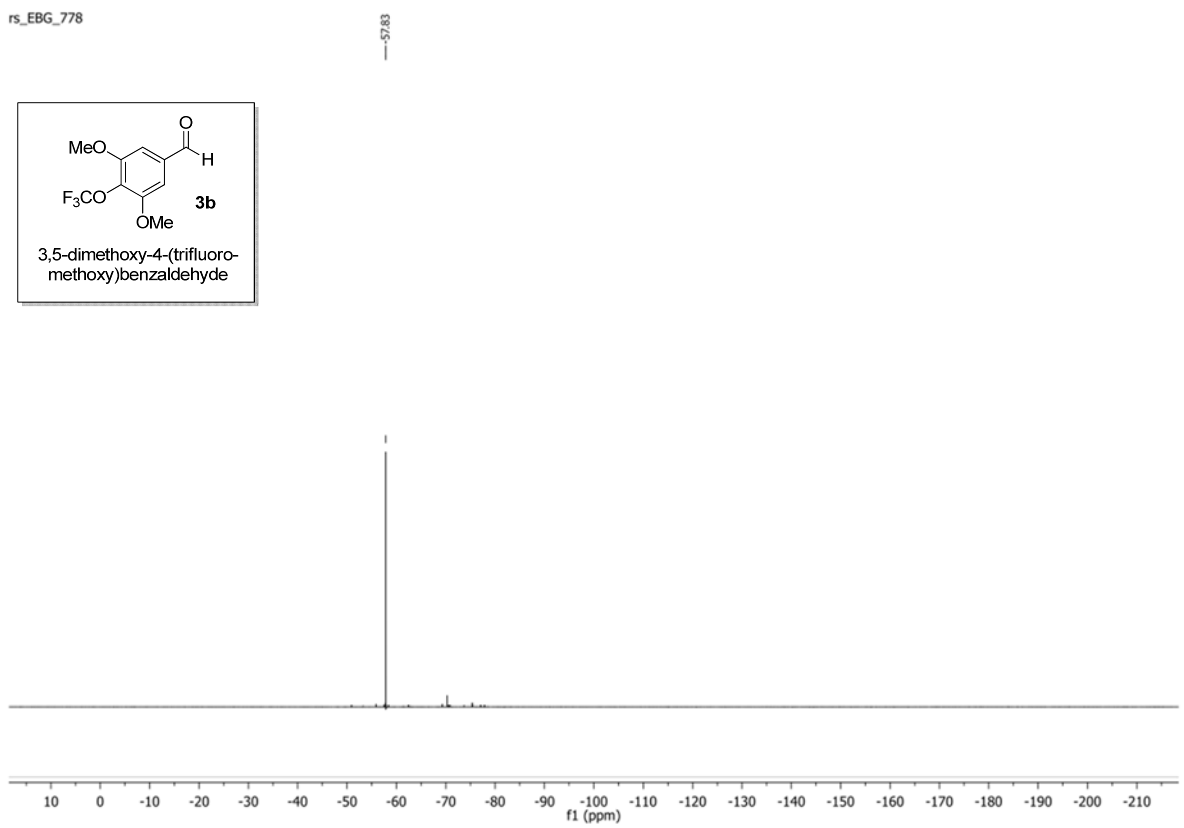

Supplementary Figure 55. ^{19}F-NMR of 3,5-dimethoxy-4-(trifluoromethoxy)benzaldehyde (**3b**)

Supplementary Table 1: Reagents and conditions: sodium acetate buffer (250 mM, pH 5.5), substrate **1a** = 50 mM (4.91 mg per 500 µL), $Zn(SO_2CF_3)_2$ = 1.0-2.5 eq. (8.29 mg, 12.43 mg, 16.6 mg, 20.7 mg per 500 µL), *t*BuOOH 4-8 eq. (aq. 70 wt% solution: 13.7 µL, 20.6 µL, 27.5 µL per 500 µL); Laccase (2.5 mg per 500 µL), reaction time 24 hours, 30 °C, 900 rpm in Eppendorf orbital shaker (horizontal position), with syringe needle for air exchange in the top lid of the Eppendorf tube (2.0 mL).

entry	eq. $Zn(SO_2CF_3)_2$	eq. *t*BuOOH	overall conv. [%]	conv. **2a** [%]
1	1.0	4	32.4 ± 1.0	0.8 ± 0.1
2	1.0	6	47.4 ± 4.2	1.2 ± 0.1
3	1.0	8	56.2 ± 5.4	1.6 ± 0.1
4	1.5	4	85.6 ± 0.4	44.9 ± 1.0
5	1.5	6	88.4 ± 4.8	38.6 ± 3.9
6	1.5	8	92.6 ± 2.0	45.0 ± 7.2
7	2.0	4	95.8 ± 0.4	55.1 ± 0.7
8	2.0	6	99.2 ± 0.2	57.5 ± 1.0
9	2.0	8	99.4 ± 0.2	58.0 ± 1.7
10	2.5	4	99.0 ± 0.1	59.3 ± 1.9
11	2.5	6	98.6 ± 1.0	63.6 ± 5.2
12	2.5	8	99.4 ± 0.2	53.1 ± 1.5

Supplementary Table 2: Reagents and conditions: sodium acetate buffer (250 mM, pH 5.5), substrate **1a** = 50 mM (4.91 mg per 500 µL), $Zn(SO_2CF_3)_2$ = 1.0-2.5 eq. (8.29 mg, 12.43 mg, 16.6 mg, 20.7 mg per 500 µL), *t*BuOOH 8 eq. (aq. solution: 27.5 µL per 500 µL); reaction time 24 hours, 30 °C, 900 rpm in Eppendorf orbital shaker (horizontal position), with syringe needle for air exchange in the top lid of the Eppendorf tube (2.0 mL).

entry	eq. $Zn(SO_2CF_3)_2$	eq. *t*BuOOH	recovery rate **1a** [%]	conv. **2a** [%]
1	1.0	8	98.9	--
2	1.5	8	98.9	--
3	2.0	8	97.9	--
4	2.5	8	95.8	--

Supplementary Table 3: Reagents and conditions: sodium acetate buffer (250 mM, pH 5.5), substrate **1a** = 50 mM (4.91 mg per 500 µL), $Zn(SO_2CF_3)_2$ = 2.0 eq. (16.6 mg per 500 µL), *t*BuOOH 8 eq. (aq. 70% wt solution: 27.5 µL per 500µL); 4.0 eq. TEMPO = (15.6 mg per 500 µL), reaction time 4-24 hours, 30 °C, 900 rpm in orbital shaker (horizontal position), with/without with syringe needle for air exchange in the top lid of the Eppendorf tube (2.0 mL).

entry	conditions and additives	time [h]	O_2[a]	conv. [%]	conv. **2a** [%]
1	Laccase + substrate **1a**	24	+	77.1 ± 0.3	<0.1
2	Laccase + substrate **1a** + $Zn(SO_2CF_3)_2$	24	+	23.6 ± 0.8	<0.1
3	Laccase + substrate **1a** + $Zn(SO_2CF_3)_2$ + *t*BuOOH	24	+	97.6 ± 0.5	55.6 ± 3.6
4	Laccase + substrate **1a** + $Zn(SO_2CF_3)_2$ + *t*BuOOH	24	-	99.1 ± 0.6	61.8 ± 0.4
5	Laccase + substrate **1a** + $Zn(SO_2CF_3)_2$ + *t*BuOOH	4	+	37.1 ± 4.2	1.8 ± 0.1

[a] Oxygen circulation was enabled through a syringe needle plugging in the tube lid

Supplementary Table 4: Comparison of specific activities of the laccase originating from *Agaricus bisporus* with and without *t*BuOOH.

entry	conditions	specific activity [U/mg]
1	Laccase + DMP	1.48
2	Laccase + DMP + *t*BuOOH (4.0 eq.)	1.91
3	Laccase + DMP + *t*BuOOH (6.0 eq.)	1.90
4	Laccase + DMP + *t*BuOOH (8.0 eq.)	1.84

Supplementary Table 5. Energies and free energy corrections of transition state structures of reaction of CF_3 radical with the phenol radical cation to the cationic intermediate (see **Supplementary Figure 4**).

Final product structure	CF_3-Substituent in position	E_0[a]	G correctionz
		(Hartree)[b]	(Hartree/particle)[b,d]
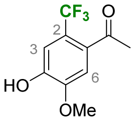 **2d**	C2	-911.78578572	0.146549
	C3	-911.78301018	0.146805
	C6	-911.78311549	0.146995
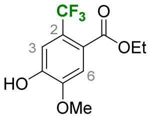 **2e**	C2	-1026.36189309	0.177154
	C3	-1026.35995007	0.177145
	C6	-1026.35205005	0.176513
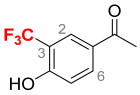 **2f**	C2	-797.31969701	0.117320
	C3	-797.34353827	0.116920

[a] Energy obtained from single-point calculations with M06-X2/6-311+G(d,p) scrf=(iefpcm, solvent=water)
[b] 1 Hartree = 627.51 kcal mol^{-1}
[c] Corrections obtained from frequency calculations on geometries optimized at the B3LYP/6-31g(d) level.
[d] Thermal corrections at 298.15 K.

Supplementary Table 6. Energies and free energy corrections of transition state structures of reaction of CF_3 radical with the phenol radical to the cationic intermediate (see **Supplementary Figure 4**).

Final product structure	CF_3-Substituent in position	E_0[a]	G correction[c]
		(Hartree)[b]	(Hartree/particle)[b,d]
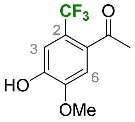 **2d**	C2	-911.35158597	0.133877
	C3	-911.35569041	0.133196

[a] Energy obtained from single-point calculations with M06-X2/6-311+G(d,p) scrf=(iefpcm, solvent=water)
[b] 1 Hartree = 627.51 kcal mol^{-1}
[c] Corrections obtained from frequency calculations on geometries optimized at the B3LYP/6-31g(d) level.
[d] Thermal corrections at 298.15 K.

Supplementary Table 7. Energies Transition states in **Supplementary Figure 5**

TS for C2

Charge = 1 Multiplicity = 1

C	-0.61293	-1.51237	-1.29257
H	1.64734	2.88222	0.70844
H	1.11162	3.28525	-0.92284
H	-0.52149	1.99429	0.48793
F	0.24519	-1.37464	1.54498
F	2.02766	-2.13042	0.55885
F	2.08675	-0.23427	1.57501
C	1.33161	-1.06519	0.88764
H	-0.64773	-2.47388	-1.78635
H	1.47829	-1.1553	-1.67267

C	-3.08353	1.97359	1.0199
H	-3.63375	-1.07078	-0.47224
O	-2.91903	0.71143	0.32473
C	1.88179	2.75816	-0.35268
O	2.87872	0.75991	-1.20577
C	1.90763	1.30577	-0.72785
O	-2.92269	-1.59619	-0.87854
C	-1.76902	-0.95972	-0.7772
C	-1.73106	0.33375	-0.11459
C	-0.54403	1.03246	-0.00148
C	0.63515	0.47574	-0.53114
C	0.58506	-0.80864	-1.17108
H	2.85537	3.19619	-0.56246
H	-2.80389	2.79551	0.36103
H	-4.13994	2.02465	1.26341
H	-2.48214	1.97337	1.92917

TS for C3

Charge = 1 Multiplicity = 1

C	-1.03736	-1.08268	0.92164
C	0.31978	-1.09623	1.26015
C	-1.57389	0.02829	0.28749
C	-0.77402	1.1544	-0.02744
C	0.55687	1.18597	0.31293
C	1.13341	0.04966	1.0048
O	2.32735	0.13946	1.55088
C	-3.04226	0.10013	-0.09934
O	-3.45044	1.09991	-0.6574
C	-3.93785	-1.0639	0.22381
O	1.43026	2.1723	0.13732
H	2.76032	0.96838	1.27736
C	1.01276	3.39177	-0.52261
H	-1.63448-	1.95286	1.15059
H	0.75703	-1.91	1.82371
C	1.42071	-1.35863	-0.67749
F	2.50119	-0.71344	-1.05158
F	0.46585	-1.26484	-1.55955
F	1.71286	-2.60287	-0.38556
H	-1.24783	1.99314	-0.51834
H	-4.95148	-0.83346	-0.09788
H	-3.59166	-1.96778	-0.28556
H	-3.93376-	1.26997	1.29768
H	1.90148	4.01401	-0.55718
H	0.6654	3.16588	-1.53117
H	0.22969	3.8769	0.06109

TS for C6

Charge = 1 Multiplicity = 1

C	-0.02235	2.28958	0.21161
C	-1.28013	1.67485	0.10639
C	-1.43766	0.36704	-0.34671
C	-0.29486	-0.35537	-0.71966
C	0.997	0.25598	-0.67598
C	1.11682	1.61609	-0.16777
O	2.30835	2.19691	-0.12897
C	-2.76467	-0.33135	-0.44893
O	-2.79509	-1.50212	-0.7873
C	-4.01602	0.4429	-0.13057

O	2.10319	-0.1974	-1.22103
H	2.98915	1.6162	-0.50821
C	2.13243	-1.44648	-1.97372
H	-0.41393	-1.34502	-1.14116
C	0.74562	-0.87886	1.21267
F	0.07128	-0.1888	2.08789
F	2.03779	-0.83556	1.43715
F	0.33711	-2.12471	1.13984
H	0.06337	3.31023	0.56071
H	-2.14698	2.25416	0.39685
H	-4.09411	1.33497	-0.75781
H	-4.8819	-0.1951	-0.2962
H	-4.00586	0.77354	0.91222
H	3.13996	-1.50123	-2.3722
H	1.94489	-2.28562	-1.30478
H	1.40117	-1.40594	-2.7791

Supplementary Table 8. Transition states in **Supplementary Figure 6**

TS for C2

Charge =1 Multiplicity = 1

C	-1.94556	-1.04974	-1.27378
H	0.26316	1.72245	0.44088
F	-1.1691	-1.41381	1.56777
F	-0.20415	-3.10683	0.60787
F	0.98913	-1.60885	1.59143
C	-0.11775	-1.83282	0.91587
H	-2.55509	-1.80576	-1.74931
H	-0.06066	-2.02847	-1.64808
C	-1.81173	3.25695	0.97232
H	-4.08728	1.1335	-0.47565
O	-2.4438	2.13954	0.29761
O	2.37056	0.75791	-0.24116
O	2.26215	-1.26654	-1.23431
C	1.74398	-0.30692	-0.71114
O	-3.83747	0.28152	-0.87394
C	-2.53281	0.09629	-0.77393
C	-1.72241	1.11737	-0.12904
C	-0.3536	0.96645	-0.02048
C	0.24256	-0.19826	-0.53442
C	-0.56489	-1.20703	-1.15755
H	-1.10619	3.74184	0.29772
H	-2.62465	3.93142	1.22161
H	-1.31507	2.90592	1.87696
C	3.83245	0.78614	-0.3658
C	4.31183	2.10092	0.20422
H	4.07595	0.67651	-1.4232
H	4.22628	-0.07338	0.1781
H	3.8923	2.94521	-0.3465
H	5.40042	2.14574	0.12213
H	4.0439	2.19488	1.25849

TS for C3

Charge = 1 Multiplicity = 1

C	-0.48629	-0.78951	1.11369
C	0.87291	-1.02131	1.34514
C	-0.87278	0.36814	0.4569
C	0.06859	1.3253	0.01104
C	1.41133	1.13925	0.24398

C	1.84368	-0.04747	0.95563
O	3.07943	-0.14214	1.39842
C	-2.32463	0.67142	0.17909
O	-2.68055	1.68244	-0.38529
O	-3.127	-0.28531	0.63171
O	2.41915	1.95037	-0.05799
H	3.6219	0.58385	1.04054
C	2.15899	3.18785	-0.76387
H	-1.21553	-1.51859	1.43276
H	1.21438	-1.87083	1.92209
C	1.73282	-1.56721	-0.65402
F	2.87984	-1.15415	-1.14058
F	0.74295	-1.33407	-1.46937
F	1.81281	-2.83046	-0.31277
H	-0.29673	2.21044	-0.49065
H	3.13275	3.64897	-0.89568
H	1.70432	2.97209	-1.73126
H	1.51284	3.82705	-0.16135
C	-4.56942	-0.12965	0.42997
C	-4.99457	-0.6966	-0.91106
H	-4.81556	0.92693	0.52641
H	-5.00507	-0.6829	1.2599
H	-4.54602	-0.1431	-1.73799
H	-6.0815	-0.6202	-0.99903
H	-4.71823	-1.74972	-0.99503

TS for C6
Charge = 1 Multiplicity = 1

C	0.50901	2.27106	0.14637
C	-0.71117	1.58545	0.04687
C	-0.78266	0.26029	-0.37309
C	0.39709	-0.41281	-0.71167
C	1.65293	0.27346	-0.67082
C	1.68902	1.65032	-0.19899
O	2.84561	2.29902	-0.16347
C	-2.0625	-0.50563	-0.46598
O	-2.10828	-1.67387	-0.7917
O	-3.11855	0.24219	-0.15753
O	2.78935	-0.13017	-1.19163
H	3.56178	1.74872	-0.52225
C	2.89642	-1.36726	-1.958
H	0.34128	-1.41848	-1.10696
C	1.4293	-0.8231	1.25621
F	0.75353	-0.09742	2.1015
F	2.72214	-0.75959	1.46654
F	1.02694	-2.07249	1.24835
H	0.53407	3.30476	0.46559
H	-1.62319	2.10544	0.30828
H	3.89642	-1.34014	-2.37805
H	2.79024	-2.22546	-1.29583
H	2.14788	-1.37526	-2.74828
C	-4.4324	-0.39678	-0.21776
C	-5.45961	0.63466	0.19156
H	-4.41954	-1.25623	0.454
H	-4.58667	-0.7523	-1.23761
H	-5.28044	0.98594	1.20993
H	-6.45338	0.18181	0.1557
H	-5.44953	1.4914	-0.48554

Supplementary Table 9. Transition states in **Supplementary Figure**

TS for C2

Charge = 1 Multiplicity = 1

C	1.62981	0.12529	1.17706
C	0.23421	0.08321	1.16775
C	-0.48559	-0.7081	0.21081
C	0.25485	-1.43105	-0.75077
C	1.62731	-1.37413	-0.74784
C	2.33238	-0.59688	0.21966
O	3.66184	-0.54445	0.24306
C	-1.9779	-0.92233	0.4349
O	-2.52101	-0.29645	1.32146
C	-2.6968	-1.91395	-0.43185
H	2.16103	0.70715	1.91805
H	-0.33046	0.53698	1.97211
C	-0.38588	1.4886	-0.40411
F	-0.24476	2.50271	0.404
F	-1.58586	1.46095	-0.91263
F	0.54857	1.42778 -	1.30012
H	-0.24895	-2.03829	1.48966
H	2.19204	-1.93325	-1.48575
H	4.06108	-1.07976	-0.45754
H	-2.26669	-2.91201	-0.30781
H	-3.74808	-1.93461	-0.15239
H	-2.6025	-1.64593	-1.48804

TS for C3

Charge = 1 Multiplicity = 1

C	0.5645	-0.05779	1.40174
C	-0.7709	-0.37422	1.14892
C	-1.47059	0.29557	0.15411
C	-0.82512	1.2975	-0.62189
C	0.47681	1.63777	-0.39413
C	1.21414	0.96682	0.63979
O	2.37555	1.51991	0.97985
C	-2.92974	-0.00238	-0.14902
O	-3.47918	0.63477	-1.02592
C	-3.63587	-1.06562	0.64434
H	1.10436	-0.55337	2.19993
H	-1.23494	-1.14767	1.74433
C	1.76241	-0.7997	-0.49478
F	2.30123	-1.73409	0.24441
F	2.67169	-0.1767	-1.1941
F	0.77623	-1.23266	-1.22031
H	-1.3981	1.80117	-1.38898
H	0.97462	2.42059	-0.95166
H	2.75377	1.1445	1.78911
H	-3.14887	-2.03512	0.50389
H	-3.6072	-0.83513	1.71306

Supplementary Table 10. Trifluoromethylation of phenols with $TMSCF_3$, $PhI(OAc)_2$ and catalytic silver fluoride.

entry	substrate	conv. [%][a]	ratio **3**:**2**[a]	**3** [%][a,b]
1	**1a** (R= Me)	79	1.48	28 (17)
2	**1b** (R = H)	69	1.09	17 (13)

[a] Determined by GC on an achiral phase.
[b] Isolated yields in brackets.

Supplementary Table 11. Trifluoromethylation of phenols with $NaSO_2CF_3$ and $PhI(CF_3CO_2)_2$ and catalytic silver.

entry	substrate	conv. [%][a]	**4a** [%][a,b]
1	**1a** (R= Me)	91	24 (18)
2	**1b** (R = H)	>99	93 (89)

[a] Determined by GC on a achiral phase.
[b Isolated yields in brackets.

Supplementary Table 12. Trifluoromethylation of phenol **1b** with 3,3-Dimethyl-1-(trifluoromethyl)-1,2-benziodoxole.

entry	substrate	conv. [%][a]	**4a** [%][b]
1	**1b** (R = Me)	>99	8

[a] Determined by GC-MS on an achiral phase.
[b] Area of product **4a** on GC-MS in comparison to the areas of the two unknown side products (peak areas of 58% and 34%).

Supplementary Table 13: Crystal data and structure refinement for 4-hydroxy-3,5-dimethoxy-2-(trifluoromethyl)-benzaldehyde (**2b**):

Empirical formula	$C_{10}H_9F_3O_4$	
Formula weight	250.17	
Temperature	100(2) K	
Wavelength	0.71073 A	
Crystal system, space group	Monoclinic, P2(1)	
Unit cell dimensions	a = 7.4098(13) A	alpha = 90 deg.
	b = 16.824(3) A	beta = 98.120(3) deg.
	c = 8.2667(15) A	gamma = 90 deg.
Volume	1020.2(3) A^3	
Z, Calculated density	2, 1.629 Mg/m^3	
Absorption coefficient	0.159 mm^-1	
F(000)	512	
Crystal size	0.37 x 0.22 x 0.12 mm	
Theta range for data collection	2.42 to 26.35 deg.	
Limiting indices	-9<=h<=9, -20<=k<=21, -10<=l<=10	
Reflections collected / unique	8120 / 4022 [R(int) = 0.0192]	
Completeness to theta =	26.35 / 99.9%	
Absorption correction	SADABS	
Max. and min. transmission	0.9812 and 0.9436	
Refinement method	Full-matrix least-squares on F^2	
Data / restraints / parameters	4022 / 1 / 314	
Goodness-of-fit on F^2	1.110	
Final R indices [I>2sigma(I)]	R1 = 0.0259, wR2 = 0.0637	
R indices (all data)	R1 = 0.0263, wR2 = 0.0640	
Largest diff. peak and hole	0.187 and -0.254 e.A^-3	

Supplementary Table 14: Bond lengths [A] and angles [deg] for4-hydroxy-3,5-dimethoxy-2-(trifluoromethyl)-benzaldehyde (**2b**):

F(1)-C(6)	1.3459(18)
F(2)-C(6)	1.3469(18)
F(3)-C(6)	1.3302(19)
F(4)-C(17)	1.3264(19)
F(5)-C(17)	1.3508(18)
F(6)-C(17)	1.3494(18)
O(1)-C(1)	1.3702(19)
O(1)-C(7)	1.436(2)
O(2)-C(2)	1.3450(18)
O(3)-C(3)	1.3632(18)
O(3)-C(8)	1.4411(19)
O(4)-C(10)	1.213(2)
O(5)-C(14)	1.3458(18)
O(6)-C(13)	1.3630(19)
O(6)-C(19)	1.4316(19)
O(7)-C(15)	1.3649(18)
O(7)-C(18)	1.4399(19)
O(8)-C(20)	1.218(2)
C(1)-C(2)	1.399(2)
C(1)-C(5)	1.401(2)
C(2)-C(3)	1.411(2)
C(3)-C(4)	1.381(2)
C(4)-C(9)	1.399(2)
C(5)-C(9)	1.407(2)
C(5)-C(6)	1.511(2)
C(9)-C(10)	1.482(2)
C(11)-C(16)	1.397(2)
C(11)-C(12)	1.413(2)
C(11)-C(20)	1.479(2)
C(12)-C(13)	1.402(2)
C(12)-C(17)	1.511(2)
C(13)-C(14)	1.407(2)
C(14)-C(15)	1.402(2)
C(15)-C(16)	1.380(2)
C(1)-O(1)-C(7)	113.91(12)
C(3)-O(3)-C(8)	115.95(11)
C(13)-O(6)-C(19)	115.20(11)
C(15)-O(7)-C(18)	115.96(11)
O(1)-C(1)-C(2)	117.61(13)
O(1)-C(1)-C(5)	121.98(14)
C(2)-C(1)-C(5)	120.29(14)
O(2)-C(2)-C(1)	117.17(13)
O(2)-C(2)-C(3)	122.88(13)
C(1)-C(2)-C(3)	119.95(13)
O(3)-C(3)-C(4)	125.49(14)
O(3)-C(3)-C(2)	114.89(13)
C(4)-C(3)-C(2)	119.60(14)
C(3)-C(4)-C(9)	120.73(14)
C(1)-C(5)-C(9)	119.14(14)
C(1)-C(5)-C(6)	121.88(14)
C(9)-C(5)-C(6)	118.95(13)
F(3)-C(6)-F(1)	106.40(13)
F(3)-C(6)-F(2)	105.63(12)
F(1)-C(6)-F(2)	105.70(12)
F(3)-C(6)-C(5)	115.11(13)
F(1)-C(6)-C(5)	112.28(13)
F(2)-C(6)-C(5)	111.06(12)
C(4)-C(9)-C(5)	120.13(14)
C(4)-C(9)-C(10)	116.48(14)
C(5)-C(9)-C(10)	123.35(14)
O(4)-C(10)-C(9)	123.11(15)
C(16)-C(11)-C(12)	120.01(13)
C(16)-C(11)-C(20)	116.59(14)
C(12)-C(11)-C(20)	123.39(14)
C(13)-C(12)-C(11)	119.17(13)
C(13)-C(12)-C(17)	121.91(13)
C(11)-C(12)-C(17)	118.92(13)
O(6)-C(13)-C(12)	121.88(13)
O(6)-C(13)-C(14)	117.84(13)
C(12)-C(13)-C(14)	120.02(14)
O(5)-C(14)-C(15)	122.75(14)
O(5)-C(14)-C(13)	117.19(13)
C(15)-C(14)-C(13)	120.03(13)
O(7)-C(15)-C(16)	125.31(14)
O(7)-C(15)-C(14)	114.72(13)
C(16)-C(15)-C(14)	119.95(14)
C(15)-C(16)-C(11)	120.78(14)
F(4)-C(17)-F(6)	106.15(13)
F(4)-C(17)-F(5)	106.03(13)
F(6)-C(17)-F(5)	105.38(12)
F(4)-C(17)-C(12)	115.46(13)
F(6)-C(17)-C(12)	111.60(12)
F(5)-C(17)-C(12)	111.53(12)
O(8)-C(20)-C(11)	122.60(14)

Supplementary Table 15: Torsion angles [deg] for 4-hydroxy-3,5-dimethoxy-2-(trifluoromethyl)benzaldehyde (**2b**):

C(7)-O(1)-C(1)-C(2)	78.82(17)
C(7)-O(1)-C(1)-C(5)	-104.93(16)
O(1)-C(1)-C(2)-O(2)	-1.03(19)
C(5)-C(1)-C(2)-O(2)	-177.35(13)
O(1)-C(1)-C(2)-C(3)	177.90(13)
C(5)-C(1)-C(2)-C(3)	1.6(2)
C(8)-O(3)-C(3)-C(4)	-2.9(2)
C(8)-O(3)-C(3)-C(2)	175.53(13)
O(2)-C(2)-C(3)-O(3)	-3.4(2)
C(1)-C(2)-C(3)-O(3)	177.76(13)
O(2)-C(2)-C(3)-C(4)	175.13(13)
C(1)-C(2)-C(3)-C(4)	-3.7(2)
O(3)-C(3)-C(4)-C(9)	-179.56(14)
C(2)-C(3)-C(4)-C(9)	2.1(2)
O(1)-C(1)-C(5)-C(9)	-173.98(13)
C(2)-C(1)-C(5)-C(9)	2.2(2)
O(1)-C(1)-C(5)-C(6)	7.7(2)
C(2)-C(1)-C(5)-C(6)	-176.17(13)
C(1)-C(5)-C(6)-F(3)	9.5(2)
C(9)-C(5)-C(6)-F(3)	-168.83(13)
C(1)-C(5)-C(6)-F(1)	-112.42(16)
C(9)-C(5)-C(6)-F(1)	69.23(17)
C(1)-C(5)-C(6)-F(2)	129.47(14)
C(9)-C(5)-C(6)-F(2)	-48.88(18)
C(3)-C(4)-C(9)-C(5)	1.7(2)
C(3)-C(4)-C(9)-C(10)	-176.13(13)
C(1)-C(5)-C(9)-C(4)	-3.8(2)
C(6)-C(5)-C(9)-C(4)	174.58(13)
C(1)-C(5)-C(9)-C(10)	173.84(14)
C(6)-C(5)-C(9)-C(10)	-7.8(2)
C(4)-C(9)-C(10)-O(4)	-5.6(2)
C(5)-C(9)-C(10)-O(4)	176.64(15)
C(16)-C(11)-C(12)-C(13)	-0.6(2)
C(20)-C(11)-C(12)-C(13)	177.65(14)
C(16)-C(11)-C(12)-C(17)	-179.72(13)
C(20)-C(11)-C(12)-C(17)	-1.5(2)
C(19)-O(6)-C(13)-C(12)	-105.85(16)
C(19)-O(6)-C(13)-C(14)	80.03(17)
C(11)-C(12)-C(13)-O(6)	171.74(13)
C(17)-C(12)-C(13)-O(6)	7.4(2)
C(11)-C(12)-C(13)-C(14)	2.3(2)
C(17)-C(12)-C(13)-C(14)	-178.65(14)
O(6)-C(13)-C(14)-O(5)	-6.1(2)
C(12)-C(13)-C(14)-O(5)	179.67(13)
O(6)-C(13)-C(14)-C(15)	171.89(13)
C(12)-C(13)-C(14)-C(15)	-2.3(2)
C(18)-O(7)-C(15)-C(16)	-12.1(2)
C(18)-O(7)-C(15)-C(14)	166.54(13)
O(5)-C(14)-C(15)-O(7)	-0.1(2)
C(13)-C(14)-C(15)-O(7)	-177.97(13)
O(5)-C(14)-C(15)-C(16)	178.60(14)
C(13)-C(14)-C(15)-C(16)	0.7(2)
O(7)-C(15)-C(16)-C(11)	179.51(13)
C(14)-C(15)-C(16)-C(11)	1.0(2)
C(12)-C(11)-C(16)-C(15)	-1.0(2)
C(20)-C(11)-C(16)-C(15)	-179.38(14)
C(13)-C(12)-C(17)-F(4)	2.9(2)
C(11)-C(12)-C(17)-F(4)	-178.03(13)
C(13)-C(12)-C(17)-F(6)	124.19(15)
C(11)-C(12)-C(17)-F(6)	-56.71(18)
C(13)-C(12)-C(17)-F(5)	-118.25(15)
C(11)-C(12)-C(17)-F(5)	60.86(18)
C(16)-C(11)-C(20)-O(8)	4.3(2)
C(12)-C(11)-C(20)-O(8)	-174.04(14)

Supplementary Table 16: Crystal data and structure refinement for ethyl 4-hydroxy-3,5-dimethoxy-2-(trifluoro-methyl)benzoate (**2c**):

Empirical formula	$C_{12}H_{13}F_3O_5$	
Formula weight	294.22	
Temperature	100(2) K	
Wavelength	0.71073 A	
Crystal system, space group	triclinic, P-1	
Unit cell dimensions	a = 7.9963(16) A	alpha = 106.608(3) deg.
	b = 12.755(2) A	beta = 90.848(3) deg.
	c = 13.024(3) A	gamma = 94.350(3) deg.
Volume	1268.4(4) A^3	
Z, Calculated density	2, 1.541 Mg/m^3	
Absorption coefficient	0.146 mm^-1	
F(000)	608	
Crystal size	0.44 x 0.10 x 0.07 mm	
Theta range for data collection	1.63 to 26.37 deg.	
Limiting indices	-9<=h<=9, -15<=k<=15, -16<=l<=16	
Reflections collected / unique	10224 / 5096 [R(int) = 0.0317]	
Completeness to theta =	26.37 / 98.6%	
Absorption correction	SADABS	
Max. and min. transmission	0.9898 and 0.9385	
Refinement method	Full-matrix least-squares on F^2	
Data / restraints / parameters	5096 / 0 / 369	
Goodness-of-fit on F^2	1.195	
Final R indices [I>2sigma(I)]	R1 = 0.0671, wR2 = 0.1220	
R indices (all data)	R1 = 0.0822, wR2 = 0.1280	
Largest diff. peak and hole	0.304 and -0.276 e.A^-3	

Supplementary Table 17: Bond lengths [A] and angles [deg] for ethyl 4-hydroxy-3,5-dimethoxy-2-(trifluoromethyl)-benzoate (**2c**):

F(1)-C(10)	1.351(3)	C(1)-C(2)-C(10)	121.9(2)
F(2)-C(10)	1.344(3)	C(3)-C(2)-C(10)	119.1(2)
F(3)-C(10)	1.337(3)	O(3)-C(3)-C(4)	118.9(2)
F(4)-C(24)	1.345(3)	O(3)-C(3)-C(2)	120.3(2)
F(5)-C(24)	1.346(3)	C(4)-C(3)-C(2)	120.7(2)
F(6)-C(24)	1.340(3)	O(4)-C(4)-C(3)	118.0(2)
O(1)-C(7)	1.211(3)	O(4)-C(4)-C(5)	122.5(2)
O(2)-C(7)	1.328(3)	C(3)-C(4)-C(5)	119.5(2)
O(2)-C(8)	1.456(3)	O(5)-C(5)-C(6)	125.7(2)
O(3)-C(3)	1.373(3)	O(5)-C(5)-C(4)	114.4(2)
O(3)-C(11)	1.447(3)	C(6)-C(5)-C(4)	119.9(2)
O(4)-C(4)	1.355(3)	C(5)-C(6)-C(1)	120.9(3)
O(5)-C(5)	1.363(3)	O(1)-C(7)-O(2)	124.1(2)
O(5)-C(12)	1.432(3)	O(1)-C(7)-C(1)	123.6(2)
O(6)-C(19)	1.213(3)	O(2)-C(7)-C(1)	112.2(2)
O(7)-C(19)	1.327(3)	O(2)-C(8)-C(9)	106.9(2)
O(7)-C(20)	1.463(3)	F(3)-C(10)-F(2)	106.7(2)
O(8)-C(15)	1.371(3)	F(3)-C(10)-F(1)	104.6(2)
O(8)-C(22)	1.452(3)	F(2)-C(10)-F(1)	106.9(2)
O(9)-C(16)	1.360(3)	F(3)-C(10)-C(2)	113.3(2)
O(10)-C(17)	1.360(3)	F(2)-C(10)-C(2)	112.5(2)
O(10)-C(23)	1.435(3)	F(1)-C(10)-C(2)	112.3(2)
C(1)-C(6)	1.390(4)	C(18)-C(13)-C(14)	120.5(2)
C(1)-C(2)	1.405(4)	C(18)-C(13)-C(19)	115.0(2)
C(1)-C(7)	1.496(4)	C(14)-C(13)-C(19)	124.5(2)
C(2)-C(3)	1.406(4)	C(13)-C(14)-C(15)	118.7(2)
C(2)-C(10)	1.503(4)	C(13)-C(14)-C(24)	122.1(2)
C(3)-C(4)	1.391(4)	C(15)-C(14)-C(24)	119.2(2)
C(4)-C(5)	1.401(4)	O(8)-C(15)-C(16)	119.9(2)
C(5)-C(6)	1.384(4)	O(8)-C(15)-C(14)	119.3(2)
C(8)-C(9)	1.504(4)	C(16)-C(15)-C(14)	120.6(2)
C(13)-C(18)	1.392(4)	O(9)-C(16)-C(15)	118.5(2)
C(13)-C(14)	1.399(4)	O(9)-C(16)-C(17)	121.7(2)
C(13)-C(19)	1.504(4)	C(15)-C(16)-C(17)	119.7(2)
C(14)-C(15)	1.405(4)	O(10)-C(17)-C(18)	125.1(2)
C(14)-C(24)	1.503(4)	O(10)-C(17)-C(16)	114.8(2)
C(15)-C(16)	1.388(4)	C(18)-C(17)-C(16)	120.0(2)
C(16)-C(17)	1.397(4)	C(17)-C(18)-C(13)	120.1(2)
C(17)-C(18)	1.388(4)	O(6)-C(19)-O(7)	124.7(2)
C(20)-C(21)	1.501(4)	O(6)-C(19)-C(13)	123.4(2)
C(7)-O(2)-C(8)	116.2(2)	O(7)-C(19)-C(13)	111.7(2)
C(3)-O(3)-C(11)	113.82(19)	O(7)-C(20)-C(21)	110.9(2)
C(5)-O(5)-C(12)	117.0(2)	F(6)-C(24)-F(4)	105.1(2)
C(19)-O(7)-C(20)	117.2(2)	F(6)-C(24)-F(5)	106.5(2)
C(15)-O(8)-C(22)	114.3(2)	F(4)-C(24)-F(5)	106.7(2)
C(17)-O(10)-C(23)	117.4(2)	F(6)-C(24)-C(14)	113.0(2)
C(6)-C(1)-C(2)	119.7(2)	F(4)-C(24)-C(14)	112.3(2)
C(6)-C(1)-C(7)	115.3(2)	F(5)-C(24)-C(14)	112.7(2)
C(2)-C(1)-C(7)	124.9(2)		
C(1)-C(2)-C(3)	119.0(2)		

Supplementary Table 18: Torsion angles [deg] for ethyl 4-hydroxy-3,5-dimethoxy-2-(trifluoromethyl)benzoate (**2c**):

C(6)-C(1)-C(2)-C(3)	-4.2(4)
C(7)-C(1)-C(2)-C(3)	174.0(2)
C(6)-C(1)-C(2)-C(10)	173.9(2)
C(7)-C(1)-C(2)-C(10)	-7.9(4)
C(11)-O(3)-C(3)-C(4)	76.7(3)
C(11)-O(3)-C(3)-C(2)	-106.8(3)
C(1)-C(2)-C(3)-O(3)	-173.0(2)
C(10)-C(2)-C(3)-O(3)	8.9(4)
C(1)-C(2)-C(3)-C(4)	3.5(4)
C(10)-C(2)-C(3)-C(4)	-174.7(2)
O(3)-C(3)-C(4)-O(4)	-2.6(4)
C(2)-C(3)-C(4)-O(4)	-179.1(2)
O(3)-C(3)-C(4)-C(5)	177.1(2)
C(2)-C(3)-C(4)-C(5)	0.6(4)
C(12)-O(5)-C(5)-C(6)	-1.5(4)
C(12)-O(5)-C(5)-C(4)	177.6(2)
O(4)-C(4)-C(5)-O(5)	-3.4(4)
C(3)-C(4)-C(5)-O(5)	176.9(2)
O(4)-C(4)-C(5)-C(6)	175.8(2)
C(3)-C(4)-C(5)-C(6)	-3.9(4)
O(5)-C(5)-C(6)-C(1)	-177.8(2)
C(4)-C(5)-C(6)-C(1)	3.2(4)
C(2)-C(1)-C(6)-C(5)	0.9(4)
C(7)-C(1)-C(6)-C(5)	-177.4(2)
C(8)-O(2)-C(7)-O(1)	-4.2(4)
C(8)-O(2)-C(7)-C(1)	178.5(2)
C(6)-C(1)-C(7)-O(1)	-42.2(4)
C(2)-C(1)-C(7)-O(1)	139.5(3)
C(6)-C(1)-C(7)-O(2)	135.1(2)
C(2)-C(1)-C(7)-O(2)	-43.2(3)
C(7)-O(2)-C(8)-C(9)	-174.4(2)
C(1)-C(2)-C(10)-F(3)	-146.2(2)
C(3)-C(2)-C(10)-F(3)	31.9(4)
C(1)-C(2)-C(10)-F(2)	92.6(3)
C(3)-C(2)-C(10)-F(2)	-89.3(3)
C(1)-C(2)-C(10)-F(1)	-28.0(4)
C(3)-C(2)-C(10)-F(1)	150.1(2)
C(18)-C(13)-C(14)-C(15)	-4.8(4)
C(19)-C(13)-C(14)-C(15)	173.3(2)
C(18)-C(13)-C(14)-C(24)	172.1(2)
C(19)-C(13)-C(14)-C(24)	-9.9(4)
C(22)-O(8)-C(15)-C(16)	74.0(3)
C(22)-O(8)-C(15)-C(14)	-110.8(3)
C(13)-C(14)-C(15)-O(8)	-172.2(2)
C(24)-C(14)-C(15)-O(8)	10.8(4)
C(13)-C(14)-C(15)-C(16)	3.0(4)
C(24)-C(14)-C(15)-C(16)	-174.0(2)
O(8)-C(15)-C(16)-O(9)	-2.9(4)
C(14)-C(15)-C(16)-O(9)	-178.1(2)
O(8)-C(15)-C(16)-C(17)	177.0(2)
C(14)-C(15)-C(16)-C(17)	1.8(4)
C(23)-O(10)-C(17)-C(18)	-2.2(4)
C(23)-O(10)-C(17)-C(16)	176.7(2)
O(9)-C(16)-C(17)-O(10)	-4.0(4)
C(15)-C(16)-C(17)-O(10)	176.1(2)
O(9)-C(16)-C(17)-C(18)	175.0(2)
C(15)-C(16)-C(17)-C(18)	-4.9(4)
O(10)-C(17)-C(18)-C(13)	-178.0(2)
C(16)-C(17)-C(18)-C(13)	3.1(4)
C(14)-C(13)-C(18)-C(17)	1.8(4)
C(19)-C(13)-C(18)-C(17)	-176.4(2)
C(20)-O(7)-C(19)-O(6)	-0.8(4)
C(20)-O(7)-C(19)-C(13)	-176.0(2)
C(18)-C(13)-C(19)-O(6)	-47.0(4)
C(14)-C(13)-C(19)-O(6)	134.8(3)
C(18)-C(13)-C(19)-O(7)	128.3(2)
C(14)-C(13)-C(19)-O(7)	-49.9(3)
C(19)-O(7)-C(20)-C(21)	-87.2(3)
C(13)-C(14)-C(24)-F(6)	-140.3(3)
C(15)-C(14)-C(24)-F(6)	36.6(3)
C(13)-C(14)-C(24)-F(4)	-21.7(4)
C(15)-C(14)-C(24)-F(4)	155.2(2)
C(13)-C(14)-C(24)-F(5)	98.9(3)
C(15)-C(14)-C(24)-F(5)	-84.3(3)

Supplementary Table 19: Crystal data and structure refinement for 1-(4-hydroxy-5-methoxy-2-(trifluoromethyl)-phenylethanone (**2d**):

Empirical formula	$C_{20}H_{18}F_6O_6$	
Formula weight	468.34	
Temperature	100(2) K	
Wavelength	0.71073 A	
Crystal system, space group	Triclinic, P1	
Unit cell dimensions	a = 13.598(6) A	alpha = 90 deg.
	b = 17.053(7) A	beta = 90 deg.
	c = 17.163(7) A	gamma = 90 deg.
Volume	3980(3) A^3	
Z, Calculated density	8, 1.563 Mg/m^3	
Absorption coefficient	0.150 mm^-1	
F(000)	1920	
Crystal size	0.22 x 0.15 x 0.08 mm	
Theta range data collection	2.25 to 26.38 deg.	
Limiting indices	16<=h<=16, -21<=k<=21, -21<=l<=21	
Reflections collected / unique	29386 / 4066 [R(int) = 0.1405]	
Completeness to theta =	26.38 / 99.9%	
Absorption correction	None	
Max. and min. transmission	0.9881 and 0.9678	
Refinement method	Full-matrix least-squares on F^2	
Data / restraints / parameters	4066 / 0 / 295	
Goodness-of-fit on F^2	1.057	
Final R indices [I>2sigma(I)]	R1 = 0.0615, wR2 = 0.1481	
R indices (all data)	R1 = 0.0695, wR2 = 0.1543	
Largest diff. peak and hole	0.395 and -0.611 e.A^-3	

Supplementary Table 20: Bond lengths [A] and angles [deg] for 1-(4-hydroxy-5-methoxy-2-(trifluoromethyl)-phenylethanone (**2d**):

O(1)-C(7)	1.219(3)
O(2)-C(4)	1.353(3)
O(3)-C(5)	1.358(3)
O(3)-C(10)	1.438(3)
O(4)-C(17)	1.217(3)
O(5)-C(14)	1.347(3)
O(6)-C(15)	1.353(3)
O(6)-C(20)	1.438(3)
F(1)-C(9)	1.343(3)
F(2)-C(9)	1.338(3)
F(3)-C(9)	1.350(3)
F(4)-C(19)	1.351(3)
F(5)-C(19)	1.341(3)
F(6)-C(19)	1.347(3)
C(1)-C(6)	1.401(3)
C(1)-C(2)	1.411(3)
C(1)-C(7)	1.495(3)
C(2)-C(3)	1.397(3)
C(2)-C(9)	1.510(3)
C(3)-C(4)	1.379(3)
C(4)-C(5)	1.406(3)
C(5)-C(6)	1.381(3)
C(7)-C(8)	1.505(3)
C(11)-C(16)	1.399(3)
C(11)-C(12)	1.415(3)
C(11)-C(17)	1.493(3)
C(12)-C(13)	1.387(3)
C(12)-C(19)	1.504(3)
C(13)-C(14)	1.380(3)
C(14)-C(15)	1.417(3)
C(15)-C(16)	1.378(3)
C(17)-C(18)	1.515(3)
C(5)-O(3)-C(10)	117.30(17)
C(15)-O(6)-C(20)	116.70(17)
C(6)-C(1)-C(2)	118.3(2)
C(6)-C(1)-C(7)	117.9(2)
(2)-C(1)-C(7)	123.8(2)
C(3)-C(2)-C(1)	119.4(2)
C(3)-C(2)-C(9)	116.5(2)
C(1)-C(2)-C(9)	123.9(2)
C(4)-C(3)-C(2)	121.4(2)
O(2)-C(4)-C(3)	118.8(2)
O(2)-C(4)-C(5)	121.7(2)
C(3)-C(4)-C(5)	119.5(2)
O(3)-C(5)-C(6)	125.9(2)
O(3)-C(5)-C(4)	114.70(19)
C(6)-C(5)-C(4)	119.4(2)
C(5)-C(6)-C(1)	121.9(2)
O(1)-C(7)-C(1)	121.0(2)
O(1)-C(7)-C(8)	119.8(2)
C(1)-C(7)-C(8)	119.15(19)
F(2)-C(9)-F(1)	107.56(18)
F(2)-C(9)-F(3)	105.38(18)
F(1)-C(9)-F(3)	105.04(19)
F(2)-C(9)-C(2)	114.38(19)
F(1)-C(9)-C(2)	112.74(18)
F(3)-C(9)-C(2)	111.06(18)
C(16)-C(11)-C(12)	118.0(2)
C(16)-C(11)-C(17)	118.66(19)
C(12)-C(11)-C(17)	123.3(2)
C(13)-C(12)-C(11)	119.6(2)
C(13)-C(12)-C(19)	117.23(19)
C(11)-C(12)-C(19)	123.1(2)
C(14)-C(13)-C(12)	121.9(2)
O(5)-C(14)-C(13)	119.06(19)
O(5)-C(14)-C(15)	121.9(2)
C(13)-C(14)-C(15)	119.0(2)
O(6)-C(15)-C(16)	126.29(19)
O(6)-C(15)-C(14)	114.56(19)
C(16)-C(15)-C(14)	119.1(2)
C(15)-C(16)-C(11)	122.3(2)
O(4)-C(17)-C(11)	121.2(2)
O(4)-C(17)-C(18)	120.5(2)
C(11)-C(17)-C(18)	118.32(19)
F(5)-C(19)-F(6)	105.20(18)
F(5)-C(19)-F(4)	107.42(18)
F(6)-C(19)-F(4)	105.46(18)
F(5)-C(19)-C(12)	113.89(18)
F(6)-C(19)-C(12)	111.38(18)
F(4)-C(19)-C(12)	112.86(19)

Supplementary Table 21: Torsion angles [deg] for 1-(4-hydroxy-5-methoxy-2-(trifluoromethyl)phenylethanone (**2d**):

C(6)-C(1)-C(2)-C(3)	3.4(3)
C(7)-C(1)-C(2)-C(3)	-174.46(19)
C(6)-C(1)-C(2)-C(9)	-172.8(2)
C(7)-C(1)-C(2)-C(9)	9.3(3)
C(1)-C(2)-C(3)-C(4)	-1.8(3)
C(9)-C(2)-C(3)-C(4)	174.68(19)
C(2)-C(3)-C(4)-O(2)	179.75(19)
C(2)-C(3)-C(4)-C(5)	-0.9(3)
C(10)-O(3)-C(5)-C(6)	-2.0(3)
C(10)-O(3)-C(5)-C(4)	178.08(18)
O(2)-C(4)-C(5)-O(3)	1.3(3)
C(3)-C(4)-C(5)-O(3)	-178.03(18)
O(2)-C(4)-C(5)-C(6)	-178.67(18)
C(3)-C(4)-C(5)-C(6)	2.0(3)
O(3)-C(5)-C(6)-C(1)	179.69(19)
C(4)-C(5)-C(6)-C(1)	-0.4(3)
C(2)-C(1)-C(6)-C(5)	-2.3(3)
C(7)-C(1)-C(6)-C(5)	175.65(19)
C(6)-C(1)-C(7)-O(1)	-163.2(2)
C(2)-C(1)-C(7)-O(1)	14.6(3)
C(6)-C(1)-C(7)-C(8)	14.2(3)
C(2)-C(1)-C(7)-C(8)	-168.0(2)
C(3)-C(2)-C(9)-F(2)	107.6(2)
C(1)-C(2)-C(9)-F(2)	-76.1(3)
C(3)-C(2)-C(9)-F(1)	-129.1(2)
C(1)-C(2)-C(9)-F(1)	47.2(3)
C(3)-C(2)-C(9)-F(3)	-11.5(3)
C(1)-C(2)-C(9)-F(3)	164.8(2)
C(16)-C(11)-C(12)-C(13)	0.2(3)
C(17)-C(11)-C(12)-C(13)	178.34(19)
C(16)-C(11)-C(12)-C(19)	177.58(19)
C(17)-C(11)-C(12)-C(19)	-4.3(3)
C(11)-C(12)-C(13)-C(14)	-1.5(3)
C(19)-C(12)-C(13)-C(14)	-179.1(2)
C(12)-C(13)-C(14)-O(5)	179.47(19)
C(12)-C(13)-C(14)-C(15)	0.9(3)
C(20)-O(6)-C(15)-C(16)	3.4(3)
C(20)-O(6)-C(15)-C(14)	-175.92(18)
O(5)-C(14)-C(15)-O(6)	1.9(3)
C(13)-C(14)-C(15)-O(6)	-179.51(19)
O(5)-C(14)-C(15)-C(16)	-177.46(19)
C(13)-C(14)-C(15)-C(16)	1.1(3)
O(6)-C(15)-C(16)-C(11)	178.2(2)
C(14)-C(15)-C(16)-C(11)	-2.5(3)
C(12)-C(11)-C(16)-C(15)	1.8(3)
C(17)-C(11)-C(16)-C(15)	-176.42(19)
C(16)-C(11)-C(17)-O(4)	152.6(2)
C(12)-C(11)-C(17)-O(4)	-25.6(3)
C(16)-C(11)-C(17)-C(18)	-27.1(3)
C(12)-C(11)-C(17)-C(18)	154.7(2)
C(13)-C(12)-C(19)-F(5)	-108.1(2)
C(11)-C(12)-C(19)-F(5)	74.4(3)
C(13)-C(12)-C(19)-F(6)	10.7(3)
C(11)-C(12)-C(19)-F(6)	-166.80(19)
C(13)-C(12)-C(19)-F(4)	129.1(2)
C(11)-C(12)-C(19)-F(4)	-48.4(3)

Supplementary Methods

1. General Information

All starting materials were obtained from commercial suppliers and used as received unless stated otherwise, e.g. *t*BuOOH was obtained as 70 wt% in water from Aldrich. Chemical reactions were carried out with standard *Schlenk*-techniques under N_2 atmosphere in oven-dried (125°C) glassware. Solvents were dried and purified by conventional methods prior to use. Preparative chromatographic separations were performed by column chromatography on silica gel 60 (0.063–0.200 mm). Solvents for flash chromatography (petroleum ether/ethyl acetate/dichloromethane) were distilled before use. Petroleum ether refers to a fraction with a boiling point between 63–69 °C. TLC was carried out with pre-coated aluminium sheets with detection by UV (254 nm) and/or by staining with p-anisaldehyde solution or cerium molybdenum solution. ^1H and ^{13}C NMR spectra were recorded at 20 °C; chemical shifts are given in ppm relative to Me_4Si (^1H: Me_4Si = 0.0 ppm) or relative to the resonance of the solvent (^1H: $CDCl_3$ = 7.26 ppm; ^{13}C: $CDCl_3$ = 77.0 ppm). Laccase originating from *Agaricus bisporus* (1.5 U mg^{-1}) was bought in form of a freeze dried powder from ASA Spezialenzyme GmbH (Germany) and used as received. The specific activity was determined according to an enzyme assay by J. F. Kennedy and co-workers. For measurements please see Supplementary methods and tables. HRMS were recorded on an HPLC-TOF-MS system consisting of an HPLC [Agilent 1260 Infinity Series; Injection: 0.1 µL; eluent: isocratic, 20% H_2O, 80% (90% ACN with 10%H_2O (0.1% 5 M Ammonium formate)), flow: 0.3 mL min^{-1}] and Agilent 6230 TOF LC/MS with APCI measuring in the negative mode and the following settings: gas temp. (N_2): 350 °C; vaporizer: 375 °C; drying gas: 10 L min^{-1}; nebulizer: 40 psig; fragmentor: 50 V; skimmer: 65 V; OCT 1 RF Vpp: 750 V; Vcap: 3500 V; corona: 22 µA; nozzle voltage: 1100V; reference masses: 966.0007250; acquisition: 150-1,100 m z^{-1}; 1 spectra s^{-1}.

2. Preliminary Experiments (Analytical Scale)

Due to undesired polymerisation of the phenols catalysed by the laccase both, the substrate **1a** and product **2a**, were calibrated for initial conversion measurements; in order to ensure most possible accuracy, the compounds for calibration were treated similar to the biotransformations.

For calibration curves see Supplementary Figure 1 and Supplementary Figure 2.

General procedure: Out of a stock solution of **1a** or **2a** (50 mM in DMSO) certain aliquots were withdrawn and diluted with a sodium acetate buffer (250 mM, pH 5.5) and DMSO to reach a total

volume of 500 µL containing 25 vol% DMSO. The mixtures were shaken for 8 hours at 30 °C in Eppendorf tubes (2.0 mL) in an orbital shaker (horizontal position) and were then diluted with saturated NaCl solution (400 µL). The mixtures were extracted twice with EtOAc (500 µL) and combined organic layers dried over $MgSO_4$. The samples were filtered and used for GC measurements.

2.1. Enzymatic Trifluoromethylation at Varied Amounts of Zinc trifluoromethanesulfinate $Zn(SO_2CF_3)_2$ and *t*BuOOH

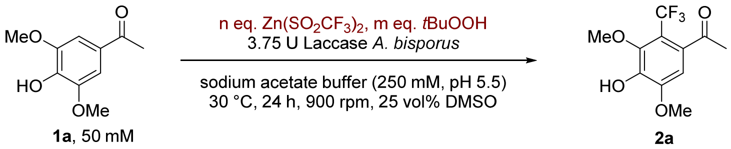

General procedure: Varied amounts of $Zn(SO_2CF_3)_2$ (dissolved in DMSO) were added to a sodium acetate buffer (250 mM, pH 5.5) containing the laccase from *A. bisporus* (2.5 mg/3.75 U) in an Eppendorf tube (2.0 mL), followed by the substrate **1a** (50 mM, dissolved in DMSO) and varied amounts of aq. *t*BuOOH solution to reach a total volume of 500 µL (25 vol% DMSO). Air access was enabled through a syringe needle punched in the lid of the tube. The reactions were shaken in an orbital shaker (horizontal position) at 30 °C for 24 hours (900 rpm) and were then diluted with saturated NaCl solution (400 µL). The mixtures were extracted twice with an exact amount of EtOAc (500 µL). Combined organic layers were dried over $MgSO_4$, filtrated and measured via GC on an achiral phase.

Determination of conversion by GC: HP-5 column (Agilent Technologies), temperature program: 100-300 °C, slope = 10 °C/min. R_t (**2a**) = 9.93 min, R_t (**1a**) = 10.73 min.

For results see Supplementary Table 1.

2.2. Control Experiments - Blank Reactions

Procedure: Varied amounts of Zinc trifluoromethanesulfinate $Zn(SO_2CF_3)_2$ (dissolved in DMSO) were added to a sodium acetate buffer (250 mM, pH 5.5) in an Eppendorf tube (2.0 mL), followed by the substrate **1a** (4.91 mg, dissolved in DMSO, 50 mM final conc.) and aqueous *t*BuOOH solution (27.5 µL, 70 wt%) to reach a total volume of 500 µL (25 vol% DMSO). Air access was enabled through a syringe needle punched in the lid of the tube. The reactions were shaken in an orbital shaker at 30 °C for 24 hours (900 rpm) and were then diluted with saturated NaCl solution (400 µL). The mixtures were extracted twice with an exact amount of EtOAc (500 µL) and the combined organic layers were dried ($MgSO_4$). The solution was filtrated and analysed by GC.

For results see Supplementary Table 2.

2.3. Control Experiments – Influence of Diverse Additives on the Enzymatic Trifluoromethylation Reaction

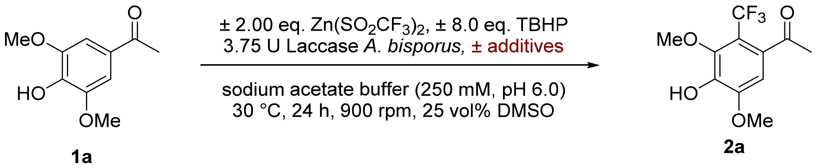

General procedure: Laccase from *A. bisporus* (2.5 mg/3.75 U) was dissolved in a sodium acetate buffer (250 mM, pH 5.5) in an Eppendorf tube (2.0 mL) prior to addition of reagents [($Zn(SO_2CF_3)_2$ (2 eq., 100 mM, 16.6 mg per 500 µL) and/or TEMPO (4 eq., 200 mM, 15.62 mg per 500 µL)] dissolved in DMSO. Afterwards aryl-ketone **1a** in DMSO (50 mM, 4.91 mg per 500 µL) was added followed by aqueous *t*BuOOH solution (8 eq., 200 mM, 27.5 µL per 500 µL). The reactions were shaken in the pre- and absence of a syringe needle in the top lid for access of air in an orbital shaker at 30 °C for the time indicated in Supplementary Table 3. Then, 400 µL saturated NaCl solution were added and the reactions extracted twice with an exact amount of 500 µL EtOAc. Combined organic layers were dried over $MgSO_4$, filtrated and used for GC-measurements.

For results see Supplementary Table 3.

2.4. Influence of Organic Solvent and Temperature

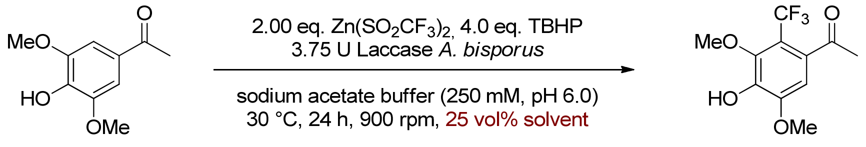

General procedure: The laccase from *A. bisporus* (2.5 mg per 500 µL) was dissolved in a sodium acetate buffer (250 mM, pH 5.5) in an Eppendorf tube (2.0 mL) prior to addition of $Zn(SO_2CF_3)_2$ (2 eq., 100 mM, 16.6 mg per 500 µL, dissolved in the corresponding solvent shown in Supplementary Figure 3). In case of MeCN, EtOH and acetone, the zinc trifluoromethanesulfinate $Zn(SO_2CF_3)_2$ solutions were warmed to 40 °C for solubility reasons prior to addition to the enzyme solution. Afterwards ketone **1a** (50 mM, 4.91 mg per 500 µL, dissolved in the solvent shown in figure 3) was added followed by aqueous *t*BuOOH solution (8 eq., 400 mM, 27.5 µL per 500µL). The reactions were shaken with a syringe needle in the top lid for access of air in an orbital shaker at 30 °C for 24 hours. Then, 400 µL of saturated NaCl solution were added and the reactions extracted twice with exactly 500 µL EtOAc. Combined organic layers were dried over $MgSO_4$, filtrated and used for GC-measurements.

For results see Supplement Figure 3.

3. Photometric Enzymatic Activity Assay

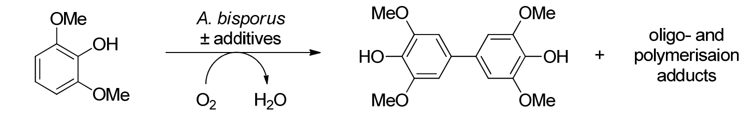

Dimethoxyphenol
(DMP)

3,3',5,5'-tetramethoxy-1,1'-biphenyl-4,4'-diol
(TMBP)

The enzymatic activity of the laccase originating from *A. bisporus* (0.5 mg mL^{-1}) was determined spectrophotometrically (λ = 469 nm) for four minutes in triplicate at room temperature according to a literature procedure.[1] 2,6-Dimethoxyphenol (0.83 mg mL^{-1}) was employed as model substrate with deionized water (pH 6.0) as reaction medium. The amount of DMSO was 25 vol% to ensure authentic reaction conditions. One unit was defined as the change in optical density a the corresponding wavelength effected per min per µmol of protein added to 1 mL substrate solution in a 1 cm path length (plastic cuvette) incubated at 25 °C [ε_{469} (DMP) = 49.600 M^{-1} cm^{-1}].[2]

For results see Supplement Figure 4.

4. Biotransformations on Preparative Scale

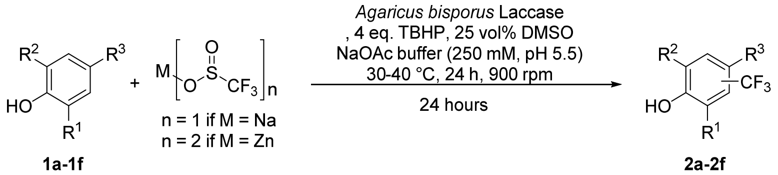

1a-1f **2a-2f**

Four to ten 1 mL reactions were run in parallel; after reaction and extraction, the organic layers were combined and the products were purified as described.

Representative Procedure: The laccase from *A. bisporus* (5.0 mg mL^{-1}) was dissolved in a sodium acetate buffer (250 mM, pH 5.5) in an Eppendorf tube (2.0 mL) prior to addition of $Zn(SO_2CF_3)_2$ (2 eq., dissolved in DMSO) or $NaSO_2CF_3$ (2. eq, dissolved in DMSO). Afterwards ketone **1** (50 mM, dissolved in DMSO) was added followed by aqueous *t*BuOOH solution (8.0 eq.) to reach a total volume of 1 mL (25 vol% DMSO). The reactions were shaken in an orbital shaker at 30 °C [$Zn(SO_2CF_3)_2$] or 40 °C (in case of $NaSO_2CF_3$) for 24 hours at 900 rpm (horizontal position). Then, each reaction was extracted four times with EtOAc (500 µL) and combined organic fractions were dried over Na_2SO_4. The solutions were filtered, concentrated under reduced pressure and the residue purified by silica gel chromatography to afford the trifluoromethylated phenol derivative **2**.

1-(4-Hydroxy-3,5-dimethoxy-2-(trifluoromethyl)phenyl)ethanone (2a)

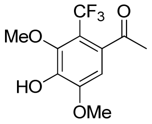

2a

Following the representative procedure, five reactions were run in parallel with ketone **1a** (9.81 mg mL^{-1}) as substrate and Baran's zinc sulfinate as trifluoromethylating reagent. Chromatography on silica (eluent: PE/EtOAc 70:30) afforded the product **2a** as colourless solid in 61.6% yield (40.7 mg, 0.154 mmol). R_F (PE/EtOAc 70:30) = 0.26; melting range = 115-118 °C; ^{1}H-NMR (300 MHz, $CDCl_3$): δ_H [ppm] = 2.46 (s, 3 H, COC*H*$_3$), 3.93 (s, 3 H, OCH_3), 3.95 (s, 3 H, OCH_3), 5.90 (brs, 1 H, OH), 6.50 (s, 1 H, 6-H); ^{13}C-NMR (75 MHz, $CDCl_3$): δ_C [ppm] = 31.6 (C-2'), 56.5 (OCH_3), 61.5 (OCH_3), 103.8 (q, $^4J_{C6,F}$ = 1.6 Hz, C-6), 113.5 (q, $^2J_{C2,F}$ = 31.1 Hz, C-2), 123.5 (q, $^1J_{CF3,F}$ = 273.6 Hz, CF_3), 133.7 (q, $^3J_{1C,F}$ = 2.7 Hz, C-1), 140.2 (C-4), 145.9 (q, $^3J_{3C,F}$ = 2.0 Hz, C-3), 150.0 (C-5), 202.7 (C-1'); ^{19}F-NMR (272 MHz, $CDCl_3$): δ_F [ppm] = -54.7 (s, 3 F, CF_3); GC-MS (EI, 70 eV): *m z*$^{-1}$ [%] = 264 [M^+] (52), 249 [$C_{10}H_8F_3O_4^+$] (100), 43 [$C_2H_3O^+$] (9); IR (ATR-film): $\tilde{\nu}_{max}$ [cm^{-1}] = 3409 (OH), 2957, 1692 (C=O), 1601, 1302, 1091 (C-F), 611. HR-MS: *m z*$^{-1}$ = 263.0537 [$(M-H)^-$] (calcd.: 263.0537).

Following the representative procedure, ten reactions were run in parallel with ketone **1a** (9.81 mg mL^{-1}) as substrate using Langlois' reagent for the trifluoromethylation. Chromatography on silica afforded the product **2a** in 52.5% yield (69.3 mg, 0.262 mmol).

4-Hydroxy-3,5-dimethoxy-2-(trifluoromethyl)benzaldehyde (2b)

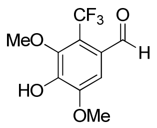

2b [x-ray]

Following the representative procedure, six reactions were run in parallel with ketone **1b** (9.10 mg mL^{-1}) as substrate and Baran's zinc sulfinate as trifluoromethylating reagent. Chromatography on silica (eluent: PE/EtOAc 80:20) afforded the product **2b** as colourless oil in 42.2% yield (31.7 mg, 0.127 mmol) which slowly crystallises upon standing. R_F (PE/EtOAc 70:30) = 0.40; melting range = 112-114 °C; ^{1}H-NMR (300 MHz, $CDCl_3$): δ_H [ppm] = 3.95 (s, 3 H, OCH_3), 4.00 (s, 3 H, OCH_3), 6.30 (brs, 1 H, OH), 7.40 (s, 1 H, 6 H), 10.29 (q, $^4J_{CHO,F}$ = 2.3 Hz, 1 H, C*H*O); ^{13}C-NMR (75 MHz, $CDCl_3$): δ_C [ppm] = 56.7 (OCH_3), 61.9 (OCH_3), 106.4 (C-6), 118.9 (q, $^2J_{C2,F}$ = 31.4 Hz, C-2), 124.5 (q, $^1J_{CF3,F}$ = 275.9 Hz, CF_3), 127.4 (q, $^3J_{C1,F}$ = 1.0 Hz, C-1), 144.4 (C-4), 145.9 (q, $^3J_{3C,F}$ = 2.5 Hz, C-3), 149.6 (C-5), 189.0 (q, $^4J_{CHO,F}$ = 5.9 Hz, *C*HO); ^{19}F-NMR (275 MHz, $CDCl_3$): δ_F [ppm] = -50.6 (d, $J_{F,CHO}$= 2.2 Hz, F, CF_3); GC-MS (EI, 70 eV): $m\ z^{-1}$ [%] = 250 [M^+] (100); IR (ATR-film): $\tilde{\nu}_{max}$ [cm^{-1}] = 3197 (OH), 2951, 1669 (C=O), 1305, 1092 (C-F), 592. HR-MS: $m\ z^{-1}$ = 249.0379 [$(M-H)^-$] (calcd.: 249.0380).

Following the representative procedure, ten reactions were run in parallel with ketone **1b** (9.10 mg mL^{-1}) as substrate using Langlois' reagent for the trifluoromethylation. Chromatography on silica afforded the product **2b** in 40.5% yield (45.6 mg, 0.182 mmol).

Ethyl 4-hydroxy-3,5-dimethoxy-2-(trifluoromethyl)benzoate (2c)

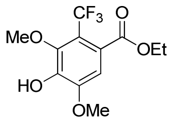

2c [x-ray]

Following the representative procedure, six reactions were run in parallel with ketone **1c** (11.3 mg mL^{-1}) as substrate and Baran's zinc sulfinate as trifluoromethylating reagent. Chromatography on silica (eluent: CH_2Cl_2) afforded the product **2c** as colourless solid 57.8% yield (51.0 mg, 0.173 mmol). R_F (PE/EtOAc 70:30) = 0.35; melting range = 108.4-109 °C; ^{1}H-NMR (300 MHz, $CDCl_3$): δ_H [ppm] = 1.35

(t, $^3J_{CH3,CH2}$ = 7.2 Hz, 3 H, CH_2CH_3), 3.95 (s, 3 H, OCH_3), 3.96 (s, 3 H, OCH_3), 4.33 (q, $^3J_{CH2,CH3}$ = 7.2 Hz, 2 H, CH_2CH_3), 6.00 (brs, 1 H, OH, 6.79 (s, 1 H, 6-H); ^{13}C-NMR (75 MHz, $CDCl_3$): δ_C [ppm] = 14.0 (CH_2CH_3), 56.6 (OCH_3), 61.7 (OCH_3), 62.3 (CH_2CH_3), 106.4 (C-6), 115.2 (q, $^2J_{C2,F}$ = 31.3 Hz, C-2), 123.5 (q, $^1J_{CF3,F}$ = 273.6 Hz, CF_3), 124.9 (q, $^3J_{1C,F}$ = 2.8 Hz, 1-C), 140.9 (C-4), 146.1 (q, $^3J_{3C,F}$ = 1.8 Hz, C-3), 149.5 (C-5), 168.3 (*C*OO); ^{19}F-NMR (275 MHz, CDCl3): δ_F [ppm] = -56.2 (s, 3 F, CF_3); GC-MS (EI, 70 eV): *m* z^{-1} [%] = 294 [M^+] (xy), 249 [$C_{10}H_8F_3O_4^+$] (100); IR (ATR-film): $\tilde{\nu}_{max}$ [cm^{-1}] = 3231 (OH), 2983, 1691 (C=O), 1133 (C-F). HR-MS: *m* z^{-1} = 293.0643 [$(M-H)^-$] (calcd.: 293.0642).

Following the representative procedure, four reactions were run in parallel with ketone **1c** (11.3 mg mL^{-1}) as substrate using Langlois' reagent for the trifluoromethylation. Chromatography on silica afforded the product **2c** in 57.3% yield (33.7 mg, 0.115 mmol).

1-(4-Hydroxy-5-methoxy-2-(trifluoromethyl)phenyl)ethanone (2d)

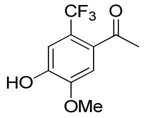

2d [x-ray]

Following the representative procedure, six reactions were run in parallel with ketone **1d** (8.30 mg mL^{-1}) as substrate and Baran's zinc sulfinate as trifluoromethylating reagent. Chromatography on silica (eluent: CH_2Cl_2) afforded trifluoromethylated arene **2d** as major isomer as colourless solid 39.3% yield (27.6 mg, 0.118 mmol). R_F (PE/EtOAc 70:30) = 0.17; melting range = 118.5-120.0 °C; ^{1}H-NMR (300 MHz, $CDCl_3$): δ_H [ppm] = 2.55 (s, 3 H, CH_3), 3.95 (s, 3 H, OCH_3), 6.06 (brs, 1 H, OH), 6.94 (s, 1 H, 6-H), 7.22 (s, 1 H, 2'-H); ^{13}C-NMR (75 MHz, $CDCl_3$): δ_C [ppm] = 30.6 (CH_3), 56.4 (OCH_3), 110.3 (C-6), 113.4 (q, $^3J_{C3,F}$ = 5.3 Hz, C-3'), 120.8 (q, $^2J_{C2,F}$ = 33.2 Hz, C-2); 124.0 (q, $^1J_{CF3,F}$ = 272.7 Hz, CF_3), 133.0 (q, $^3J_{C1,F}$ = 2.1 Hz, C-1), 147.1 (C-4), 148.4 (C-5), 201.2 (*C*O); ^{19}F-NMR (275 MHz, $CDCl_3$): δ_F [ppm] = -57.0; GC-MS (EI, 70 eV): *m* z^{-1} [%] = 234 [M^+] (34), 219 [$C_9H_6F_3O_3^+$] (100), 191 [$C_8H_6F_3O_2^+$] (18), 43 [$C_2H_3O^+$] (8); IR (ATR-film): $\tilde{\nu}_{max}$ [cm^{-1}] = 3245 (OH), 2921, 2852, 1677 (C=O), 1302, 1123 (C-F), 615. HR-MS: *m* z^{-1} = 233.0428 [$(M-H)^-$] (calcd.: 233.0431).

Following the representative procedure, four reactions were run in parallel with ketone **1d** (8.30 mg mL^{-1}) as substrate using Langlois' reagent as trifluoromethylating reagent. Chromatography on silica afforded the pure regioisomer **2d** in 29.2% yield (34.2 mg, 0.146 mmol).

Ethyl 4-hydroxy-5-methoxy-2-(trifluoromethyl)benzoate (2e-C2)

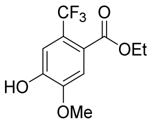

2e-C2

Following the representative procedure, ten reactions were run in parallel with ketone **1e** (9.81 mg mL^{-1}) as substrate and Baran's zinc sulfinate as trifluoromethylating reagent. Chromatography on silica (eluent: PE/EtOAc 85:15) afforded trifluoromethylated arene **2ea** as major isomer as colourless solid 29.9% yield (39.5 mg, 0.149 mmol). R_F (PE/EtOAc 70:30) = 0.33; melting range = 77.5-81.0 °C; ^{1}H-NMR (300 MHz, $CDCl_3$): δ_H [ppm] = 1.38 (t, $^3J_{CH3,CH2}$ = 7.2 Hz, 3 H, CH_2CH_3), 3.96 (s, 3 H, OCH_3), 4.37 (q, $^3J_{CH2,CH3}$ = 7.2 Hz, 2 H, CH_2CH_3), 6.06 (brs, 1 H, OH), 7.27 (s, 1 H, 6-H), 7.34 (s, 1 H, 3-H); ^{13}C-NMR (75 MHz, $CDCl_3$): δ_C [ppm] = 14.0 (CH_2CH_3), 56.5 (OCH_3), 62.1 (CH_2CH_3), 113.2 (C-6), 113.7 (q, $^3J_{C3,F}$ = 5.9 Hz, C-3), 123.3 (q, $^2J_{C2,F}$ = 33.1 Hz, C-3), 123.5 (q, $^1J_{CF3,F}$ = 272.9 Hz, CF_3), 123.5 (q, $^3J_{C1,F}$ = 2.1 Hz, C-1), 147.8 (C-4), 148.1 (C-5), 166.6 (*C*OO), ^{19}F-NMR (275 MHz, $CDCl_3$): δ_F [ppm] = -58.2 (CF_3); GC-MS (EI, 70 eV): *m* z^{-1} [%] = 264 [M^+] (34), 236 [$C_9H_7F_3O^+$] (18), 219 [$C_9H_6F_3O_3^+$] (100); IR (ATR-film): $\tilde{\nu}_{max}$ [cm^{-1}] = 3390 (OH), 2996, 1700 (C=O), 1595, 1108 (C-F). HR-MS: *m* z^{-1} = 263.0539 [$(M-H)^-$] (calcd.: 263.0537).

Ethyl 4-hydroxy-3-methoxy-5-(trifluoromethyl)benzoate (2e-C3)

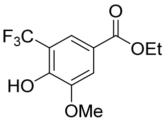

2e-C3

Compound **2eb** was obtained as colourless solid as minor isomer in 7.3% yield (9.6 mg, 0.036 mmol) from the former biotransformation (elutes first). R_F (PE/EtOAc 70:30) = 0.39, melting range = 113-114 °C; ^{1}H-NMR (300 MHz, $CDCl_3$): δ_H [ppm] = 1.49 (t, $^3J_{CH3,CH2}$ = 7.2 Hz, 3 H, CH_2CH_3), 4.00 (s, 3 H, OCH_3), 4.38 (q, $^3J_{CH2,CH3}$ = 7.1 Hz, 2 H, CH_2CH_3), 6.52 (brs, 1 H, OH), 7.69 (d, $^4J_{6,2}$ = 1.7 Hz, 1 H, 6-H), 7.90 (d, $^4J_{6,2}$ = 1.7 Hz, 1 H, 2-H); ^{13}C-NMR (75 MHz, $CDCl_3$): δ_C [ppm] = 14.5 (CH_2CH_3), 56.7 (OCH_3), 61.5 (CH_2CH_3), 114.4 (C-6), 116.1 (q, $^2J_{C3,F}$ = 32.6 Hz, C-3), 121.0 (q, $^3J_{C2,F}$ = 5.3 Hz, C-2), 122.2 (C-1), 132.2 (q, $^1J_{F,CF3}$ = 272.8 Hz, CF_3), 146.9 (C-5), 148.2 (q, $^3J_{F,C4}$ = 2.0 Hz, C-4), 165.6 (*C*OO); ^{19}F-NMR (275 MHz, $CDCl_3$): δ_F [ppm] = -62.3 (CF_3); GC-MS (EI, 70 eV): *m* z^{-1} [%] = 264 [M^+] (82), 219 [$C_9H_6F_3O_3^+$] (100); IR (ATR-film): $\tilde{\nu}_{max}$ [cm^{-1}] = 3294 (OH), 2996, 2931, 1700 (C=O), 1109 (C-F), 683. HR-MS: *m* z^{-1} = 263.0537 [$(M-H)^-$] (calcd.: 263.0537).

Following the representative procedure, ten reactions were run in parallel with ketone **1e** (9.81 mg mL^{-1}) as substrate using Langlois' reagent as trifluoromethylating reagent. Chromatography on silica afforded first regioisomer **2eb** in 7.7% (10.2 mg, 0.039 mmol) and then **2ea** in 23.6% (31.2 mg, 0.118 mmol) yield.

1-(4-Hydroxy-3-(trifluoromethyl)phenyl)ethanone (2f)

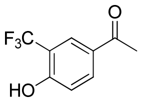

2f

Following the representative procedure, ten reactions were run in parallel with ketone **1f** (6.80 mg mL^{-1}) as substrate and Baran's zinc sulfinate as trifluoromethylating reagent. Chromatography on silica (eluent: CH_2Cl_2/EtOAc 96:4) afforded the product **2f** as solid 31.7% yield (32.4 mg, 0.159 mmol). R_F (PE/EtOAc 75:25) = 0.18; melting range = 175-176 °C; ^{1}H-NMR (300 MHz, $CDCl_3$): δ_H [ppm] = 2.52 (s, 3 H, CH_3), 6.99 (d, $^3J_{5,6}$ = 8.60 Hz, 1 H, 5-H), 8.03 (dd, $^3J_{6,5}$ = 8.6 Hz, $^4J_{6,2}$ = 1.3 Hz, 1 H, 6-H), 8.11 (d, $^4J_{2,6}$ = 1.3 Hz, 1 H, 2-H); ^{13}C-NMR (75 MHz, $CDCl_3$): δ_C [ppm] = 26.3 (CH_3), 117.7 (C-5), 118.1 (q, $^2J_{C3,F}$ = 31.2 Hz, C-3), 124.9 (q, $^1J_{F,CF3}$ = 271.4 Hz, CF_3), 128.9 (q, $^3J_{C2,F}$ = 5.3 Hz, C-2), 129.6 (C-1), 135.2 (C-6), 161.7 (q, $^3J_{C4,F}$ = 1.6 Hz, C-4), 198.1 (*C*OCH_3); ^{19}F-NMR (275 MHz, $CDCl_3$): δ_F [ppm] = -64.5 (CF_3); GC-MS (EI, 70 eV): $m\ z^{-1}$ [%] = 204 [M^+] (39), 189 [$C_8H_4F_3O_2^+$] (77), 169 [$C_9H_7F_2O^+$] (100); IR (ATR-film): $\tilde{\nu}_{max}$ [cm^{-1}] = 3163 (OH), 2379, 1663 (C=O), 1595, 1133 (C-F), 823, 573. HR-MS: $m\ z^{-1}$ = 203.0327 [$(M-H)^-$] (calcd.: 203.0325).

Following the representative procedure, ten reactions were run in parallel with ketone **1f** (6.80 mg mL^{-1}) as substrate using Langlois' reagent as trifluoromethylating reagent. Chromatography on silica afforded the product **2f** as colourless solid in 30.5% yield (31.1 mg, 0.152 mmol).

4-hydroxy-3-(trifluoromethyl)benzonitrile (2g)

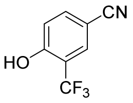

2g

Following the representative procedure, eight reactions were run in parallel with 4-cyanophenol (**1g**) (5.96 mg mL^{-1}) as substrate and Baran's zinc sulfinate as trifluoromethylating reagent.

Chromatography on silica (eluent: PE/EtOAc 80:20) afforded the product **2g** as yellowish oil in 56.7% yield (52.8 mg, 0.282 mmol). R_F (PE/EtOAc 70:30) = 0.50; ^{1}H-NMR (300 MHz, $CDCl_3$): δ_H [ppm] = 7.28 (d, $^3J_{5,6}$ = 8.6 Hz, 1 H, 5-H), 7.88 (dd, $^3J_{6,5}$ = 8.6 Hz, $^4J_{6,2}$ = 2.2 Hz, 1 H, 6-H), 8.01 (d, $^4J_{2,6}$ = 2.1 Hz, 1 H, 2-H), 10.62 (brs, 1 H, OH). ^{13}C-NMR (75 MHz, $CDCl_3$): δ_C [ppm] = 102.9 (C-1), 117.6 (q, $^2J_{C3,F}$ = 31.8 Hz, C-3), 117.7 (CN), 118.2 (C-5), 123.0 (q, $^1J_{CF3,F}$ = 271.9 Hz, CF_3), 131.6 (q, $^3J_{C2,F}$ = 4.7 Hz, C-2), 137.6 (C-6), 159.4 (q, $^3J_{C4,F}$ = 1.8 Hz, C-4); ^{19}F-NMR (272 MHz, $CDCl_3$): δ_F [ppm] = -63.70 (s, 3 F, CF_3); GC-MS (EI, 70 eV): *m* z^{-1} [%] = 63 (10), 75 (12), 88 (15), 139 (100), 167 [$C_8H_3F_2NO^+$] (85), 187 [M^+] (56); HR-MS: *m* z^{-1} = 186.0171 [$(M-H)^-$] (calcd.: 186.0172).

2,6-dimethoxy-3,4-bis(trifluoromethyl)phenol (2h)

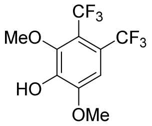

2h

Following the representative procedure, eight reactions were run in parallel with ketone (2,6-dimethoxyphenol **1h**) (7.71 mg mL^{-1}) as substrate and Baran's zinc sulfinate as trifluoromethylating reagent. Chromatography on silica (eluent: PE/EtOAc 80:20) afforded the product **2h** as colourless solid in 33.1% yield (38.4 mg, 0.132 mmol). RF (PE/EtOAc 70:30) = 0.52; melting range = 152-158 °C; ^{1}H-NMR (300 MHz, $CDCl_3$): δ_H [ppm] = 3.97 (s, 3 H, OCH_3), 3.99 (s, 3 H, OCH_3), 7.08 (s, 1 H, 5-H); ^{13}C-NMR (75 MHz, $CDCl_3$): δ_C [ppm] = 56.5 (OMe), 61.6 (OMe), 105.4 (q, $^3J_{C5,F}$ = 7.3 Hz, C-5), 116.4 (q, $^2J_{C3,F}$ = 32.3 Hz, C-3), 119.8 (q, $^2J_{C4,F}$ = 33.6 Hz, C-4), 122.5 (q, $^1J_{CF3,F}$ = 273.5 Hz, CF_3), 122.9 (q, $^1J_{CF3,F}$ = 272.5 Hz, CF_3), 142.1 (C-1), 147.4 ($^3J_{C2,F}$ = 2.0 Hz, C-2), 148.7 (C-6); ^{19}F-NMR (272 MHz, $CDCl_3$): δ_F [ppm] = -55.82 (q, $^5J_{F,F}$ = 15.5 Hz), -57.20 (q, $^5J_{F,F}$ = 15.5 Hz); GC-MS (EI, 70 eV): *m* z^{-1} [%] = retention time 7.5 min: 68 (14), 99 (17), 137 (10), 156 (22), 199 (15), 221 (10), 227 (76), 251 [$C_{10}H_7F_4O_3^+$] (12), 255 (30), 271 [$C_{10}H_7F_5O_3^+$] (23), 290 [M^+] (100); retention time 6.9: 83 (10), 88 (16), 116 (11), 159 (83), 187 (23), 203 [$C_9H_8F_2O_3^+$] (11), 222 [M^+] (100); retention time 6.7: 133 (30), 136 (11), 161 (14), 164 (20), 179 (17), 203 [$C_9H_8F_2O_3^+$] (17), 207 (35), 222 [M^+] (100); HR-MS: *m* z^{-1} = 289.0303 [$(M-H)^-$] (calcd.: 289.0305).

5. Energy Diagrams

Transition states were calculated for the reaction of the CF_3 radical with the phenol radical cation to give the corresponding substituted phenol cation (cationic intermediate) (Supplementary Scheme 1). Energies were calculated for the transition state and optimised structures of the cationic intermediate. For graphical representation of the reaction scheme and energy diagram please see Supplementary Figure 4, Supplementary Figure 5 and Supplementary Figure 6. For Energies and free energy corrections values see Supplementary Table 5 and Supplementary Table 6. Coordinates and energies of the transitions states can be found in Supplementary Table 7–9.

6. Chemical Trifluoromethylations Reactions

6.1. Silver Catalyzed Trifluoromethylation

The trifluoromethylation of phenols **1a**-**b** was investigated under silver catalysed conditions according to a literature procedure[3] (Supplementary Table S6). For both substrates, the reaction reached high conversions after 4 h, however isomeric mixtures of *O*- and *C*-trifluoromethylation with a slight preference for the *O*-functionalization were observed.

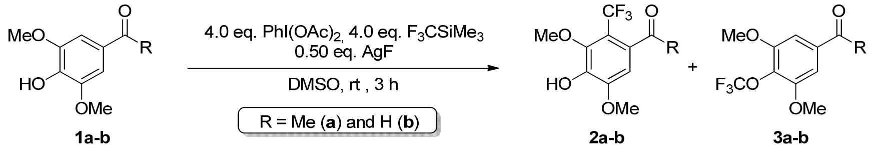

For results see Supplementary Table 10.

6.1.1. Representative Procedure: Silver Catalysed Trifluoromethylation

To a flame dried vial containing phenol **1a**-**b** (0.15 mmol) and $PhI(OAc)_2$ (193.3 mg, 0.60 mmol, 4 eq.) was added DMSO (dry, 1 mL) and F_3CSiMe_3 (88.6 µL, 0.60 mmol, 4 eq.) and the suspension was vigorously stirred at room temperature for 1 min. AgF (9.3 mg, 0.075 mmol, 0.50 eq.) was then slowly added in portions and the reaction was stirred at room temperature for further 4 h. The reaction was quenched with H_2O (10 mL) and extracted with Et_2O (3×10 mL). The organic layers were combined, dried over Na_2SO_4 the solvent removed by distillation under reduced pressure and the residue purified by flash chromatography (30-60% EtOAc/PE for **3a** and 10-30% EtOAc/PE for **3b**).

1-[3,5-Dimethoxy-4-(trifluoromethoxy)phenyl]ethanone (3a). Isolated yield: 17% (20.1 mg, 76 µmol). Colourless solid. R_F (PE/EtOAc 80:20) = 0.30; m.p.: 55.0-55.4 °C; ^{1}H-NMR (300 MHz, $CDCl_3$): δ_H [ppm] = 2.63 (s, 3 H), 3.95 (s, 6 H), 7.23 (s, 2 H), ^{13}C-NMR (75.5 MHz, $CDCl_3$): δ_C [ppm] = 26.6 (CH_3),

56.5 (2 CH_3), 105.1 (2 CH), 120.7 (q, $^{1}J_{CF}$ = 264.5 Hz), 130.9 (C), 136.1 (C), 153.7 (C), 196.7 (C). ^{19}F-NMR (282 MHz, $CDCl_3$): δ_F [ppm] = -57.9 (OCF_3); GC-MS (EI, 70 eV): *m* z^{-1} [%] = 264 [M^+] (54), 249 [M-CH_3^+] (100); IR (ATR-film): $\tilde{\nu}_{max}$ [cm^{-1}] = 1682, 1601 (C=O), 1411, 1335, 1164, 1122, 1035.

3,5-Dimethoxy-4-(trifluoromethoxy)benzaldehyde (3b). Isolated yield: 13% (19.6 mg, 78 µmol). Colourless solid. R_F (PE/EtOAc 90:10) = 0.14, melting point = 71.2-71.4 °C; ^{1}H-NMR (300 MHz, $CDCl_3$): δ_H [ppm] = 3.97 (s, 6 H), 7.17 (s, 2 H), 9.95 (s, 1 H). ^{13}C-NMR (75.5 MHz, $CDCl_3$): δ_C [ppm] = 56.5 (2 CH_3), 106.0 (2 CH), 120.6 (q, $^{1}J_{CF}$ = 260.2 Hz), 131.7 (C), 135.1 (C), 154.4 (C), 190.8 (C). ^{19}F-NMR (282 MHz, $CDCl_3$): -57.8 (OCF_3). GC-MS (EI, 70 eV): *m* z^{-1} [%] = 250 [M^+] (100). IR (ATR-film): $\tilde{\nu}_{max}$ [cm^{-1}] = 1701 (C=O), 1602, 1498, 1335, 1121.

Determination of conversion by achiral GC-measurement from the crude reaction mixture: HP-5 column (Agilent Technologies), temperature program: 80-220 °C, slope = 10 °C min^{-1}, then with 20°C/min to 280°C. R_t (**1a**) = 21.1 min, R_t (**1b**) = 19.4 min, R_t (**3a**) = 15.5 min, R_t (**3b**) = 13.5 min, R_t (**2a**) = 19.6 m in, R_t (**2b**) = 17.1 min.

6.2. Transition Metal Free Trifluoromethylation

The metal free trifluoromethylation of the phenols **1a**-**b** was conducted under oxidative conditions by the combination of the Langlois' reagent ($NaSO_2CF_3$) and phenyl iodine bis(trifluoroacetate) according to a literature procedure.[4]

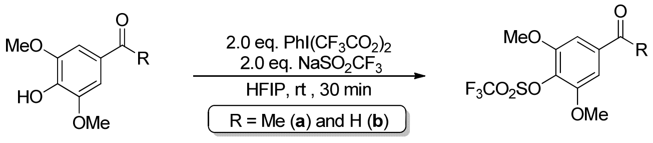

1a-b **4a-b**

For results see Supplementary Table 11.

Determination of conversion by achiral GC-measurement from the crude reaction mixture: HP-5 column (Agilent Technologies), temperature program: 80-220 °C, slope = 5 °C min^{-1}, then with 20°C/min to 280°C. R_t (**1a**) = 21.1 min, R_t (**1b**) = 19.4 min, R_t (**4a**) = 19.0 min, R_t (**4b**) = 20.7 min.

6.2.1. Representative Procedure: Metal Free Trifluoromethylation

The phenols **1a**-**b** (0.20 mmol) and $NaSO_2CF_3$ (62.4 mg, 0.40 mmol, 2.0 eq.) were suspended in hexafluoro-2-propanol (HFIP, 0.4 mL) and $PhI(CF_3CO_2)_2$ (172.2 mg, 0.40 mmol, 2 eq.) was added. The mixture was vigorously stirred at room temperature for 30 min. The reaction was quenched with H_2O (10 mL) and extracted with EtOAc (3 × 10 mL). The organic layers were combined, dried over Na_2SO_4, the solvent removed by distillation under reduced pressure and residue purified by flash chromatography (20% EtOAc/PE).

4-Acetyl-2,6-dimethoxyphenyl trifluoromethanesulfonate (**4a**). Isolated yield: 18% (23.4 mg, 70 µmol). Colourless solid. R_F (PE/EtOAc 90:10): 0.19; melting point = 73.5-74.0 °C; ^{1}H-NMR (300 MHz, $CDCl_3$): δ_H [ppm] = 2.64 (s, 3 H), 3.98 (s, 6 H), 7.24 (s, 2 H), 9.95 (s, 1 H). ^{13}C-NMR (75.5 MHz, $CDCl_3$): δ_C [ppm] = 26.6 (CH_3), 56.5 (2 CH_3), 104.9 (2 CH), 118.6 (q, $^1J_{CF}$ = 320.1 Hz), 131.2 (C), 136.8 (C), 152.5 (C), 196.3 (C). ^{19}F-NMR (282 MHz; $CDCl_3$): δ_F [ppm] = -73.6 (OSO_2CF_3). GC-MS (EI, 70 eV): $m\ z^{-1}$ [%] = 328 [M^+] (22), 195 [$M-SO_2CF_3^+$] (100). IR (ATR-film): $\tilde{\nu}_{max}$ [cm^{-1}] = 1687 (C=O), 1607, 1414, 1360, 1286

4-Formyl-2,6-dimethoxyphenyl trifluoromethanesulfonate (4b). Isolated yield: 89% (112 mg, 356 µmol). Colourless solid. R_F (PE/EtOAc 60:40): 0.40; melting point: 96.5-97.0 °C; ^{1}H-NMR (300 MHz, $CDCl_3$): δ_H [ppm] = 4.00 (s, 6 H), 7.19 (s, 2 H), 9.95 (s, 1 H), ^{13}C-NMR (75.5 MHz, $CDCl_3$): δ_C [ppm] = 56.7 ($2CH_3$), 105.9 (2 CH), 118.6 (q, $^1J_{CF}$ = 320.0 Hz), 132.0 (C), 135.8 (C), 153.2 (C), 190.5 (C). ^{19}F-NMR (282 MHz; $CDCl_3$): δ_F [ppm] = -73.5 (OSO_2CF_3). GC-MS (EI, 70 eV): $m\ z^{-1}$ [%] = 314 [M^+] (27), 181 [$M-SO_2CF_3^+$] (100). IR (ATR-film): $\tilde{\nu}_{max}$ [cm^{-1}] = 1701 (C=O), 1602, 1498, 1335, 1121

6.3. Electrophilic trifluoromethylation using the Togni-reagent

The trifluoromethylation using the Togni-reagent (3,3-Dimethyl-1-(trifluoromethyl)-1,2-benziodoxole) was tested on phenol **1a** according to a literature procedure.[5]

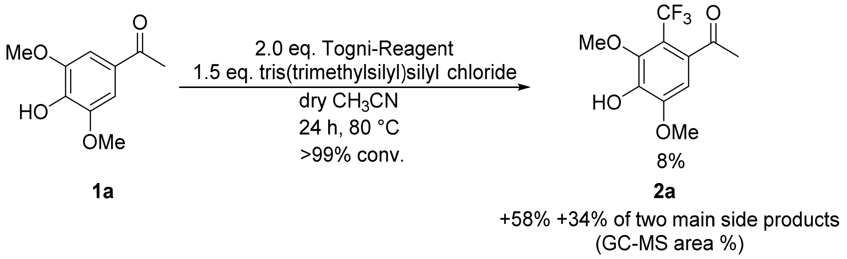

6.3.1. Representative Procedure: Electrophilic trifluoromethylation

3,3-Dimethyl-1-(trifluoromethyl)-1,2-benziodoxole (200 mg, 0.6 mmol), tris(trimethylsilyl)silyl chloride (125 mg, 0.45 mmol) and phenol **1a** (59 mg, 0.3 mmol) were dissolved in dry CH_3CN (2 mL) and the mixture was to stirred for at 80°C 24 h. The crude mixture was diluted with CH_3CN before analysis. Analysis was performed by GC-MS on an achiral stationary phase showing that the substrate was completely converted but just leading to product **2a** with 8%, while two unidentified products were the main products.

For results see Supplementary Table 12.

7. X-Ray Diffraction Data

7.1. Data for 4-hydroxy-3,5-dimethoxy-2-(trifluoromethyl)benzaldehyde (2b)

The crystal structure has been deposited at the Cambridge Crystallographic Data Centre and allocated the deposition number CCDC 1480621.

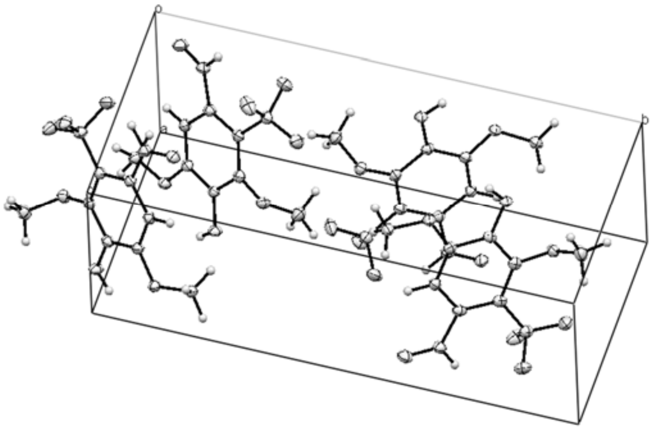

Packing (two molecules in the asymmetric unit)

Crystal data and structure refinement for 4-hydroxy-3,5-dimethoxy-2-(trifluoromethyl)-benzaldehyde (**2b**) can be found in Supplementary Table 13.

Bond lengths [A] and angles [deg] for4-hydroxy-3,5-dimethoxy-2-(trifluoromethyl)-benzaldehyde (**2b**) can be found in Supplementary Table 14.

Torsion angles [deg] for 4-hydroxy-3,5-dimethoxy-2-(trifluoromethyl)benzaldehyde (**2b**) can be found in Supplementary Table 15.

7.2. Data for ethyl 4-hydroxy-3,5-dimethoxy-2-(trifluoromethyl)benzoate (2c)

The crystal structure has been deposited at the Cambridge Crystallographic Data Centre and allocated the deposition number CCDC 1480623.

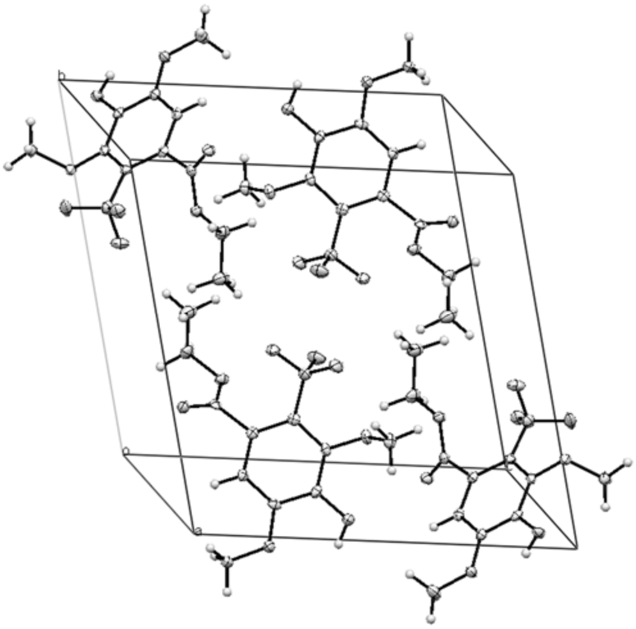

Packing (two independent molecules in the asymmetric unit)

Crystal data and structure refinement for ethyl 4-hydroxy-3,5-dimethoxy-2-(trifluoro-methyl)benzoate (**2c**) can be found in Supplementary Table 16.

Bond lengths [A] and angles [deg] ethyl 4-hydroxy-3,5-dimethoxy-2-(trifluoro-methyl)benzoate (**2c**) can be found in Supplementary Table 17.

Torsion angles [deg] for ethyl 4-hydroxy-3,5-dimethoxy-2-(trifluoro-methyl)benzoate (**2c**) can be found in Supplementary Table 18.

7.3. Data for 1-(4-hydroxy-5-methoxy-2-(trifluoromethyl)phenyl)ethanone (2d)

The crystal structure has been deposited at the Cambridge Crystallographic Data Centre and allocated the deposition number CCDC 1480622.

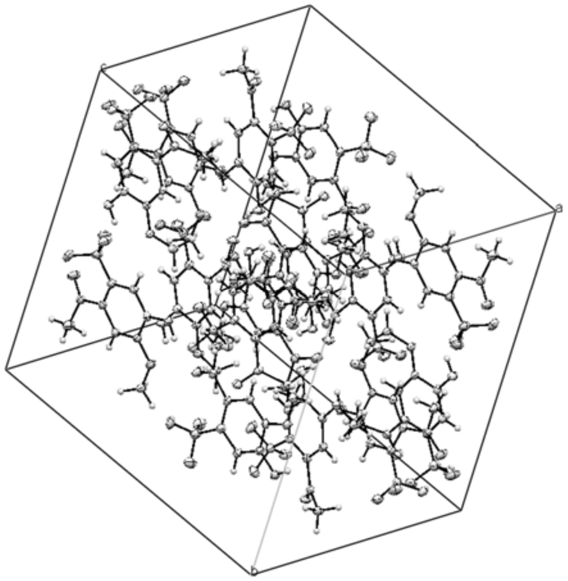

Packing (two independent molecules in the asymmetric unit)

Crystal data and structure refinement for 1-(4-hydroxy-5-methoxy-2-(trifluoromethyl)-phenylethanone (**2d**) can be found in Supplementary Table 19.

Bond lengths [A] and angles [deg] 1-(4-hydroxy-5-methoxy-2-(trifluoromethyl)-phenylethanone (**2d**)can be found in Supplementary Table 20.

Torsion angles [deg] for 1-(4-hydroxy-5-methoxy-2-(trifluoromethyl)-phenylethanone (**2d**) can be found in Supplementary Table 21.

Supplementary references

1. Wan, Y.-Y., Lu, R., Xiao, L., Du, Y.-M., Miyakoshi, T., Chen, C.-H., Knill, C. J., Kennedy, J. F. Effects of organic solvents on the activity of free and immobilised laccase from *Rhus vernicifera. Int. J. Biol. Macromolec.* **47**, 488-495 (2010).
2. Kallio, J. P., Auer, S., Jänis, J., Andberg, M., Kruus, K., Rouvinen, J., Koivula, A., Hakulinen, N. Structure-Function Studies of a *Melanocarpus albomyces* Laccase Suggest a Pathway for Oxidation of Phenolic Compounds. *J. Mol. Biol.* **392**, 895-909 (2009).
3. Seo, S., Taylor, J. B., Greaney, M. F. Silver-catalysed trifluoro-methylation of arens at room temperature. *Chem. Commun.* **49**, 6385-6387 (2013).
4. Yang, Y.-D., Iwamoto, K., Tokunaga, E., Shibata, N. Transition-metal-free oxidative trifluoromethylation of unsymmetrical biaryls with trifluoromethanesulfinate. *Chem. Commun.* **49**, 5510-5512 (2013).
5. Wiehn, M. S., Vinogradova, E. V. Togni, A. Electrophilic trifluoromethylation of arenes and N-heteroarenes using hypervalent iodine reagents. *J. Fluorine Chem*. **131**, 951-957 (2010).
